# Bioinspired Pyrano[2,3-*f*]chromen-8-ones: Ring C-Opened Analogues of Calanolide A: Synthesis and Anti-HIV-1 Evaluation

**DOI:** 10.3390/biomimetics9010044

**Published:** 2024-01-11

**Authors:** Igor A. Khalymbadzha, Ramil F. Fatykhov, Ilya I. Butorin, Ainur D. Sharapov, Anastasia P. Potapova, Nibin Joy Muthipeedika, Grigory V. Zyryanov, Vsevolod V. Melekhin, Maria D. Tokhtueva, Sergey L. Deev, Marina K. Kukhanova, Nataliya N. Mochulskaya, Mikhail V. Tsurkan

**Affiliations:** 1 Department of Organic and Biomolecular Chemistry, Ural Federal University, 620002 Yekaterinburg, Russia; rf.fatykhov@urfu.ru (R.F.F.); iibutorin@urfu.ru (I.I.B.); a.d.sharapov@urfu.ru (A.D.S.); a.p.potapova@urfu.ru (A.P.P.); mnibinjoy@gmail.com (N.J.M.); g.v.zyrianov@urfu.ru (G.V.Z.); v.v.melekhin@urfu.ru (V.V.M.); maria.tokhtueva@urfu.ru (M.D.T.); deevsl@yandex.ru (S.L.D.); n.n.mochulskaia@urfu.ru (N.N.M.); 2 Department of Medical Biology and Genetics, Ural State Medical University, 620028 Yekaterinburg, Russia; 3 Engelhardt Institute of Molecular Biology, 119991 Moscow, Russia; kukhan86@hotmail.com; 4 Leibniz Institute of Polymer Research Dresden, 01069 Dresden, Germany

**Keywords:** Calanolide A, anti-HIV activity, reverse transcriptase, non-nucleoside reverse transcriptase inhibitors (NNRTIs), Mannich reaction, CH/CH-coupling

## Abstract

We have designed and synthesized a series of bioinspired pyrano[2,3-*f*]coumarin-based Calanolide A analogs with anti-HIV activity. The design of these new calanolide analogs involved incorporating nitrogen heterocycles or aromatic groups in lieu of ring C, effectively mimicking and preserving their bioactive properties. Three directions for the synthesis were explored: reaction of 5-hydroxy-2,2-dimethyl-10-propyl-2*H*,8*H*-pyrano[2,3-*f*]chromen-8-one with (i) 1,2,4-triazines, (ii) sulfonylation followed by Suzuki cross-coupling with (het)aryl boronic acids, and (iii) aminomethylation by Mannich reaction. Antiviral assay of the synthesized compounds showed that compound **4** has moderate activity against HIV-1 on enzymes and poor activity on the cell model. A molecular docking study demonstrates a good correlation between in silico and in vitro HIV-1 reverse transcriptase (RT) activity of the compounds when docked to the nonnucleoside RT inhibitor binding site, and alternative binding modes of the considered analogs of Calanolide A were established.

## 1. Introduction

Acquired immunodeficiency syndrome (AIDS) and the human immunodeficiency virus (HIV) that causes it remain a major global public health problem, claiming approximately 40.4 million lives. In 2022, approximately 630,000 people will die from HIV-related diseases, and 1.3 million people will become infected with HIV [1].

HIV-1 reverse transcriptase inhibitors (RTIs) represent a critical component of the arsenal of highly active antiretroviral therapy proposed by David Ho in 1996 [2]. However, the efficacy of RTIs has been significantly reduced due to the emergence of resistant strains of HIV-1 and side effects [3]. As a result, medicinal chemists are constantly researching new HIV-1 RTIs with increased potency, improved pharmacokinetic properties, and reduced side effects [3].

Heterocyclic compounds occupy a privileged position for the search for novel anti-HIV agents. At least 9 out of 10 currently used anti-AIDS drugs are nitrogen-containing heterocyclic compounds [4]. The important role of heterocyclic compounds, especially quinoline [5] and pyrimidine [6] derivatives in the development of new anti-HIV drugs [7] is associated with their stability, conformational rigidity, convenience of synthesis [8,9], as well as the widest possibilities for tuning the properties by changing the size of cycles, types and number of heteroatoms, etc. [10].

**Figure 1:**
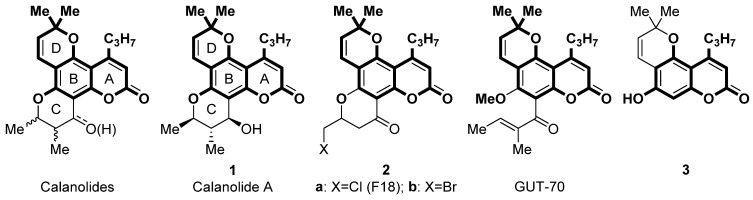
Structure of calanolides, Calanolide A (**1**), its analogues (**2a**,**b**), and 5-hydroxy-2,2-dimethyl-10-propyl-2*H*,8*H*-pyrano[2,3-*f*]chromen-8-one (**3**).

Calanolides (Figure 1) are a class of tetracyclic pyranocoumarin HIV-1 nonnucleoside RTIs (NNRTIs) isolated from a tropical tree *Calophyllum lanigerum*. Among them, Calanolide A (Figure 1) was identified as the most promising compound with an IC_50_ in the nanomolar range [11,12]. In addition, Calanolide A showed activity against a wide range of HIV-1 strains, including azidothymidine and pyridinone resistant strains [11]. Calanolide A successfully has passed Phase I clinical trials and demonstrated good tolerability in healthy volunteers [13]. However, further clinical trials were suspended due to the difficulties associated with the isolation of Calanolide A from natural sources and total synthesis [14,15], as well as an insufficiently high therapeutic index.

The structure of calanolides consists of four rings: A, B, C, and D (Figure 1). It is important to note that the activity of the modified Calanolide A derivatives depends dramatically on the C ring structure, while the substituents in other rings are less significant [11,12,16]. For example, the most active calanolide analog F18 **2a** and its bromo counterpart **2b** (Figure 1) have a modified C ring [17]. These compounds are characterized by good therapeutic indexes, 1417 and more than 10526 for compounds **2a** and **2b**, respectively, [18] as well as fairly simple synthesis, demonstrating the promise of modifying the C ring to optimize the properties of calanolide A. Another example of C ring-modified calanolide A derivatives is GUT-70 (Figure 1). This tricyclic coumarin has multiple anti-HIV actions: it inhibits the HIV-1 entry process by reducing cell membrane fluidity and down-regulates the expression of chemokine receptors CXCR4, CD4, and CCR5 [19], as well as an inhibitory effect on viral replication through the suppression of NF-κB [20].

Moreover, from a medicinal chemistry point of view, modification of the C ring is the most attractive direction since it reduces the number of chiral centers and simplifies the synthesis. While calanolide A contains 3 stereocenters and thus has 8 stereoisomers, compounds **2a**,**b** contain one stereocenter and are represented by two stereoisomers.

5-Hydroxy-2,2-dimethyl-10-propyl-2*H*,8*H*-pyrano[2,3-*f*]chromen-8-one **3**, a scaffold contained in the rings A, B, and D of Calanolide A, is a convenient starting point for the synthesis of its analogues modified at the ring C. Previously, we proposed a convenient method for the synthesis of the compound **3** based on the selective acylation of 5,7-dihydroxy-4-propylcoumarin with 1-nicotinoylbenzotriazole [21]. This method makes the tricyclic precursor **3** available in a gram scale, which we needed for our study.

With this in mind, we designed a series of bioinspired pyrano[2,3-*f*]coumarin-based Calanolide A analogs. Three directions of modification of **3** were investigated: reaction with 1,2,4-triazines, Suzuki cross-coupling reaction of sulfonylated **3** with boronic acids, and Mannich reaction with formaldehyde and cyclic aliphatic amines.

## 2. Materials and Methods

### 2.1. Chemistry

All chemicals were purchased from commercial suppliers and used as delivered. ^1^H NMR, ^13^C NMR and ^19^F NMR spectra were recorded at 400 (or 600), 100 (or 150) and 376 (or 565) MHz, respectively. Chemical shifts are reported in parts per million (ppm) and coupling constants in Hertz (Hz). Residual solvent peaks in DMSO-d_6_ (δ = 2.50 ppm) [22] and CDCl_3_ (δ = 7.26 ppm) served as internal standards for recording. Microanalyses were performed on PerkinElmer Series II CHNS/O 2400 elemental analyzer. Melting points were determined using a Stuart SMP 3 apparatus. Thin-layer chromatography (TLC) was performed using Merck silica gel 60 F_254_ TLC plates.

Images of ^1^H and ^13^C NMR spectra are provided in the Supplementary Materials.

5-Hydroxy-2,2-dimethyl-10-propyl-2*H*,8*H*-pyrano[2,3-*f*]chromen-8-one **3** was prepared from 5,7-dihydroxy-4-propylcoumarin according to the published procedure [21].

#### 2.1.1. Synthesis of 6-Triazyninyl Derivatives of 5-Hydroxy-2,2-dimethyl-10-propyl-2*H*,8*H*-pyrano[2,3-*f*]chromen-8-ones **4a**–**h** and **5a**–**f**

##### General Procedure for the Synthesis of Dihydrotriazines **4a**–**h**

To a solution of 5-hydroxy-2,2-dimethyl-10-propyl-2*H*,8*H*-pyrano[2,3-*f*]chromen-8-one **3** (1.0 mmol, 286 mg) and 1,2,4-triazines **6a**–**h** [23] (1.1 mmol) in acetic acid (10 mL) methanesulfonic acid (3.0 mmol, 288 mg) was added. The reaction mixture was allowed to stand for 24 h and then poured in saturated aqueous NaHCO_3_. The precipitate was collected and recrystallized from acetonitrile to give pure **4a**–**h**. Images of ^1^H and ^13^C NMR spectra of compounds **4a**–**h** are provided in Supplementary Figures S1–S14.

5-Hydroxy-2,2-dimethyl-6-(3-(methylthio)-2,5-dihydro-1,2,4-triazin-5-yl)-10-propyl-2*H*,8*H*-pyrano[2,3-*f*]chromen-8-one **4a**. Yield 363 mg (88%). Beige solid, m.p. 208–210 °C dec. ^1^H NMR (400 MHz, CDCl_3_): δ = 12.93 (br s, 1H), 9.91 (s, 1H), 6.75 (s, 1H), 6.69 (d, *J* = 10.0 Hz, 1H), 5.98 (s, 1H), 5.55 (d, *J* = 10.0 Hz, 1H), 4.93 (s, 1H), 2.87–3.00 (m, 2H), 1.67–1.72 (m, 2H), 1.51 (s, 3H), 1.50 (s, 3H), 1.04–1.07 (m, 3H). ^13^C NMR (101 MHz, CDCl_3_): δ = 161.7, 159.6, 158.4, 158.1, 153.1, 151.5, 141.6, 126.8, 116.8, 109.6, 107.5, 103.0, 100.7, 77.8, 55.5, 38.9, 28.0, 27.9, 23.4, 14.4, 14.2. Anal. Calcd for C_21_H_23_N_3_O_4_S: C, 61.00; H, 5.61; N, 10.16; S, 7.75. Found: C, 60.87; H, 5.69; N, 10.31; S, 7.90.

6-(3-(Butylthio)-2,5-dihydro-1,2,4-triazin-5-yl)-5-hydroxy-2,2-dimethyl-10-propyl-2*H*,8*H*-pyrano[2,3-*f*]chromen-8-one **4b**. Yield 319 mg (70%). Beige solid, m.p. 184–186 °C dec. ^1^H NMR (400 MHz, CDCl_3_): δ = 12.92 (br s, 1H), 9.95–9.98 (m, 1H), 6.73 (s, 1H), 6.70 (d, *J* = 10.0 Hz, 1H), 5.98 (s, 1H), 5.54 (d, *J* = 10.0 Hz, 1H), 4.91 (s, 1H), 3.06–3.11 (m, 1H), 2.91–3.01 (m, 3H), 1.65–1.72 (m, 4H), 1.51 (s, 3H), 1.50 (s, 3H), 1.41–1.47 (m, 2H), 1.03–1.07 (m, 3H), 0.91–0.94 (m, 3H). ^13^C NMR (101 MHz, CDCl_3_): δ = 161.7, 159.5, 158.2, 158.0, 153.1, 151.5, 141.5, 126.7, 116.9, 109.5, 107.5, 102.9, 100.8, 77.8, 55.3, 38.8, 31.8, 31.4, 28.0, 27.9, 23.4, 22.0, 14.2, 13.7. Anal. Calcd for: C_24_H_29_N_3_O_4_S: C, 63.28; H, 6.42; N, 9.22; S, 7.04. Found: C, 63.17; H, 6.61; N, 9.01; S, 6.89.

6-(3-(But-2-yn-1-ylthio)-2,5-dihydro-1,2,4-triazin-5-yl)-5-hydroxy-2,2-dimethyl-10-propyl-2*H*,8*H*-pyrano[2,3-*f*]chromen-8-one **4c**. Yield 203 mg (45%). Beige solid, m.p. 162–164 °C dec. ^1^H NMR (400 MHz, CDCl_3_): δ = 12.32 (br s, 1H), 9.92 (s, 1H), 6.71 (s, 1H), 6.69 (d, *J* = 10 Hz, 1H), 5.98 (s, 1H), 5.54 (d, *J* = 10 Hz, 1H), 4.94 (s, 1H), 3.78 (dq, *J* = 2.7, 16.2 Hz, 1H), 3.66 (dq, *J* = 2.7, 16.2 Hz, 1H), 1.87–2.99 (m, 2H), 1.85 (t, *J* = 2.76 Hz, 3H), 1.65–1.74 (m, 2H), 1.51 (s, 3H), 1.50 (s, 3H), 1.04–1.07 (m, 3H). ^13^C NMR (101 MHz, CDCl_3_): δ = 161.7, 159.6, 157.8, 156.9, 153.1, 151.5, 141.8, 126.8, 116.9, 109.6, 107.5, 103.0, 100.9, 80.6, 77.8, 73.4, 55.3, 38.9, 28.03, 27.97, 23.4, 21.3, 14.2, 3.9. Anal. Calcd for C_24_H_25_N_3_O_4_S: C, 63.84; H, 5.58; N, 9.31; S, 7.10. Found: C, 63.92; H, 5.41; N, 9.23; S, 7.10.

6-(3-(Benzylthio)-2,5-dihydro-1,2,4-triazin-5-yl)-5-hydroxy-2,2-dimethyl-10-propyl-2*H*,8*H*-pyrano[2,3-*f*]chromen-8-one **4d**. Yield 318 mg (65%). Beige solid, m.p. 183–184 °C dec. ^1^H NMR (400 MHz, CDCl_3_): δ = 12.73 (s, 1H), 9.98 (s, 1H), 7.33–7.35 (m, 2H), 7.23–7.31 (m, 3H), 6.70 (d, *J* = 10.0 Hz), 6.68 (s, 1H), 5.97 (s, 1H), 5.56 (d, *J* = 10 Hz, 1H), 4.90 (s, 1H), 4.36 (d, *J* = 13.1 Hz, 1H), 4.18 (d, *J* = 13.1 Hz, 1H), 2.91–2.96 (m, 2H), 1.67–1.72 (m, 2H), 1.51 (s, 3H), 1.50 (s, 3H), 1.04–1.07 (m, 3H). ^13^C NMR (101 MHz, CDCl_3_): δ = 161.7, 159.6, 158.0, 157.2, 153.0, 151.5, 141.6, 135.7, 129.1, 128.8, 127.9, 126.8, 116.8, 109.6, 107.5, 103.0, 100.8, 77.8, 55.3, 38.8, 36.7, 28.1, 27.9, 23.4, 14.2. Anal. Calcd for C_27_H_27_N_3_O_4_S: C, 66.24; H, 5.56; N, 8.58; S, 6.55. Found: C, 66.25; H, 5.51; N, 8.42; S, 6.60.

5-Hydroxy-2,2-dimethyl-6-(3-(phenylthio)-2,5-dihydro-1,2,4-triazin-5-yl)-10-propyl-2*H*,8*H*-pyrano[2,3-*f*]chromen-8-one **4e**. Yield 342 mg (72%). Beige solid, m.p. 185–187 °C. ^1^H NMR (400 MHz, DMSO-d_6_): δ = 12.32 (br s, 1H), 11.82 (s, 1H), 7.59–7.61 (m, 2H), 7.48–7.50 (m, 3H), 6.94 (s, 1H), 6.43 (d, *J* = 10.0 Hz, 1H), 5.93 (s, 1H), 5.63 (d, *J* = 10.0 Hz, 1H), 4.62 (s, 1H), 2.81–2.85 (m, 2H), 1.54–1.60 (m, 2H), 1.42 (s, 6H), 0.96–1.00 (m, 3H). ^13^C NMR (101 MHz, DMSO-d_6_): δ = 159.1, 157.6, 156.6, 156.4, 152.8, 150.4, 141.0, 134.7, 129.9, 129.7, 127.4, 127.0, 115.9, 109.6, 106.2, 101.9, 100.4, 77.4, 54.8, 37.7, 27.3, 27.1, 23.0, 13.8. Anal. Calcd for C_26_H_25_N_3_O_4_S: C, 65.67; H, 5.30; N, 8.84; S, 6.74. Found: C, 65.53; H, 5.35; N, 8.85; S, 6.59.

5-Hydroxy-2,2-dimethyl-6-(3-phenyl-2,5-dihydro-1,2,4-triazin-5-yl)-10-propyl-2*H*,8*H*-pyrano[2,3-*f*]chromen-8-one **4f**. Yield 186 mg (42%). Beige solid, m.p. 195–197 °C dec. ^1^H NMR (400 MHz, DMSO-d_6_): δ = 7.90–7.92 (m, 2H), 7.47–7.56 (m, 3H), 6.83 (s, 1H), 6.64 (d, *J* = 10.0 Hz, 1H), 5.63 (s, 1H), 5.48 (d, *J* = 10.0 Hz, 1H), 4.85 (s, 1H), 2.80–2.85 (m, 2H), 1.81, 1.59–1.65 (m, 2H), 1.44 (s, 3H), 1.42 (s, 3H), 0.98–1.02 (m, 3H). ^13^C NMR (101 MHz, DMSO-d_6_): δ = 163.3, 160.1, 158.0, 154.6, 153.0, 150.7, 140.6, 131.7, 131.0, 128.6, 127.1, 124.4, 118.1, 107.2, 105.5, 104.4, 98.8, 76.6, 51.3, 38.0, 27.3, 27.2, 23.4, 14.0. Anal. Calcd for C_26_H_25_N_3_O_4_: C, 70.41; H, 5.68; N, 9.47. Found: C, 70.47; H, 5.73; N, 9.28.

5-Hydroxy-2,2-dimethyl-10-propyl-6-(3-(thiophen-2-yl)-2,5-dihydro-1,2,4-triazin-5-yl)-2*H*,8*H*-pyrano[2,3-*f*]chromen-8-one **4g**. Yield 314 mg (70%). Beige solid, m.p. > 220 °C dec. ^1^H NMR (400 MHz, DMSO-d_6_): δ = 13.12 (br s, 1H), 12.02 (br s, 1H), 8.02 (d, *J* = 3.9 Hz, 1H), 7.85 (d, *J* = 5.2 Hz, 1H), 7.25 (dd, *J* = 3.9 Hz, 5.2 Hz, 1H), 7.00 (s, 1H), 6.61 (d, *J* = 10.0 Hz, 1H), 5.97 (s, 1H), 5.69 (d, *J* = 10.0 Hz, 1H), 4.80 (s, 1H), 2.85–2.89 (m, 2H), 1.59–1.63 (m, 2H), 1.58–1.67 (m, 2H), 1.45 (s, 3H), 1.44 (s, 3H), 0.99–1.02 (m, 3H). ^13^C NMR (101 MHz, DMSO-d_6_): δ = 159.2, 157.7, 156.8, 153.0, 150.6, 149.6, 140.4, 134.3, 131.6, 129.2, 128.5, 127.1, 116.0, 109.7, 106.4, 102.1, 100.9, 77.4, 52.8, 37.7, 27.3, 27.2, 23.1, 13.8. Anal. Calcd for C_24_H_23_N_3_O_4_S: C, 64.13; H, 5.16; N, 9.35; S, 7.13. Found: C, 64.14; H, 5.15; N, 7.47; S, 7.21.

##### General Procedure for Aromatization of Dihydrotriazines **4a**–**d** and **4f** to triazines **5a**–**d** and **5f**

To a solution of 5-hydroxy-2,2-dimethyl-6-(2,5-dihydro-1,2,4-triazin-5-yl)-10-propyl-2*H*,8*H*-pyrano[2,3-*f*]chromen-8-ones **4a**–**d**,**f** (1.0 mmol) in 1,2-dichloroethane (5 mL) tetrachloro-1,4-benzoquinone (295 mg, 1.2 mmol) was added. The resulting mixture was stirred under reflux for 5 h. Then, the solvent was removed under reduced pressure and the residue was recrystallized from acetonitrile to give **5a**–**d** and **5f**. Images of ^1^H and ^13^C NMR spectra of compounds **5a**–**d** and **5f** are provided in Supplementary Figures S15–S24.

5-Hydroxy-2,2-dimethyl-6-(3-(methylthio)-1,2,4-triazin-5-yl)-10-propyl-2*H*,8*H*-pyrano[2,3-*f*]chromen-8-one (**5a**). Yield 251 mg (61%). Yellow solid, m.p. 179–181 °C dec. ^1^H NMR (400 MHz, CDCl_3_): δ = 14.13 (s, 1H), 10.23 (s, 1H), 6.76 (d, *J* = 10.0 Hz, 1H), 6.04 (s, 1H), 5.62 (d, *J* = 10.0, 1H), 2.91–2.95 (m, 2H), 2.75 (s, 3H), 1.63–1.73 (m, 2H), 1.55 (s, 6H), 1.04–1.08 (m, 3H). ^13^C NMR (101 MHz, CDCl_3_): δ = 168.9, 161.5, 159.0, 158.6, 156.5, 156.4, 153.3, 145.1, 127.0, 116.1, 111.0, 106.9, 103.7, 97.6, 79.8, 39.1, 28.4, 23.4, 14.12, 14.06. Anal. Calcd for C_21_H_21_N_3_O_4_S: C, 61.30; H, 5.14; N, 10.21; S, 7.79. Found: C, 61.39; H, 5.10; N, 10.00; S, 7.61.

5-Hydroxy-2,2-dimethyl-6-(3-(butylthio)-1,2,4-triazin-5-yl)-10-propyl-2*H*,8*H*-pyrano[2,3-*f*]chromen-8-one (**5b**). Yield 304 mg (67%). Yellow solid, m.p. 136–137 °C. ^1^H NMR (400 MHz, CDCl_3_): δ = 14.14 (s, 1H), 10.18 (s, 1H), 6.73 (d, *J* = 10.0 Hz, 1H), 6.02 (s, 1H), 5.60 (d, *J* = 10.0 Hz, 1H), 3.31–3.35 (m, 2H), 2.85–2.92 (m, 2H), 1.76–1.85 (m, 2H), 1.62–1.71 (m, 2H), 1.48–1.57 (m, 8H), 1.03–1.07 (m, 3H), 0.95–0.99 (m, 3H). ^13^C NMR (101 MHz, CDCl_3_): δ = 168.7, 161.5, 158.9, 158.6, 156.4, 156.3, 153.2, 145.0, 126.9, 116.1, 110.9, 106.9, 103.6, 97.5, 79.8, 39.1, 31.0, 30.8, 28.4, 23.4, 22.1, 14.1, 13.7. Anal. Calcd for C_24_H_27_N_3_O_4_S: C, 63.56; H, 6.00; N, 9.26; S, 7.07. Found: C, 63.68; H, 6.12; N, 9.17; S, 7.15.

6-(3-(But-2-yn-1-ylthio)-1,2,4-triazin-5-yl)-5-hydroxy-2,2-dimethyl-10-propyl-2*H*,8*H*-pyrano[2,3-*f*]chromen-8-one (**5c**). Yield 184 mg (41%). Yellow solid, m.p. > 170 °C dec. ^1^H NMR (400 MHz, CDCl_3_): δ = 13.62 (s, 1H), 10.18 (s, 1H), 6.76 (d, *J* = 10.0 Hz, 1H), 6.04 (s, 1H), 5.62 (d, *J* = 10.0 Hz, 1H), 4.07 (q, *J* = 2.64 Hz, 2H), 2.90–2.94 (m, 2H), 1.83 (t, *J* = 2.64 Hz, 3H), 1.63–1.73 (m, 2H), 1.55 (s, 6H), 1.04–1.08 (m, 3H). ^13^C NMR (101 MHz, CDCl_3_): δ = 167.8, 161.0, 158.9, 158.5, 156.4, 156.3, 153.5, 145.5, 127.0, 116.1, 111.1, 106.9, 103.8, 97.8, 80.3, 79.9, 73.0, 39.1, 28.4, 23.4, 20.4, 14.1, 3.9. Anal. Calcd for C_24_H_23_N_3_O_4_S: C, 64.13; H, 5.16; N, 9.35; S, 7.13. Found: C, 64.14; H, 5.17; N, 9.20; S, 7.19.

6-(3-(Benzylthio)-1,2,4-triazin-5-yl)-5-hydroxy-2,2-dimethyl-10-propyl-2*H*,8*H*-pyrano[2,3-*f*]chromen-8-one (**5d**). Yield 346 mg (71%). Yellow solid, m.p. 158–160 °C dec. ^1^H NMR (400 MHz, CDCl_3_): δ = 13.96 (s, 1H), 10.22 (s, 1H), 7.47–7.49 (m, 2H), 7.32–7.36 (m, 2H), 7.28–7.30 (m, 1H), 6.74 (d, *J* = 10.0 Hz, 1H), 6.04 (s, 1H), 5.60 (d, *J* = 10.0 Hz, 1H), 4.62 (s, 2H), 2.90–2.94 (m, 2H), 1.63–1.72 (m, 2H), 1.54 (s, 6H), 1.04–1.08 (s, 3H). ^13^C NMR (101 MHz, CDCl_3_): δ = 168.0, 161.4, 158.9, 158.6, 156.5, 156.4, 153.4, 145.4, 136.1, 129.4, 128.9, 127.9, 127.0, 116.1, 111.0, 106.9, 103.7, 97.6, 79.8, 39.1, 35.4, 28.4, 23.4, 14.1. Anal. Calcd for C_27_H_25_N_3_O_4_S: C, 66.51; H, 5.17; N, 8.62; S, 6.58. Found: C, 66.53; H, 5.34; N, 8.67; S, 6.57.

6-(3-(Phenyl)-1,2,4-triazin-5-yl)-5-hydroxy-2,2-dimethyl-10-propyl-2*H*,8*H*-pyrano[2,3-*f*]chromen-8-one (**5f**). Yield 212 mg (48%). Yellow solid, m.p. 226–228 °C dec. ^1^H NMR (400 MHz, CDCl_3_): δ = 15.06 (s, 1H), 10.47 (s, 1H), 8.38–8.40 (m, 2H), 7.56–7.62 (m, 3H), 6.79 (d, *J* = 10.1 Hz, 1H), 6.04 (s, 1H), 5.63 (d, *J* = 10.1 Hz, 1H), 2.90–2.94 (m, 2H), 1.64–1.71 (m, 2H), 1.56 (s, 6H), 1.04–1.08 (m, 3H). ^13^C NMR (101 MHz, CDCl_3_): δ = 161.8, 159.4, 159.0, 158.6, 156.5, 156.3, 153.6, 147.3, 133.9, 129.4, 128.9, 128.2, 127.0, 116.1, 110.9, 107.0, 103.7, 97.7, 79.8, 39.1, 28.4, 23.4, 14.1. Anal. Calcd for C_26_H_23_N_3_O_4_: 70.74; H, 5.25; N, 9.52. Found: C, 74.60; H, 5.32; N, 9.63.

#### 2.1.2. Synthesis of 5-(het)Aryl-2,2-dimethyl-10-propyl-2*H*,8*H*-pyrano[2,3-*f*]chromen-8-ones **7a**–**l**

Procedure for the synthesis of 2,2-dimethyl-8-oxo-10-propyl-2*H*,8*H*-pyrano[2,3-*f*]chromen-5-yl nonafluorobutane-1-sulfonate **8**

To the weighed quantity of compound **3** (286 mg, 1.0 mmol) in acetonitrile (30 mL) in a two-necked round bottom flask, K_2_CO_3_ (210 mg, 1.5 mmol) was added and stirred at ambient temperature for 10 min. Perfluorobutanesulfonyl fluoride (362 mg, 1.2 mmol) was then added dropwise, and the reaction mixture was stirred at room temperature for 5 h. After the completion of the reaction monitored by TLC, the reaction mixture was filtered through a celite pad, the filtrate was collected, and the solvent was evaporated under reduced pressure. The resulting solid was crystallized in ethanol to obtain **8** as a white solid with m.p. 113–114 °C (511 mg, 90% yield). ^1^H NMR (400 MHz, DMSO-d_6_): δ = 7.12 (s, 1H), 6.47 (d, *J* = 10 Hz, 1H), 6.29 (s, 1H), 6.06 (d, *J* = 10.4 Hz, 1H), 2.89 (t, *J* = 7.6 Hz, 2H), 1.50–1.63 (m, 2H), 1.50 (s, 6H), 1.00 (t, *J* = 7.2 Hz, 3H). ^13^C NMR (100 MHz, DMSO-d_6_): δ = 158.9, 156.5, 154.7, 152.3, 146.2, 132.5, 115.3, 114.3, 111.2, 110.0, 103.0, 79.5, 37.7, 27.6, 23.0, 14.0. ^19^F NMR (376 MHz, DMSO-d_6_): δ = −80.3, −109.2, −120.6, −125.6.

##### General Procedure for the Suzuki Cross-Coupling Reaction

In a sealed tube with a screw cap, nonaflate **8** (568 mg, 1 mmol), boronic acid (1 mmol), Na_2_CO_3_ (212 mg, 2 mmol), 1,4-dioxane (1 mL), and water (2 mL) were added. The reaction mixture was degassed for 10 min under N_2_ atmosphere, and then Pd(PPh_3_)_2_Cl_2_ (35 mg, 0.05 mmol) was added. The reaction mixture was heated at 90 °C for 4–6 h. After the completion of the reaction, as monitored by TLC, the reaction mixture was filtered through a celite pad, the filtrate was diluted with water (10 mL) and extracted three times with ethyl acetate. The combined organic layers were washed with brine and dried with Na2SO4, and the solvent was removed under reduced pressure to obtain the crude product. The crude product was purified by column chromatography to give the products **7a**–**l**. Images of ^1^H and ^13^C NMR spectra of compounds **8,** and **7a**–**l** are provided in Supplementary Figures S25–S51.

A single crystal of **7g** was grown by the slow evaporation of the EtOAc solution. Single crystal X-ray data for the compound **7g** were collected using the Bruker D8 Quest diffractometer. The crystal was kept at 293.15 K during data collection. Using Olex2 1.3 [24] the structure was solved with the SHELXT [25] structure solution program using Intrinsic Phasing and refined with the SHELXL [26] refinement package using least squares minimization.

2,2-Dimethyl-5-phenyl-10-propyl-2*H*,8*H*-pyrano[2,3-*f*]chromen-8-one (**7a**). Yield 311 mg (90%). Off-white solid; m.p. 122–123 °C. ^1^H NMR (400 MHz, CDCl_3_): δ = 7.38–7.46 (m, 3H), 7.34 (d, *J* = 7.6 Hz, 2H), 6.86 (s, 1H), 6.31 (d, *J* = 10 Hz, 1H), 6.12 (s, 1H), 5.56 (d, *J* = 10 Hz, 1H), 2.97 (t, *J* = 7.6 Hz, 2H), 1.55 (s, 6H), 1.67–1.76 (m, 2H), 1.07 (t, *J* = 7.2 Hz, 3H). ^13^C NMR (100 MHz, CDCl_3_): δ = 160.9, 157.6, 154.5, 151.7, 142.6, 138.4, 129.4, 128.4, 128.1 (2 peaks), 120.8, 115.4, 113.9, 110.4, 108.9, 77.1, 38.6, 27.7, 23.1, 14.0. Anal. Calcd for C_23_H_22_O_3_: C, 79.74; H, 6.40; Found: C, 79.55; H, 6.36.

5-(3-Fluorophenyl)-2,2-dimethyl-10-propyl-2*H*,8*H*-pyrano[2,3-*f*]chromen-8-one (**7b**). Yield 302 mg (83%). Off-white solid; m.p. 132–133 °C. ^1^H NMR (400 MHz, CDCl_3_): δ = 7.39–7.45 (m, 1H), 7.05–7.14 (m, 3H), 6.84 (s, 1H), 6.29 (d, *J* = 10 Hz, 1H), 6.13 (s, 1H), 5.59 (d, *J* = 10 Hz, 1H), 2.97 (t, *J* = 7.6 Hz, 2H), 1.67–1.76 (m, 2H), 1.55 (s, 6H), 1.07 (t, *J* = 7.6 Hz, 3H). ^13^C NMR (100 MHz, CDCl_3_): δ = 163.8 (d, *J* = 246 Hz), 160.7, 157.4, 154.5, 151.7, 141.1 (d, *J* = 1 Hz), 140.6 (d, *J* = 8 Hz), 130.1 (d, *J* = 8 Hz), 128.6, 125.2 (d, *J* = 2 Hz), 120.4, 116.6 (d, *J* = 22 Hz), 115.3, 115.1 (d, J = 22 Hz), 114.1, 110.3, 109.3, 77.2, 38.6, 27.7, 23.1, 14.0. Anal. Calcd for C_23_H_21_FO_3_: C, 75.81; H, 5.81; Found: C, 75.95; H, 5.95.

5-(3-Chlorophenyl)-2,2-dimethyl-10-propyl-2*H*,8*H*-pyrano[2,3-*f*]chromen-8-one (**7c**). Yield 327 mg (86%). Light brown solid; m.p. 125–126 °C. ^1^H NMR (400 MHz, CDCl_3_): δ = 7.38–7.40 (m, 2H), 7.34 (s, 1H), 7.22–7.24 (m, 1H), 6.83 (s, 1H), 6.26 (d, *J* = 10 Hz, 1H), 6.14 (s, 1H), 5.59 (d, *J* = 9.6 Hz, 1H), 2.97 (t, *J* = 7.6 Hz, 2H), 1.67–1.76 (m, 2H), 1.55 (s, 6H), 1.07 (t, *J* = 7.6 Hz, 3H). ^13^C NMR (100 MHz, CDCl_3_): δ = 160.7, 157.4, 154.5, 151.7, 140.9, 140.2, 134.4, 129.8, 129.4, 128.6, 128.2, 127.6, 120.3, 115.3, 114.2, 110.3, 109.3, 77.2, 38.6, 27.7, 23.1, 14.0. Anal. Calcd for C_23_H_21_ClO_3_: C, 72.53; H, 5.56; Found: C, 72.67; H, 5.83.

5-(3-Methoxyphenyl)-2,2-dimethyl-10-propyl-2*H*,8*H*-pyrano[2,3-*f*]chromen-8-one (**7d)**. Yield 346 mg (92%). Cream solid; m.p. 118–119 °C. ^1^H NMR (400 MHz, CDCl_3_): δ = 7.36 (t, *J* = 8 Hz, 1H), 6.91–6.96 (m, 2H), 6.87–6.88 (m, 2H),6.34 (d, *J* = 10 Hz, 1H), 6.12 (s, 1H), 5.56 (d, *J* = 10 Hz, 1H), 3.85 (s, 3H), 2.97 (t, *J* = 7.6 Hz, 2H), 1.67–1.76 (m, 2H), 1.54 (s, 6H), 1.07 (t, *J* = 7.2 Hz, 3H). ^13^C NMR (100 MHz, CDCl_3_): δ = 160.9, 159.5, 157.6, 154.4, 151.6, 142.4, 139.8, 129.5, 128.1, 121.9, 120.8, 115.4, 115.2, 113.9, 113.4, 110.3, 109.0, 77.1, 55.4, 38.6, 27.7, 23.1, 14.0. Anal. Calcd for C_24_H_24_O_4_: C, 76.57; H, 6.43; Found: C, 76.32; H, 6.81.

5-(4-Methoxyphenyl)-2,2-dimethyl-10-propyl-2*H*,8*H*-pyrano[2,3-*f*]chromen-8-one (**7e**). Yield 338 mg (90%). White solid; m.p. 122–123 °C. ^1^H NMR (600 MHz, CDCl_3_): δ = 7.28 (d, *J* = 8.4 Hz, 2H), 6.98 (d, *J* = 8.4 Hz, 2H), 6.84 (s, 1H), 6.34 (d, *J* = 10.2 Hz, 1H), 6.10 (s, 1H), 5.56 (d, *J* = 10.2 Hz, 1H), 3.86 (s, 3H), 2.96 (t, *J* = 7.8 Hz, 2H), 1.67–1.74 (m, 2H), 1.54 (s, 6H), 1.06 (t, *J* = 7.2 Hz, 3H). ^13^C NMR (150 MHz, CDCl_3_): δ = 161.0, 159.6, 157.7, 154.5, 151.7, 142.4, 130.7 (2 peaks), 127.9, 121.0, 115.4, 113.9, 113.6, 110.2, 108.6, 76.9, 55.4, 38.6, 27.6, 23.2, 14.0. Anal. Calcd for C_24_H_24_O_4_: C, 76.57; H, 6.43; Found: C, 76.41; H, 6.55.

2,2-Dimethyl-10-propyl-5-(3,4,5-trimethoxyphenyl)-2*H*,8*H*-pyrano[2,3-*f*]chromen-8(2*H*)-one (**7f**). Yield: 392 mg (90%); white solid; m.p. 167–168 °C. ^1^H NMR (600 MHz, CDCl_3_): δ = 6.86 (s, 1H), 6.53 (s, 2H), 6.38 (d, *J* = 10.2 Hz, 1H), 6.11 (s, 1H), 5.57 (d, *J* = 9.6 Hz, 1H), 3.91 (s, 3H), 3.88 (s, 3H), 2.97 (t, *J* = 7.8 Hz, 2H), 1.68–1.74 (m, 2H), 1.55 (s, 6H), 1.06 (t, *J* = 7.2 Hz, 3H). ^13^C NMR (150 MHz, CDCl_3_): δ = 160.9, 151.6, 142.6, 138.0, 157.6, 154.4, 153.1, 134.0, 128.2, 120.6, 115.3, 113.9, 110.2, 108.9, 106.7, 77.1, 61.0, 56.3, 38.6, 27.8, 23.1, 14.0. Anal. Calcd for C_26_H_28_O_6_: C, 71.54; H, 6.47; Found: C, 71.35; H, 6.09.

2,2-Dimethyl-10-propyl-5-(3-(trifluoromethoxy)phenyl)-2*H*,8*H*-pyrano[2,3-*f*]chromen-8-one (**7g**). Yield 361 mg (84%). Off-white solid; m.p. 143–144 °C. ^1^H NMR (600 MHz, CDCl_3_): δ = 7.48 (t, *J* = 7.8 Hz, 1H), 7.26–7.30 (m, 2H), 7.20 (s, 1H), 6.84 (s, 1H), 6.26 (d, *J* = 10.2 Hz, 1H), 6.14 (s, 1H), 5.60 (d, *J* = 10.2 Hz, 1H), 2.97 (t, *J* = 7.8 Hz, 2H), 1.68–1.74 (m, 2H), 1.55 (s, 6H), 1.07 (t, *J* = 7.2 Hz, 3H). ^13^C NMR (150 MHz, CDCl_3_): δ = 160.7, 157.4, 154.5, 151.8, 149.2, 140.7, 140.4, 129.9, 128.8, 127.8, 122.0, 120.5 (q, *J* = 257 Hz), 120.4, 120.2, 115.3, 114.2, 110.3, 109.4, 77.2, 38.6, 27.7, 23.1, 14.0. Anal. Calcd for C_24_H_21_F_3_O_4_: C, 66.97; H, 4.92; Found: C, 67.10; H, 4.74.

5-(4-Aminophenyl)-2,2-dimethyl-10-propyl-2*H*,8*H*-pyrano[2,3-*f*]chromen-8-one (**7h**). Yield 278 mg (77%). Beige solid; m.p. 147–149 °C. ^1^H NMR (400 MHz, CDCl_3_): 7.16 (d, *J* = 8 Hz, 2H), 6.84 (s, 1H), 6.74 (d, *J* = 8.4 Hz, 2H), 6.39 (d, *J* = 10 Hz, 1H), 6.08 (s, 1H), 5.55 (d, *J* = 9.6 Hz, 1H), 3.83 (s, 2H), 2.96 (t, *J* = 7.6 Hz, 2H), 1.66–1.75 (m, 2H), 1.54 (s, 6H), 1.06 (t, *J* = 7.2 Hz, 3H). ^13^C NMR (100 MHz, CDCl_3_): δ = 161.1, 157.8, 154.6, 151.7, 146.6, 143.0, 130.6, 128.3, 127.6, 121.3, 115.3, 114.8, 113.4, 110.0, 108.3, 76.8, 38.6, 27.6, 23.2, 14.0. Anal. Calcd for C_23_H_23_NO_3_: C, 76.43; H, 6.41; N, 3.88; Found: C, 76.25; H, 6.17; N, 4.07.

2,2-Dimethyl-5-(naphthalen-2-yl)-10-propyl-2*H*,8*H*-pyrano[2,3-*f*]chromen-8-one (**7i**). Yield 360 mg (91%). Cream solid; m.p. 123–125 °C. ^1^H NMR (400 MHz, CDCl_3_): δ = 7.89–7.93 (m, 3H), 7.82 (s, 1H), 7.53–7.56 (m, 2H), 7.47 (dd, *J* = 8.4 Hz, 1.6 Hz, 1H), 6.97 (s, 1H), 6.35 (d, *J* = 10 Hz, 1H), 6.14 (s, 1H), 5.57 (d, *J* = 10 Hz, 1H), 2.99 (t, *J* = 7.6 Hz, 2H), 1.69–1.77 (m, 2H), 1.58 (s, 6H), 1.08 (t, *J* = 7.2 Hz, 3H). ^13^C NMR (100 MHz, CDCl_3_): δ = 161.0, 157.6, 154.5, 151.7, 142.6, 128.6, 128.2 (2 peaks), 128.1, 127.7, 127.3, 126.6 (2 peaks), 120.8, 115.6, 113.9, 110.7, 109.0, 77.1, 38.6, 27.7, 23.2, 14.0. Anal. Calcd for C_27_H_24_O_3_: C, 81.79; H, 6.10; Found: C, 82.14; H, 5.95.

5-(Furan-3-yl)-2,2-dimethyl-10-propyl-2*H*,8*H*-pyrano[2,3-*f*]chromen-8-one (**7j**). Yield 286 mg (85%). White solid; m.p. 129–131 °C. ^1^H NMR (600 MHz, CDCl_3_): δ = 7.56 (s, 1H), 7.51 (t, *J* = 1.8 Hz, 1H), 6.87 (s, 1H), 6.57 (d, *J* = 9.6 Hz, 1H), 6.55–6.56 (m, 1H), 6.09 (s, 1H), 5.62 (d, *J* = 10.2 Hz, 1H), 2.94 (t, *J* = 7.8 Hz, 2H), 1.66–1.72 (m, 2H), 1.53 (s, 6H), 1.05 (t, *J* = 7.2 Hz, 3H). ^13^C NMR (150 MHz, CDCl_3_): δ = 160.8, 157.5, 154.6, 151.7, 143.4, 141.1, 133.2, 128.5, 123.3, 120.5, 115.4, 113.8, 111.4, 109.7, 108.9, 77.0, 38.6, 27.6, 23.1, 14.0. Anal. Calcd for C_21_H_20_O_4_: C, 74.98; H, 5.99; Found: C, 75.35; H, 5.90.

5-(5-Acetylthiophen-2-yl)-2,2-dimethyl-10-propyl-2*H*,8*H*-pyrano[2,3-*f*]chromen-8-one (**7k**). Yield 323 mg (82%). Off-white solid; m.p. 147–148 °C. ^1^H NMR (600 MHz, CDCl_3_): δ = 7.68 (d, *J* = 3.6 Hz, 1H), 7.13 (d, *J* = 3.6 Hz, 1H), 6.95 (s, 1H), 6.57 (d, *J* = 10.2 Hz, 1H), 6.13 (s, 1H), 5.66 (d, *J* = 10.2 Hz, 1H), 2.94 (t, *J* = 7.8 Hz, 2H), 2.58 (s, 3H), 1.65–1.72 (m, 2H), 1.54 (s, 6H), 1.05 (t, *J* = 7.2 Hz, 3H). ^13^C NMR (150 MHz, CDCl_3_): δ = 190.6, 160.4, 157.2, 154.4, 152.0, 148.1, 145.3, 133.5, 132.6, 129.3, 129.0, 120.1, 115.6, 114.6, 110.6, 110.1, 77.3, 38.5, 27.6, 26.8, 23.1, 14.0. Anal. Calcd for C_23_H_22_O_4_S: C, 70.03; H, 5.62; S, 8.13; Found: C, 69.99; H, 5.49; S, 8.28.

2,2-Dimethyl-10-propyl-5-(pyridin-3-yl)-2*H*,8*H*-pyrano[2,3-*f*]chromen-8-one (**7l**). Yield 298 mg (86%). Beige solid; m.p. 135–137 °C. ^1^H NMR (400 MHz, CDCl_3_): δ = 8.65 (d, *J* = 4.4 Hz, 1H), 8.61 (s, 1H), 7.68 (d, *J* = 7.6 Hz, 1H), 7.38–7.41 (m, 1H), 6.83 (s, 1H), 6.23 (d, *J* = 10 Hz, 1H), 6.13 (s, 1H), 5.60 (d, *J* = 9.6 Hz, 1H), 2.96 (t, *J* = 7.6 Hz, 2H), 1.66–1.75 (m, 2H), 1.55 (s, 6H), 1.06 (t, *J* = 7.2 Hz, 3H). ^13^C NMR (100 MHz, CDCl_3_): δ = 160.6, 157.3, 154.6, 151.9, 150.0, 149.3, 138.5, 136.6, 134.2, 129.0, 123.3, 120.0, 115.5, 114.3, 110.4, 109.6, 77.3, 38.4, 27.6, 23.1, 14.0. Anal. Calcd for C_22_H_21_NO_3_: C, 76.06; H, 6.09; N, 4.03; Found: C, 76.08; H, 6.09; N, 3.90.

#### 2.1.3. Synthesis of Aminomethyl Derivatives of 5-Hydroxy-2,2-dimethyl-10-propyl-2*H*,8*H*-pyrano[2,3-*f*]chromen-8-one **9a**–**c**

##### General Procedure for the Mannich Reaction

Pyranocoumarin **3** (286 mg, 1.0 equiv.) was dissolved in dioxane (15 mL), formaldehyde (82 mg, 1.1 equiv.) and the corresponding amine (1.1 equiv.) were added to the solution. The reaction mixture was heated under reflux for 6 h, was poured into water, and the solid was filtered, dried and crystallized from aqueous ethanol (1:1). Images of ^1^H and ^13^C NMR spectra of compounds **9a**–**c** are provided in Supplementary Figures S52–S57.

5-Hydroxy-2,2-dimethyl-6-(piperidin-1-ylmethyl)-10-propyl-2*H*,8*H*-pyrano[2,3-*f*]chromen-8-one **9a**. Yield 260 mg (71%). Off-white solid; m.p. 146–148 °C. ^1^H NMR (400 MHz, DMSO-d_6_): δ = 6.57 (d, *J* = 9.9 Hz, 1H), 5.83 (s, 1H), 5.61 (d, *J* = 9.9 Hz, 1H), 5.24 (br s, 1H), 3.90 (s, 2H), 2.80–2.84 (m, 2H), 2.63 (br s, 4H), 1.57–1.58 (m, 6H), 1.34–1.46 (m, 8H), 0.96–1.00 (m, 3H). ^13^C NMR (101 MHz, DMSO-d_6_): δ = 160.0, 159.5, 158.1, 153.0, 150.4, 126.3, 116.4, 108.2, 105.8, 100.6, 99.5, 77.2, 53.9, 52.7, 37.8, 27.3, 25.0, 23.2, 22.9, 13.8. Anal. Calcd for C_23_H_29_NO_4_: C, 72.04; H, 7.62; N, 3.65. Found: C, 71.85; H, 7.67; N, 3.72.

5-Hydroxy-2,2-dimethyl-6-(morpholinomethyl)-10-propyl-2*H*,8*H*-pyrano[2,3-*f*]chromen-8-one **9b**. Yield 250 mg (65%). Off-white solid; m.p. 178–180 °C. ^1^H NMR (400 MHz, CDCl_3_): δ = 9.65 (br s, 1H), 6.65 (d, *J* = 9.9 Hz, 1H), 5.89 (s, 1H), 5.53 (d, *J* = 9.9 Hz, 1H), 3.97 (s, 2H), 3.76 (br s, 4H), 2.86–2.90 (m, 2H), 2.65 (br s, 4H), 1.62–1.68 (m, 2H), 1.48 (s, 6H), 1.01–1.04 (m, 3H). ^13^C NMR (101 MHz, CDCl_3_): δ = 161.1, 158.9, 157.7, 153.5, 151.4, 126.8, 116.7, 109.9, 106.4, 103.0, 99.9, 77.8, 66.8, 54.5, 53.0, 38.8, 28.0, 23.4, 14.1. Anal. Calcd for C_22_H_27_NO_5_: C, 68.55; H, 7.06; N, 3.63. Found: C, 68.66; H, 7.16; N, 3.77.

5-Hydroxy-2,2-dimethyl-10-propyl-6-(pyrrolidin-1-ylmethyl)-2*H*,8*H*-pyrano[2,3-*f*]chromen-8-one **9c**. Yield 250 mg (69%). Off-white solid; m.p. 115–117 °C. ^1^H NMR (400 MHz, DMSO-d_6_): δ = 6.59 (d, *J* = 9.9 Hz, 1H), 5.76 (s, 1H), 5.56 (d, *J* = 9.9 Hz, 1H), 4.07 (s, 2H), 2.79–2.83 (m, 6H), 1.84 (br s, 4H), 1.55–1.61 (m, 2H), 1.42 (s, 6H), 0.96–1.00 (m, 3H). ^13^C NMR (101 MHz, DMSO-d_6_): δ = 161.8, 159.7, 158.2, 153.1, 150.7, 125.6, 116.9, 107.2, 106.1, 100.1, 99.6, 77.0, 52.7, 50.9, 37.8, 27.3, 23.2, 23.2, 13.9. Anal. Calcd for C_22_H_27_NO_4_: C, 71.52; H, 7.37; N, 3.79. Found: C, 71.59; H, 7.52; N, 3.85.

### 2.2. Anti-HIV Assay

#### 2.2.1. Antiviral Activity on the Model HIV-1 Reverse Transcriptase

The activity of RT inhibition by the tested compounds was measured using colorimetric enzyme immunoassay in accordance with the manufacturer’s procedure [27]. The compounds were first diluted in 2% propylene glycol and then in a lysis buffer to the tested concentration of 50 µM. Nevirapine (abcr, Karlsruhe, Germany) was used as a positive control. At the end of the assay, the optical density was measured at 405 nm using a microplate reader (Victor Nivo™, PerkinElmer, Shelton, CT, USA).

Statistical data processing was performed in the RStudio program (version 2023.03.0 build 386 © 2009–2023 Posit Software, PBC) using the R packages (version 4.3.2). The data is presented as mean ± standard deviation (SD). Differences between the groups were determined using a *t*-test adjusted for Holm’s multiplicity.

#### 2.2.2. Antiviral Activity in a Model of Human Cells Infected with HIV-1

- Cells.

Transfected human MT-4 lymphoblastoid cells were used. Cells were cultured in RPMI 1640 medium with 10% bovine embryo serum, 100 μg/mL penicillin and streptomycin.

- Viruses.

HIV-1_899A_ strain, from the collection of human immunodeficiency virus strains of the Institute of Virology named after D.I. Ivanovsky, was used as a virus source.

- Assay Design.

Evaluation of the cytotoxic effect.

The tested compounds were added to the cells at the same concentrations to determine antiviral activity (0.25 to 100 μM). Cells were incubated at 37 °C, 5% CO_2_, and 98% humidity for 4–6 days. Cell viability was assessed by MTT assay.

Evaluation of the antiviral activity of the compounds

The tested compounds were added to the cells at various doses while simultaneously infected with the virus at a dose of 0.01 TCID_50_/cell. Cell cultures were incubated at 37 °C, 5% CO_2_, and 98% humidity for 6 days. The results were evaluated using MTT assay with spectrophotometry and light microscopy: investigation of the cytopathic effect of the virus (CPE) and virus-induced syncytium formation.

### 2.3. Computational Study

- Ligand preparation.

All ligands were prepared using DataWarrior 5.5.0 software [28] with conformers generation (1 per ligand) on MMFF94s+ force field and self-assembly algorithm.

- Docking protocol

The protein-ligand complex (complex of RT and NNRTI CHEMBL4094163 (for structure of CHEMBL4094163 see Supplementary Figure S58), PDB ID: 6C0N) was exported from the RCSB databank in .pdb format. Water and other small molecules (except native NNRTI) were manually removed. The prepared complex was downloaded into Jamda tool.

The binding site was determined from the native ligand (NNRTI) position. Docking was performed using default Jamda parameters excluding site radius (changed from default 6.5 Å to 8 Å).

- Validation.

First, native NNRTI was redocked from the pdb:6c0n complex to assess the quality (reproducibility of the native ligand position) of the method and docking parameters used. As a result, the calculated root-mean-square deviation (RMSD) and Jamda score values for the native ligand were 0.52 Å [29] and −4.8, respectively.

Additional validation was performed by docking known NNRTIs to assess the correlation between the calculated Jamda score and in vitro inhibitory activity. NNRTIs for validation with a wide range of IC_50_ values were downloaded from the ChEMBL database using DataWarrior 5.5.0 software.

Ligand preparation and docking procedures were performed as described above.

Correlation between in vitro activity (e.g., IC_50_ values) and Jamda score was assessed using Pearson linear correlation and Spearman’s rank test. For the known NNRTIs, a moderately good correlation was obtained by both Pearson (0.66) and Spearman (0.56) tests (Supplementary Table S1) [30,31,32,33,34,35,36].

After validation, studied compounds were docked using the same docking parameters. In addition, two-dimensional maps of non-covalent interactions between docked ligands and binding site residues were generated using the PoseEdit tool [37] from the ProteinsPlus web server. The superposition of all docked ligands and native NNRTI in the binding site was represented using VMD 1.9.3 software [38].

## 3. Results and Discussion

### 3.1. Chemistry

**Scheme 1:**
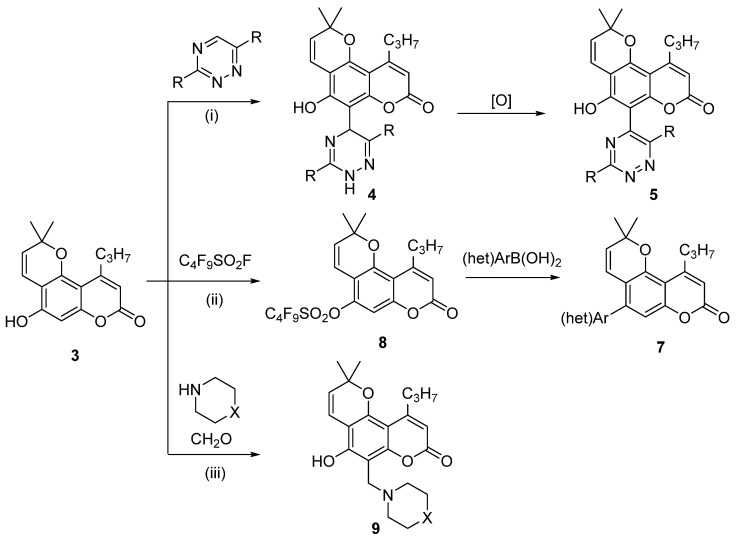
General outline of the synthetic routes studied.

This report presents the synthesis of pyranocoumarin derivatives comprising the moieties of nitrogen-containing heterocycles (Scheme 1). Three directions of the modification were investigated: (i) reaction with 1,2,4-triazines, (ii) Suzuki cross-coupling reaction between pyranocoumarin O-perfluorobutane sulfonyl derivative and boronic acids, and (iii) Mannich aminomethylation.

#### 3.1.1. Synthesis of Calanolide Analogues with 1,2,4-Triazine Moiety

**Scheme 2:**
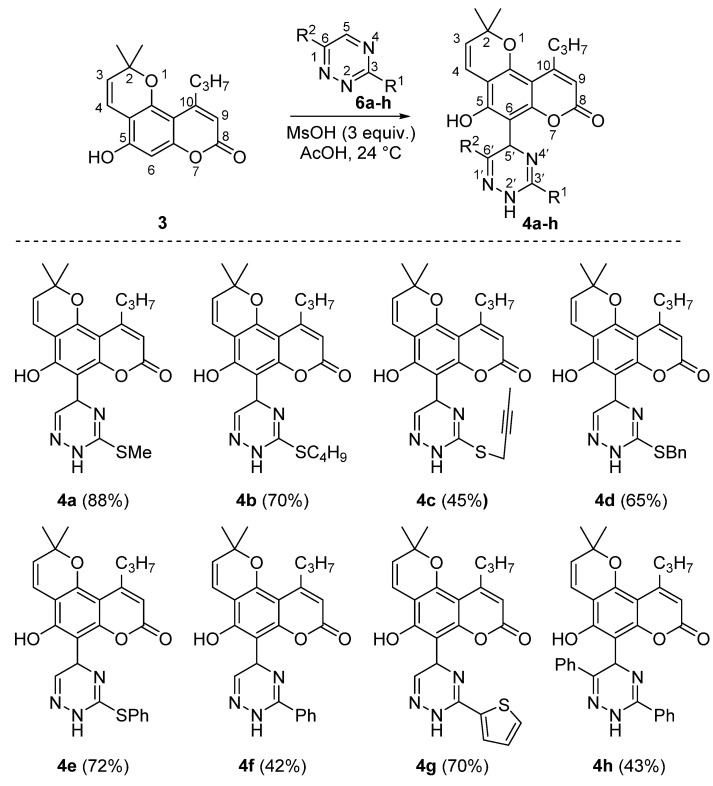
Reaction of compound **3** with 1,2,4-triazines **6a**–**h**.

We have adapted the previously published procedure for the reaction of 5,7-dihydroxycoumarin derivatives [39,40], the parent structure for annulated coumarin **3**, with 1,2,4-triazines under acid catalyzed conditions. The reaction of pyranocoumarin **3** with triazines **6a**–**h** proceeded smoothly in an acetic acid solution in the presence of methanesulfonic acid yielding dihydrotriazines **4a**–**h** (Scheme 2). The reaction proceeds between the most electrophilic position 5 of the triazine and the accessible nucleophilic position 6 of the pyranocoumarin **3** giving the desired products in 42 to 88% yield. The structure of the obtained products is in agreement with the NMR spectroscopic data. Thus, a singlet at 4.9 ppm of hydrogen at C5′ is registered in the ^1^H NMR spectrum, while a signal of carbon atom C6 at 55 ppm appears in the ^13^C NMR spectrum.

**Scheme 3:**
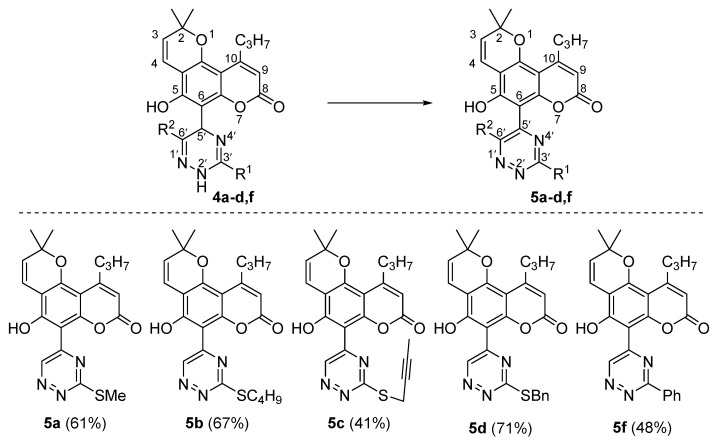
Aromatization of dihydrotriazines **4a**–**d**,**f** to triazines.

Dihydrotriazines **4** were oxidized with chloranil in dichloroethane solution to obtain aromatic triazines **5a**–**d**,**f** in 41–71% yields (Scheme 3). The aromatization of the dihydrotriazine ring is supported by NMR data: the signal of the proton at the C5′ carbon atom at 5.7 ppm disappears in the ^1^H NMR spectra, and, in the ^13^C NMR spectrum, the signal of the C5′ carbon atom at 55 ppm is shifted to downfield.

#### 3.1.2. Synthesis of Calanolide Analogues with (het)Aryl Moiety via Suzuki Cross-Coupling Reaction

**Scheme 4:**
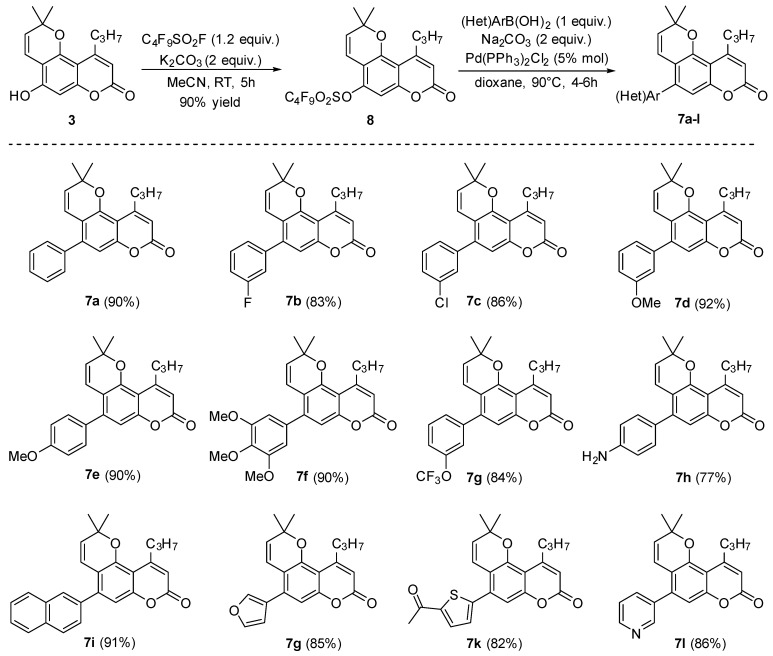
Synthesis of compounds **7a**–**l** using Suzuki cross-coupling reaction.

The second part of this work aims to synthesize calanolide analogues in which ring C is substituted with aromatic or heteroaromatic groups. For the introduction of the (het)aryl group, we used the C-X/C-Y cross-coupling reaction, a versatile tool for creating a new C-C bond. In the first step, the 5-hydroxy group was activated by conversion to a perfluorobutanesulfonyloxy group, followed by substitution of the perfluorobutanesulfonyloxy group under the action of arylboronic acids in the presence of a palladium catalyst. Thus, compound **3** was converted into a sulfonyloxy derivative **8** under the action of perfluorobutanesulfonyloxy fluoride in the presence of K_2_CO_3_, in 90% yield (Scheme 4). Perfluorobutanesulfonyl derivate **8** was then reacted with boronic acids in the presence of bis(triphenylphosphine)palladium chloride to give the desired derivatives **7a**–**l** in high yields (Scheme 4).

The structures of compounds **7** and **8** are in good agreement with their spectral data. Thus, in the ^19^F NMR spectrum of compound **8**, signals with δ = −125.6, −120.6, −109.2, and −80.3 ppm are registered, which is characteristic for the perfluorobutylsulfonyl group, and in the ^1^H NMR spectra of compounds **7a**–**l** a signal of the aromatic group in the region of 7–8 ppm is observed.

**Figure 2:**
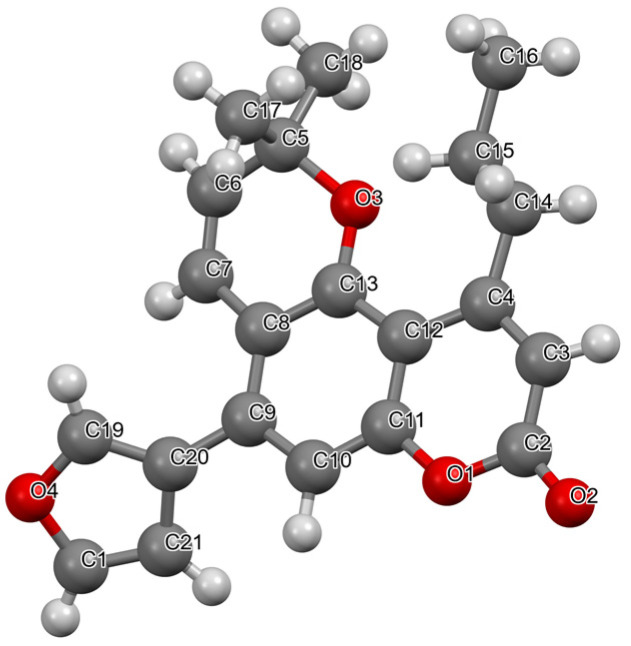
X-ray diffraction data for compound **7g** (CCDC 2309635).

In order to further verify the structure of one of the products **7**, we performed an X-ray diffraction analysis of one of the compounds, **7g**. The data shown in Figure 2 fully supports the proposed structure of this compound.

#### 3.1.3. Synthesis of Calanolide Analogues with Aminomethyl Moiety

**Scheme 5:**
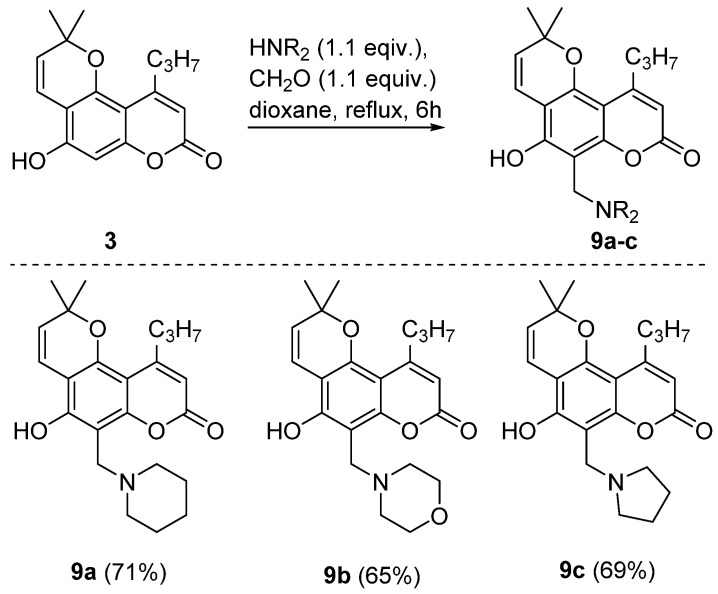
Mannich aminomethylation of compound **3** with cyclic secondary amines.

The Mannich reaction is a convenient tool for the one-step modification of nucleophilic compounds, such as phenols, using formaldehyde and a primary or secondary amine. In the present work, we have used the Mannich reaction for the aminomethylation of 5-hydroxy pyranocoumarin **3** using secondary amines. The desired products **9a**, **9b**, and **9c** were obtained when compound **3** was refluxed with formaldehyde and amines such as piperidine, morpholine, and pyrrolidine (Scheme 5). The structure of the obtained compounds **9** is supported by the ^1^H NMR spectrum: a singlet of methylene group linking pyranocoumarin with amine is registered at 4 ppm, and multiplets of methylene groups of the cyclic amine moieties are observed at 2.0–1.0 ppm.

### 3.2. Anti-HIV Assay

#### 3.2.1. Antiviral Activity on the Model HIV-1 Reverse Transcriptase

**Figure 3:**
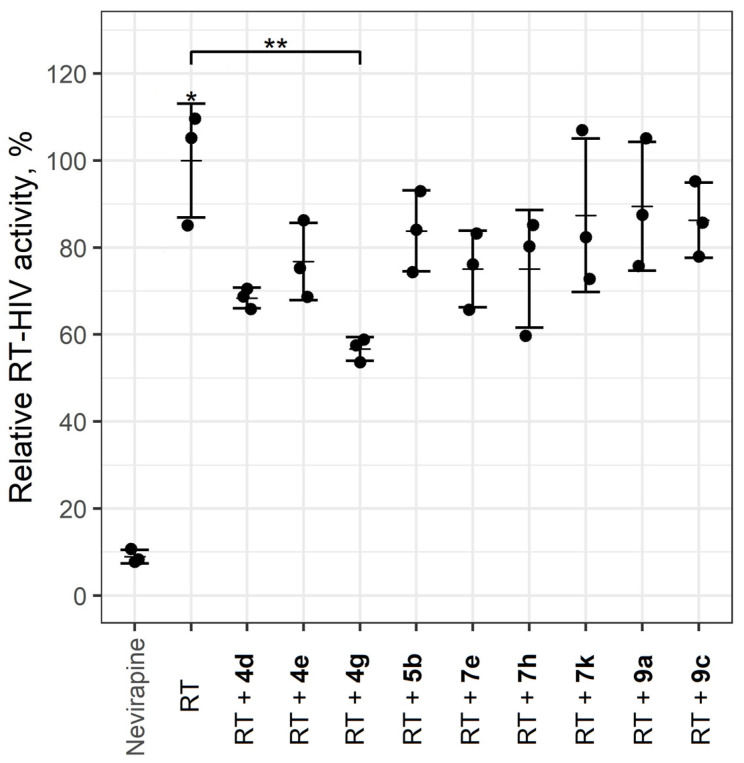
Effect of pyrano[2,3-*f*]chromen-8-ones on HIV reverse transcriptase activity (mean ± SD, n = 3). * *p* < 0.05, ** *p* < 0.01.

We describe the results of the anti-HIV assay showing a decrease in HIV-1 RT activity under the influence of all pyrano[2,3-*f*]chromen-8-ones (Figure 3). As shown in Figure 3, dihydrotriazines **4** demonstrate the highest activity among the studied compounds. They are characterized by RT inhibition from 43% to 22% at a concentration of 50 μM. Compounds **7** are less active, inhibiting RT from 17 to 22% at the same concentration. Compounds **5** and **9** are the least active among those tested, characterized by an inhibition of RT of slightly more than 10% at 50 μM. Compound **4g** is the most active of the series studies, inhibiting RT by 43% at 50 μM (*p* < 0.01). Although this compound has not reached the level of Nevirapine, we consider that optimization of substituents in these dihydrotriazines can further enhance the activity of this type of compound.

Since compound **4** showed the best activity in the RT assay, we focused further cell assays on compounds of this type.

#### 3.2.2. Antiviral Activity in the Model of Human Cells Infected with HIV-1

Compounds **4a**,**e**,**h** and **7l** were tested in three experiments, with three replicates of seven concentrations: 100 μM; 75 μM; 50 μM; 25 μM; 10 μM; 1.0 μM; 0.5 μM.

When added to a medium in wells, all compounds showed some precipitation at any ratio of DMSO to medium (1–4%). This was particularly evident at the highest concentrations (25.0–100.0 μM). The compounds do not induce toxicity at concentrations of 10 μM and less; therefore, anti-HIV-1 activity can be evaluated at these concentrations. The obtained results are provided in Table 1.

Compounds showed pronounced cytotoxicity at concentrations of 25.0–100.0 μM, and all compounds in the range of 1–4% DMSO content in the growth medium showed some precipitate at these concentrations, which also could affect cell growth and viability. Experiments were performed under the condition of 1–2% DMSO content in the medium to avoid the negative effect on the cell viability at the studied concentrations.

### 3.3. Computational Study

To determine the possible binding modes of the tested compounds to HIV-1 reverse transcriptase, molecular docking based on a complex (PDB: 6C0N) of the protein with NNRTI CHEMBL4094163 (see structure in Supplementary Figure S58) with nanomolar activity (IC_50_ = 4.3 nM) [41] was performed using the Jamda tool [42,43,44,45] on the ProteinsPlus web service [46,47]. Additionally, using the SwissADME service [48], some pharmacokinetic characteristics of ligands were calculated, including topological polar surface area (TPSA), lipophility (WlogP), solubility (ESOL logS), gastrointestinal absorption (GI) and blood-brain-barrier permeability [49] and possible inhibition of some cytochrome P450 enzymes.

The results of the computational studies are presented in Table 2.

It is worth noting the relatively high Pearson and Spearman correlation values between the in vitro activity value and the in silico Jamda score.

**Figure 4:**
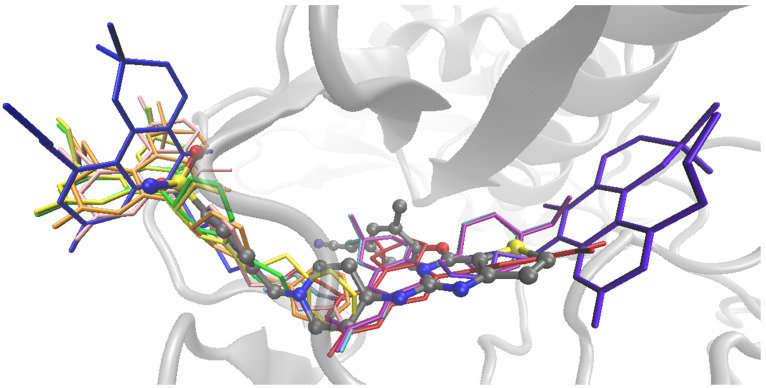
Superposition of all ligands relative to native NNRTI (CPK colors): **4d** (yellow), **4e** (orange), **4g** (green), **5b** (pink), **7e** (purple), **7h** (cyan), **7k** (red), **9a** (blue), **9c** (purple).

For most compounds (except for compounds **7e**,**h**,**k**) a significant difference in their calculated top-1 positions near the NNBS (nonnucleoside binding site) relative to the native ligand position was found. The top-1 positions of all studied ligands relative to the native ligand are presented below (Figure 4).

**Figure 5:**
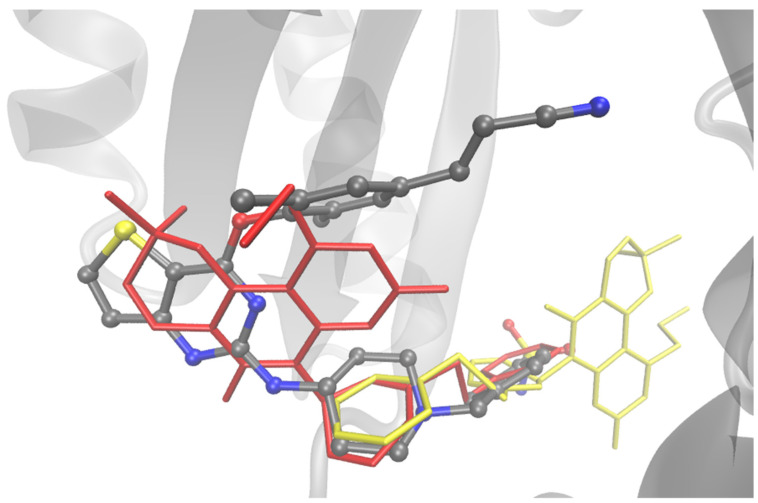
Calculated top-1 (yellow, Jamda score = −2.31) and top-2 (red, Jamda score = −2.14) positions of ligand **4d** relative to the native ligand (CPK colors).

For example, one of the hits 3 interacts with NNBS with only a fragment of its structure. For this ligand, positions within NNBS were also found to have good overlap with the native ligand but with a slight decrease in Jamda score (Figure 5).

**Figure 6:**
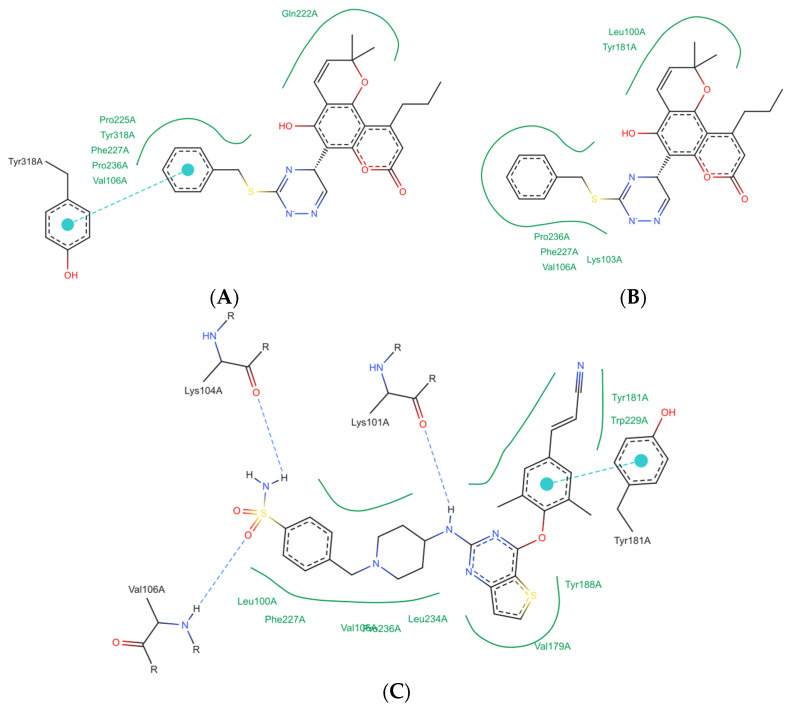
Non-covalent interaction maps of ligands: (**A**)—ligand **4d** in top-1 position; (**B**)—ligand **4d** in top-2 position; (**C**) native ligand.

Despite the ways in which the compounds are oriented relative to the native ligand, their non-covalent interaction profiles overlap only due to hydrophobic interactions. For example, the top-2 position of ligand 3, despite overlapping with the native ligand backbone (Figure 5), is characterized only by hydrophobic contacts (Figure 6B), whereas the top-1 position binds due to π-π stacking interactions (Figure 6A). The native NNRTI, in this case, forms a series of hydrogen bonds with residues Lys101, Lys104, Val106, stacking interactions with residue Tyr181 and a set of hydrophobic interactions (Figure 6C). Non-covalent interaction maps of the ligands at the top-1 positions are presented in Supplementary Figure S59.

On the one hand, the docking results may indicate both the found alternative way of binding calanolide A analogs to NNBS and the necessity for further work of simulation by molecular dynamics method for additional consideration of protein conformation changes during interaction with the studied ligands inside NNBS.

## 4. Conclusions

Herein, we reported the synthesis and anti-HIV-1 evaluation of novel calanolide analogs containing nitrogen heterocycles or aromatic groups instead of ring C. The key intermediate in this synthesis is 5-hydroxy-2,2-dimethyl-10-propyl-2*H*,8*H*-pyrano[2,3-*f*]chromen-8-one **3**. Three routes for the synthesis of the calanolide analogues were used.

- Reaction of compound **3** with 1,2,4-triazines in the presence of methanesulfonic acid afforded dihydrotriazines **4**. The dihydrotriazine ring was aromatized by refluxing with tetrachloro-1,4-benzoquinone to obtain compound **5**.
- Sulfonylation of compound **3** followed by Suzuki cross-coupling reaction with (het)aryl boronic acids.
- Aminomethylation of **3** by Mannich reaction with cyclic secondary amines.

Antiviral assay of the synthesized compounds showed moderate activity of compound **4** against HIV-1 on the enzyme model and poor anti-HIV-1 activity on the cell model. Other compounds have low activity on both enzyme and cell models.

A good correlation was observed between HIV-1 reverse transcriptase docking data and in vitro activity values. Alternative binding modes of Calanolide A analogues to NNBS were also found.

## Figures and Tables

**Table 1:** Development of cytopathic effect (%) in MT-4 cells infected with HIV-1 strain with different doses of the tested compounds on the 4th–6th day of observation.

Compound	Concentration ^1^								
	100 μM	75 μM	50 μM	25 μM	5–10 μM	1 μM	0.5 μM	0.1 μM	0.01 μM
**4a**	T	T	T	25% (T)	42.5–95%	45–95%	70–98%		
**4e**	T	T	T	T	62.5–95%	62.5–95%	70–98%		
**4h**	T	95%	95%	95%	42.5–95%	45–98%	100%		
**7l ** ^2^	T	T	T	0% (T)	25.0–80%	62.5–80%	70–98%		
Zidovudine						0%	0%	0%	37.5–50%

^1^ “T” indicates toxicity due to, among other things, precipitation of the drug. ^2^ Compound **7l** initially (4th day of observation) showed more restrained dynamics of cytopathic effect development at concentrations of 5.0–10.0 μM.

**Table 2:** Docking results of the investigated ligands, in vitro-in silico correlation and calculated ADME characteristics.

Ligand	Inhibition at 50 µM, %	Jamda Score	SwissADME Characteristics						
			TPSA	WlogP	ESOL logS	GI Absorption	BBB Permeant	CYP1A2 Inhibitor	CYP3A4 Inhibitor
**4g**	43	−2.47	124.66	3.2	−5.24	High	No	Yes	Yes
**4d**	32	−2.31	121.72	3.83	−5.82	High	No	Yes	Yes
**7h**	25	−2.20	65.46	5.08	−5.26	High	Yes	Yes	Yes
**7e**	25	−1.93	48.67	5.5	−5.68	High	Yes	Yes	No
**4e**	23	−2.05	121.72	3.84	−5.85	High	No	Yes	Yes
**5b**	16	−2.25	123.64	5.27	−5.33	Low	No	No	Yes
**9c**	14	−1.88	62.91	3.59	−4.47	High	Yes	Yes	No
**7k**	13	−1.97	84.75	5.75	−5.62	High	No	Yes	Yes
**9a**	10	−1.80	62.91	3.98	−4.77	High	Yes	No	No
Pearson coefficient	–0.81								
Spearman coefficient	–0.78								

## Data Availability

Data are contained within the article.

## Supplementary Materials

The following supporting information can be downloaded at https://www.mdpi.com/article/10.3390/biomimetics9010044/s1. Figures S1–S57: NMR spectrum. Figure S58: Structure of compound CHEMBL4094163. Figure S59: Two-dimensional maps of non-covalent interactions for the top-1 positions of docked ligands in HIV-RT non-nucleoside binding site. Table S1: The results of docking of the known non-nucleoside inhibitors of HIV-1 RT WT.
