# Supplementary material for: Bioinspired Pyrano[2,3-f]chromen-8-ones: Ring C-Opened Analogues of Calanolide A: Synthesis and Anti-HIV-1 Evaluation
Source: Biomimetics (Basel). 2024 Jan 11;9(1):44. doi: 10.3390/biomimetics9010044 (PMC10813249; doi:10.3390/biomimetics9010044)
Supplement: Supplementary file 1 [file biomimetics-09-00044-s001.zip › biomimetics-2788759-supplementary-final.pdf]

Supplementary Information

for

Bioinspired Pyrano[2,3-*f*]chromen-8-ones: Ring C-Opened Analogues of Calanolide A. Synthesis and Anti-HIV-1 Evaluation

Igor A. Khalymbadzha [1,*], Ramil F. Fatykhov [1], Ilya I. Butorin [1], Ainur D. Sharapov [1], Anastasia P. Potapova [1], Nibin Joy Muthipeedika [1], Grigory V. Zyryanov [1], Vsevolod V. Melekhin [1,2], Maria D. Tokhtueva [1], Sergey L. Deev [1], Marina K. Kukhanova [3], Nataliya N. Mochulskaya [1] and Mikhail V. Tsurkan [4,*]

[1] Department of Organic and Biomolecular Chemistry, Ural Federal University, 620002 Yekaterinburg, Russia; rf.fatykhov@urfu.ru (R.F.F.); iibutorin@urfu.ru (I.I.B.); a.d.sharapov@urfu.ru (A.D.S.); a.p.potapova@urfu.ru (A.P.P.); mnibinjoy@gmail.com (N.J.M.); g.v.zyrianov@urfu.ru (G.V.Z.); v.v.melekhin@urfu.ru (V.V.M.); maria.tokhtueva@urfu.ru (M.D.T.); deevsl@yandex.ru (S.L.D.); n.n.mochulskaia@urfu.ru (N.N.M.)

[2] Department of Medical Biology and Genetics, Ural State Medical University, 620028 Yekaterinburg, Russia

[3] Engelhardt Institute of Molecular Biology, 119991 Moscow, Russia; kukhan86@hotmail.com

[4] Leibniz Institute of Polymer Research Dresden, 01069 Dresden, Germany

* Correspondence: i.a.khalymbadzha@urfu.ru (I.A.K.); tsurkan@ipfdd.de (M.V.T.)

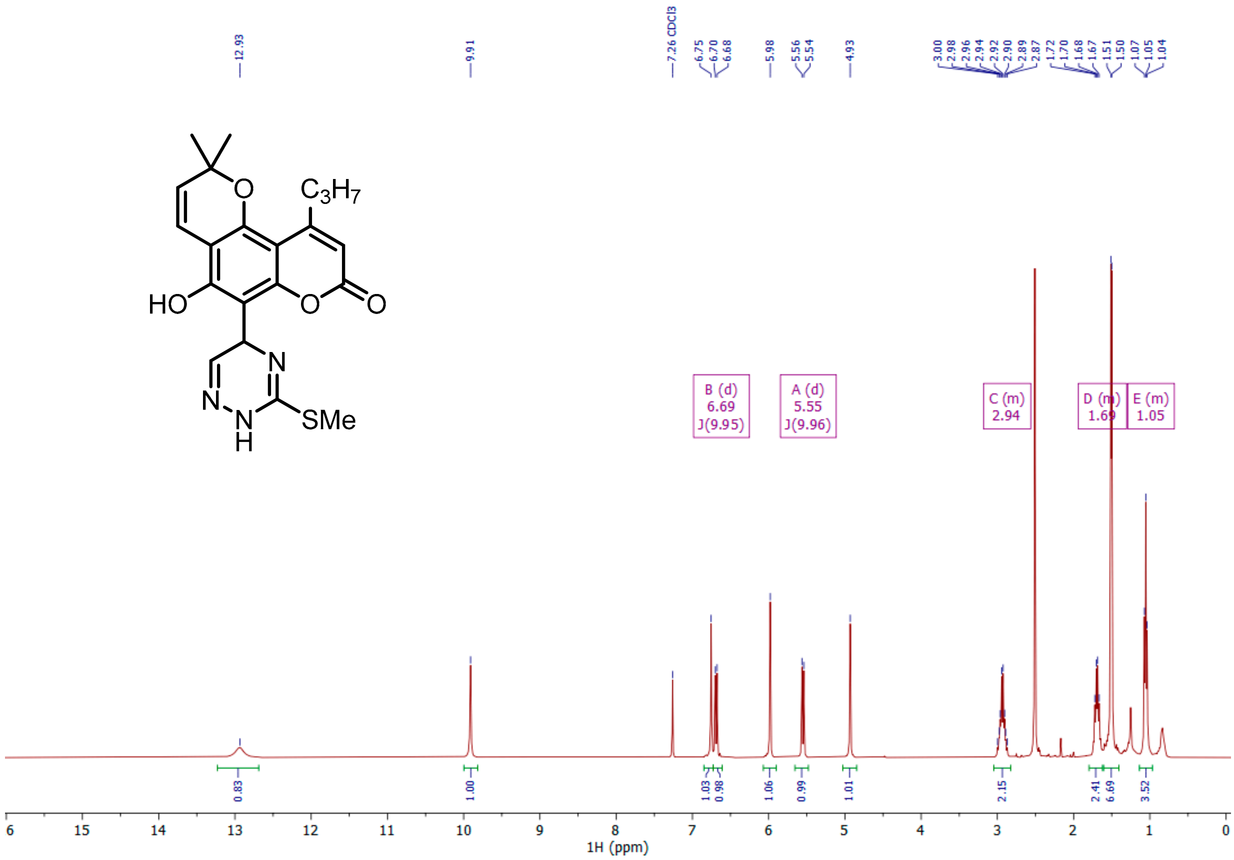

Supplementary Figure S1. ^{1}H **NMR spectrum of 4a**

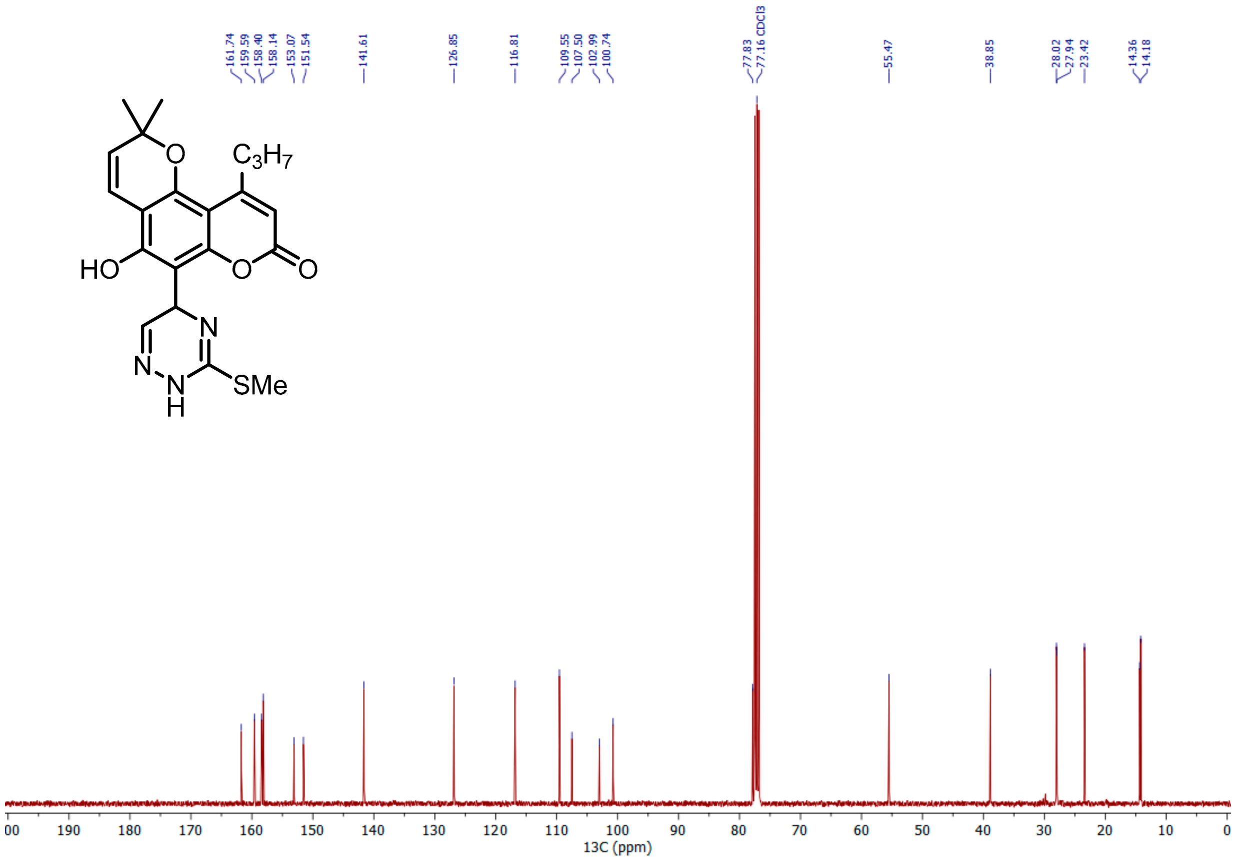

Supplementary Figure S2. ^{13}C **NMR spectrum of 4a**

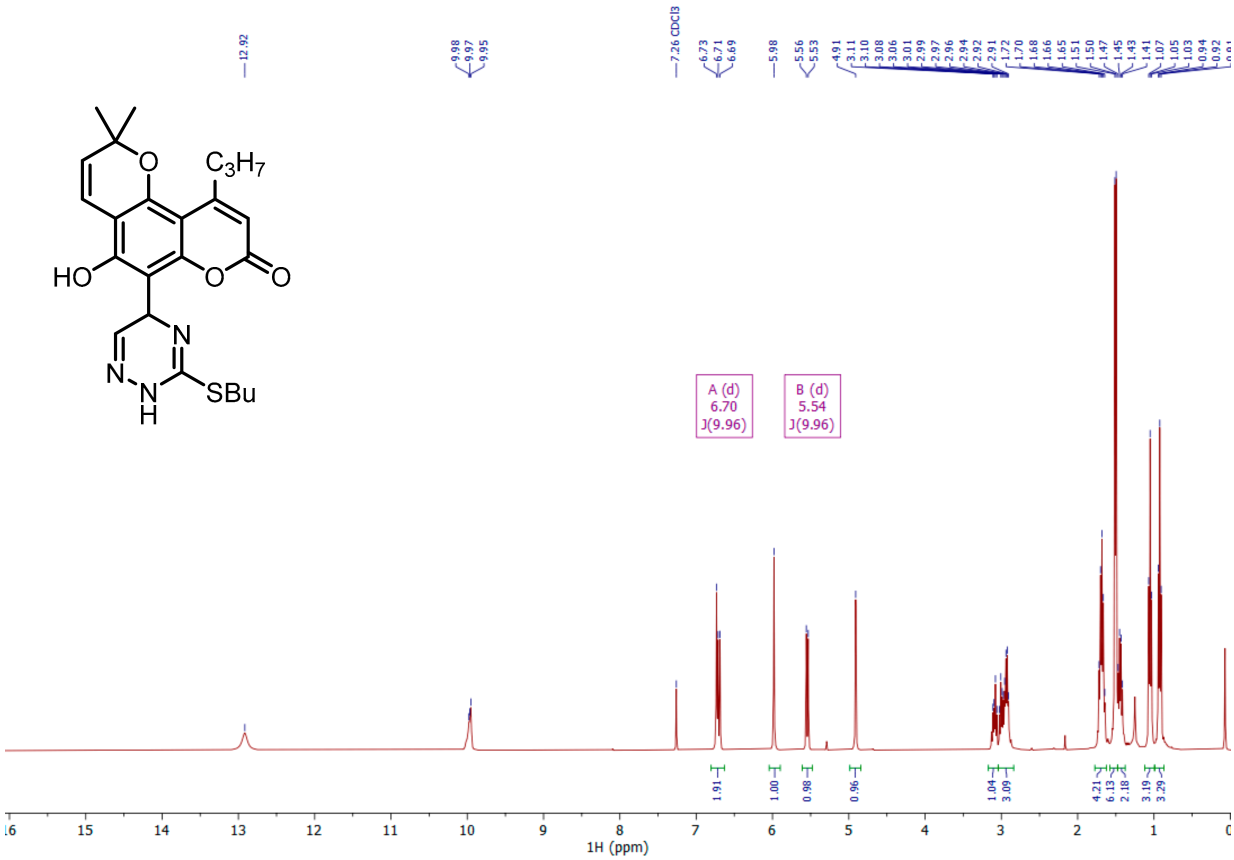

Supplementary Figure S3. ^{1}**H NMR spectrum of 4b**

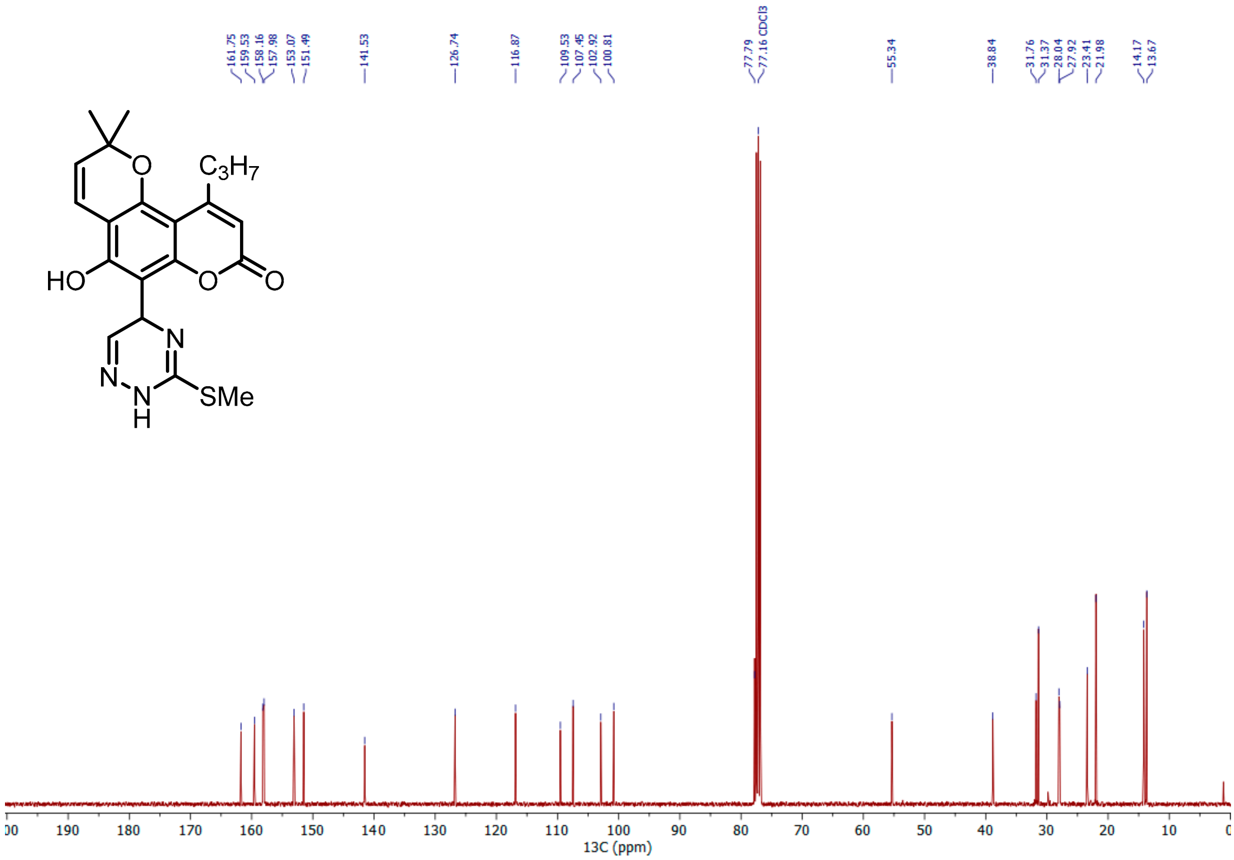

Supplementary Figure S4. ^{13}**C NMR spectrum of 4b**

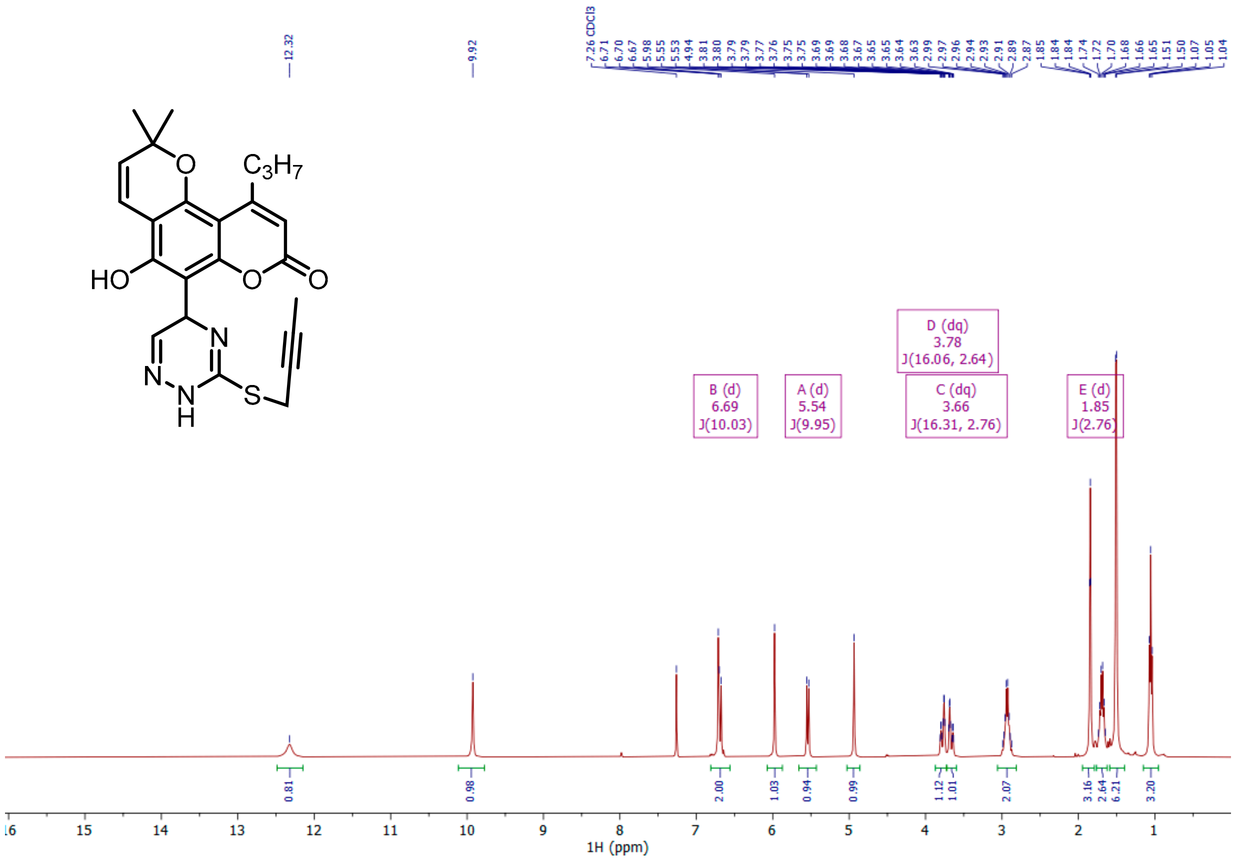

Supplementary Figure S5. ^{1}H NMR spectrum of 4c

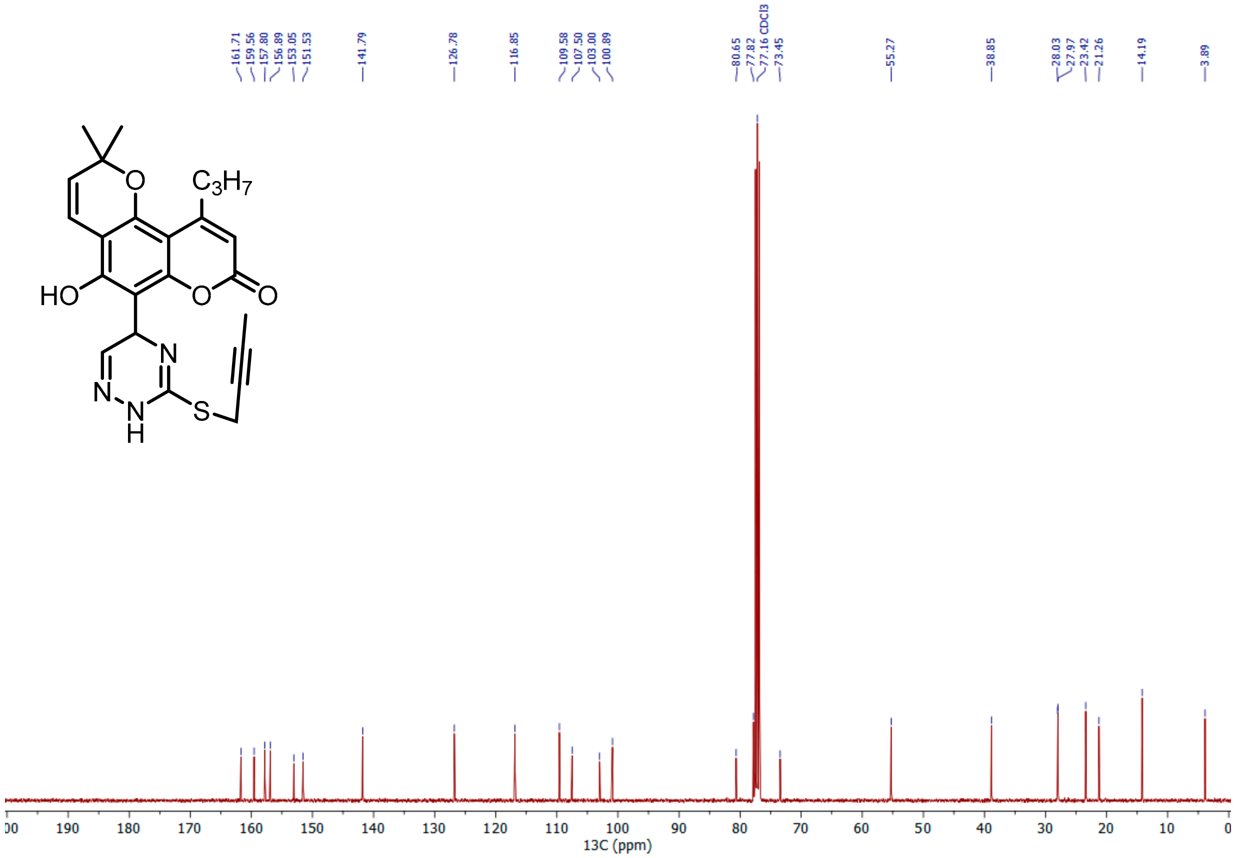

Supplementary Figure S6. ^{13}C NMR spectrum of 4c

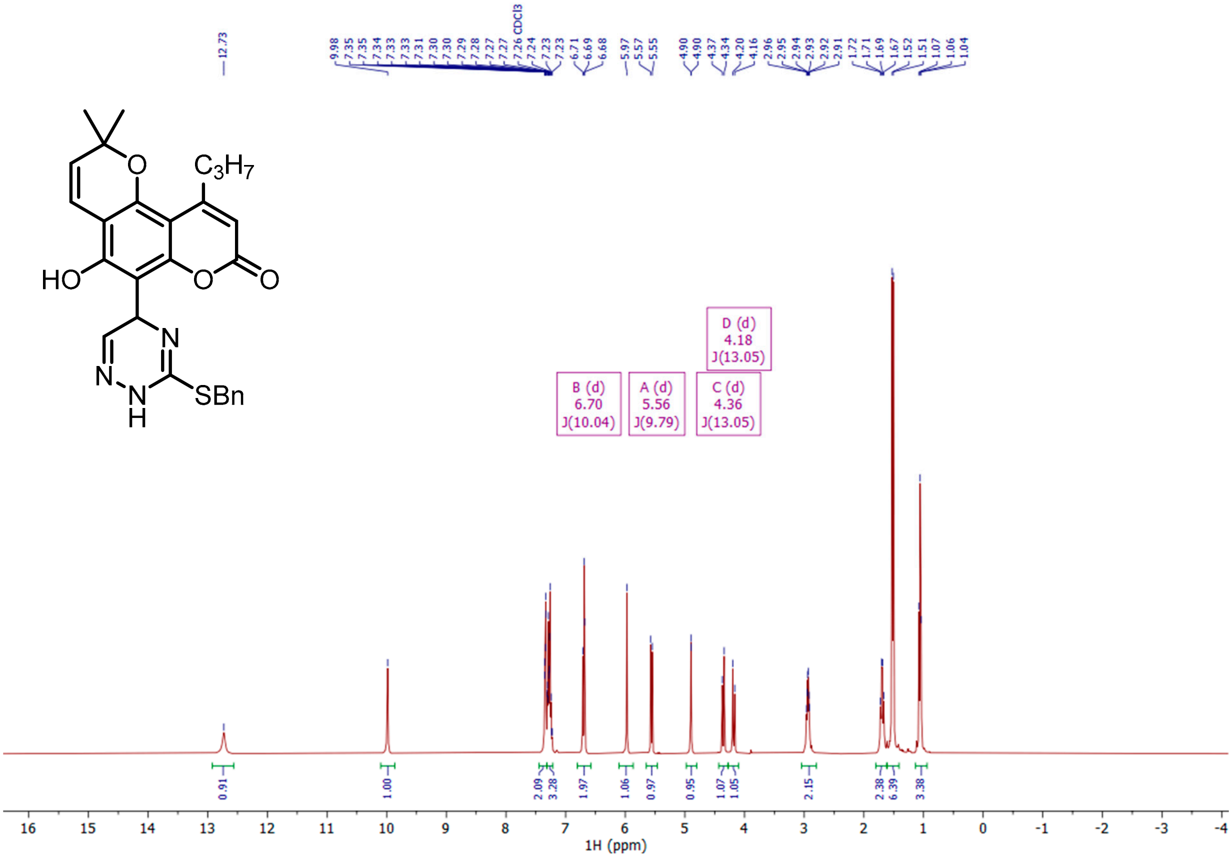

Supplementary Figure S7. ^{1}H NMR spectrum of 4d

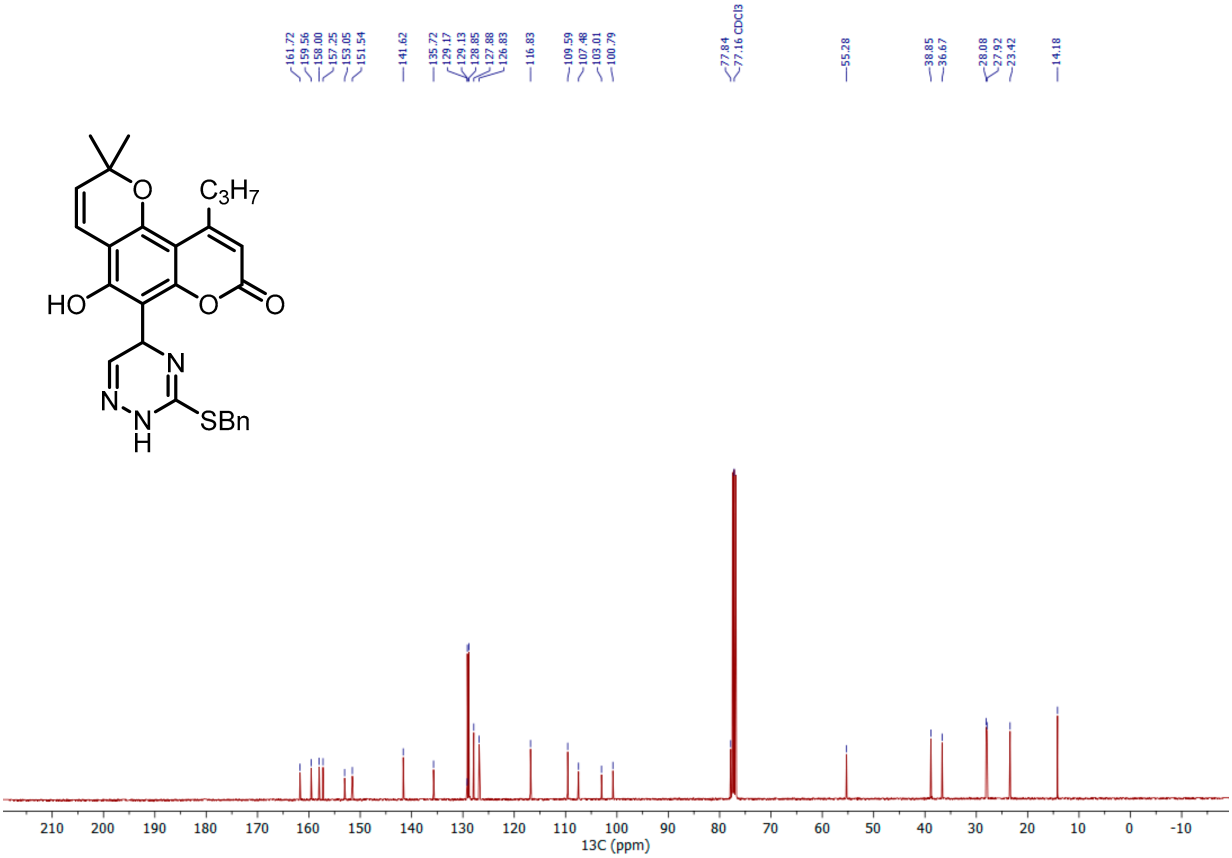

Supplementary Figure S8. ^{13}C NMR spectrum of 4d

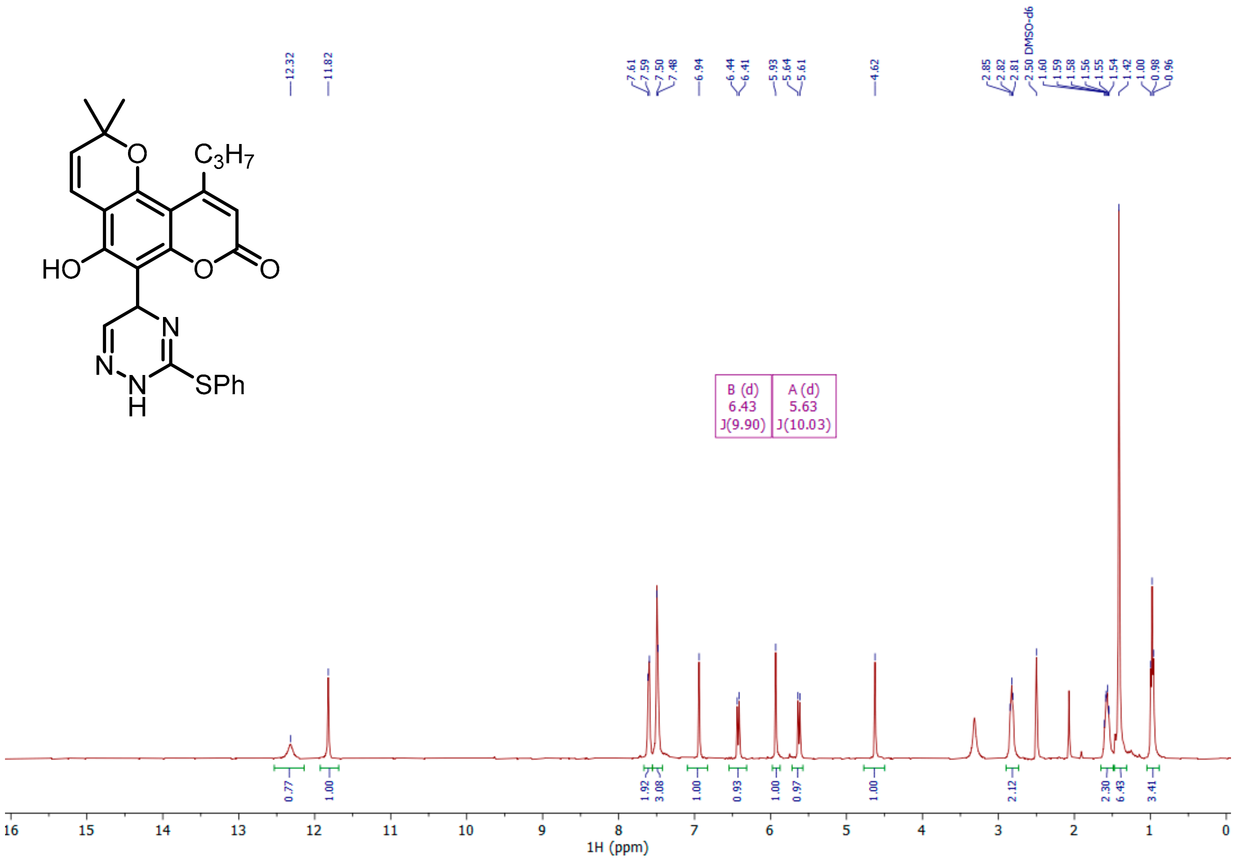

Supplementary Figure S9. ^{1}H NMR spectrum of 4e

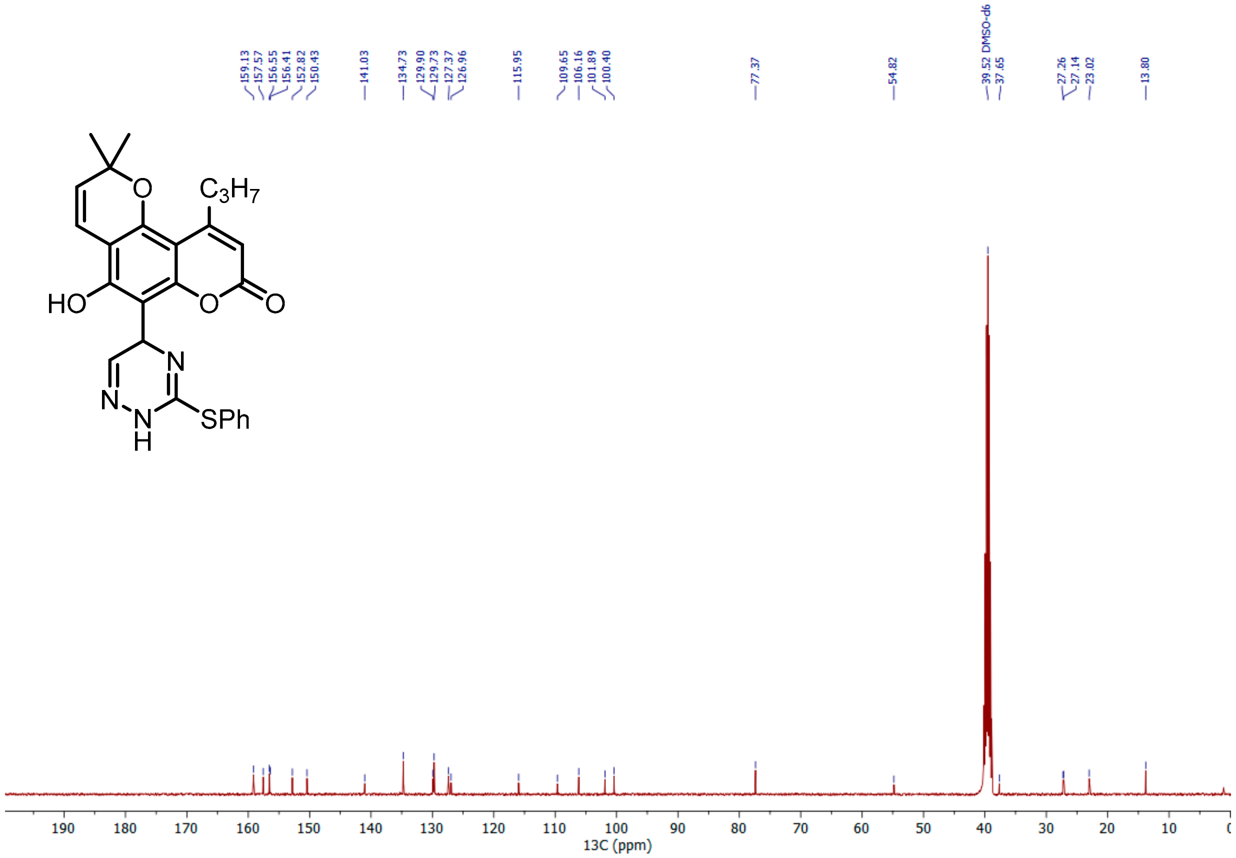

Supplementary Figure S10. ^{13}C NMR spectrum of 4e

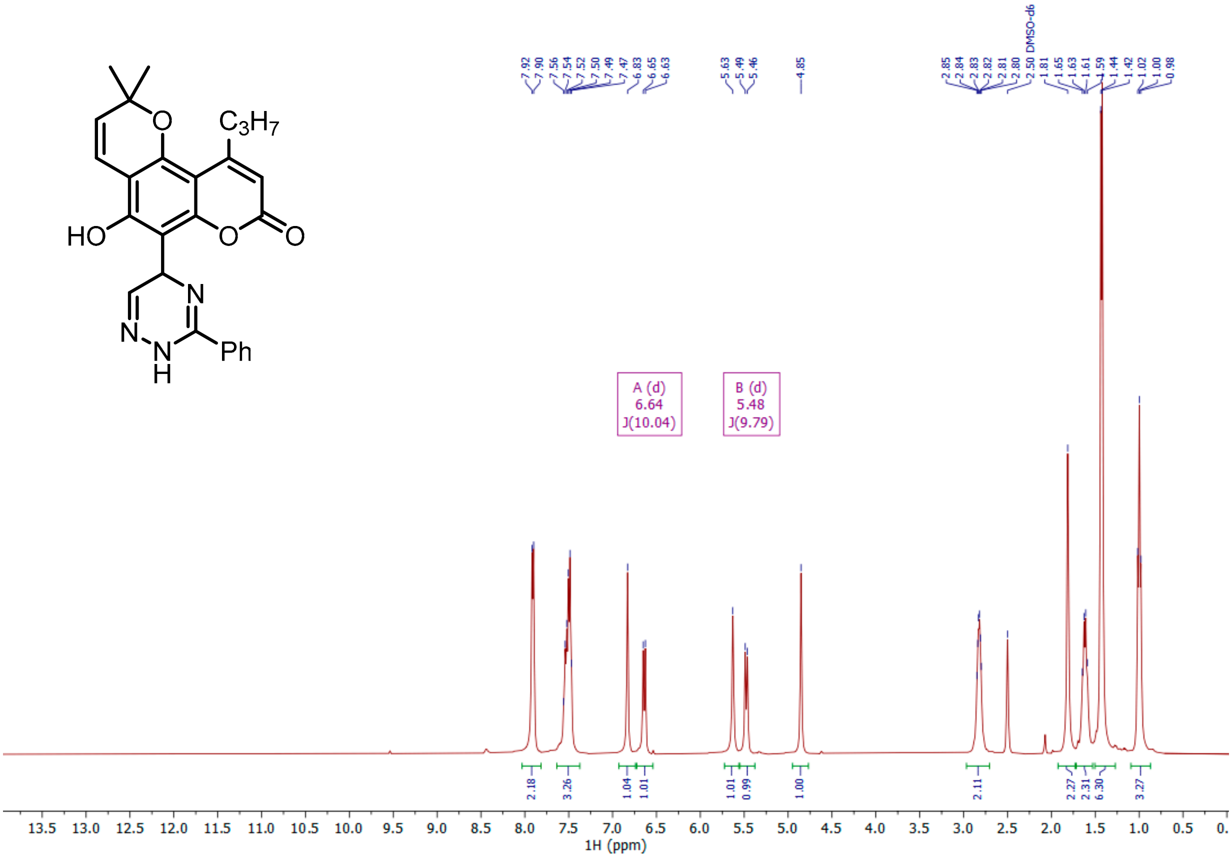

Supplementary Figure S11. ^{1}**H NMR spectrum of 4f**

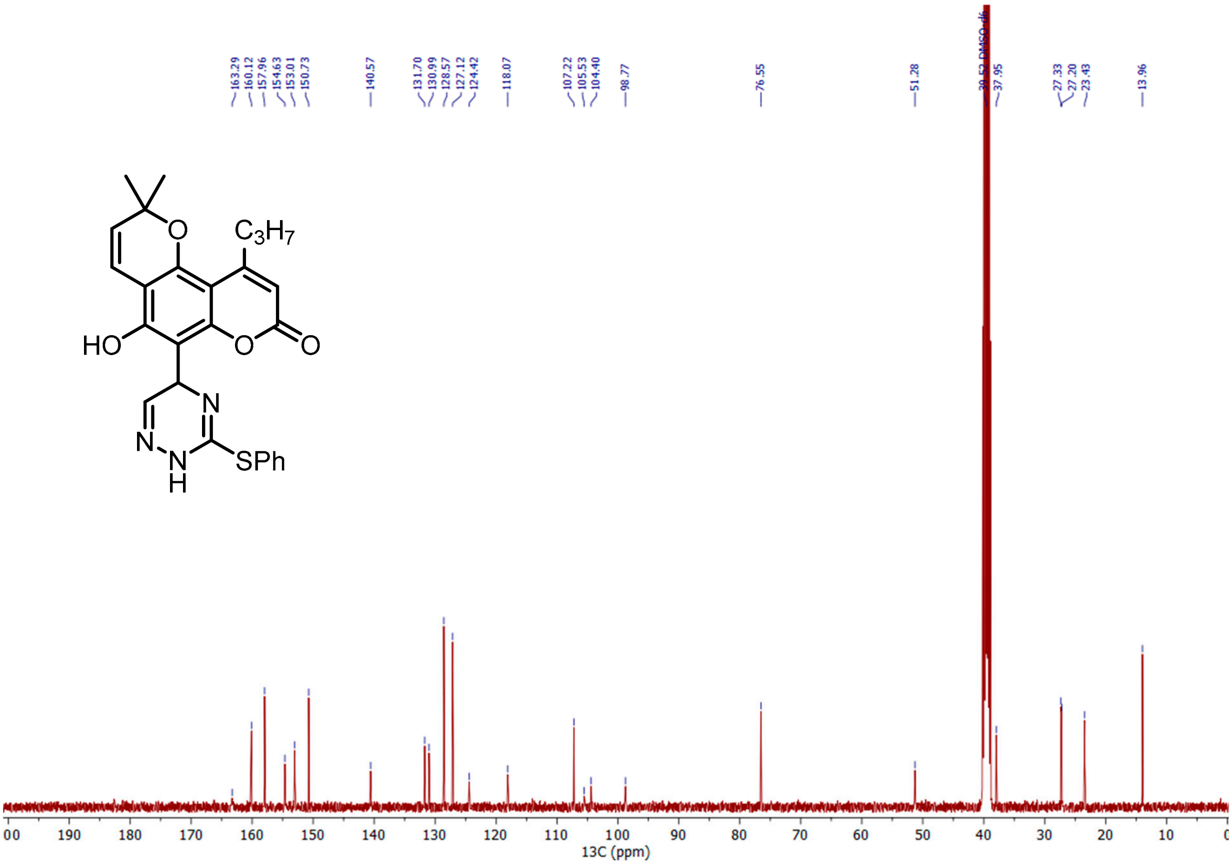

Supplementary Figure S12. ^{13}**C NMR spectrum of 4f**

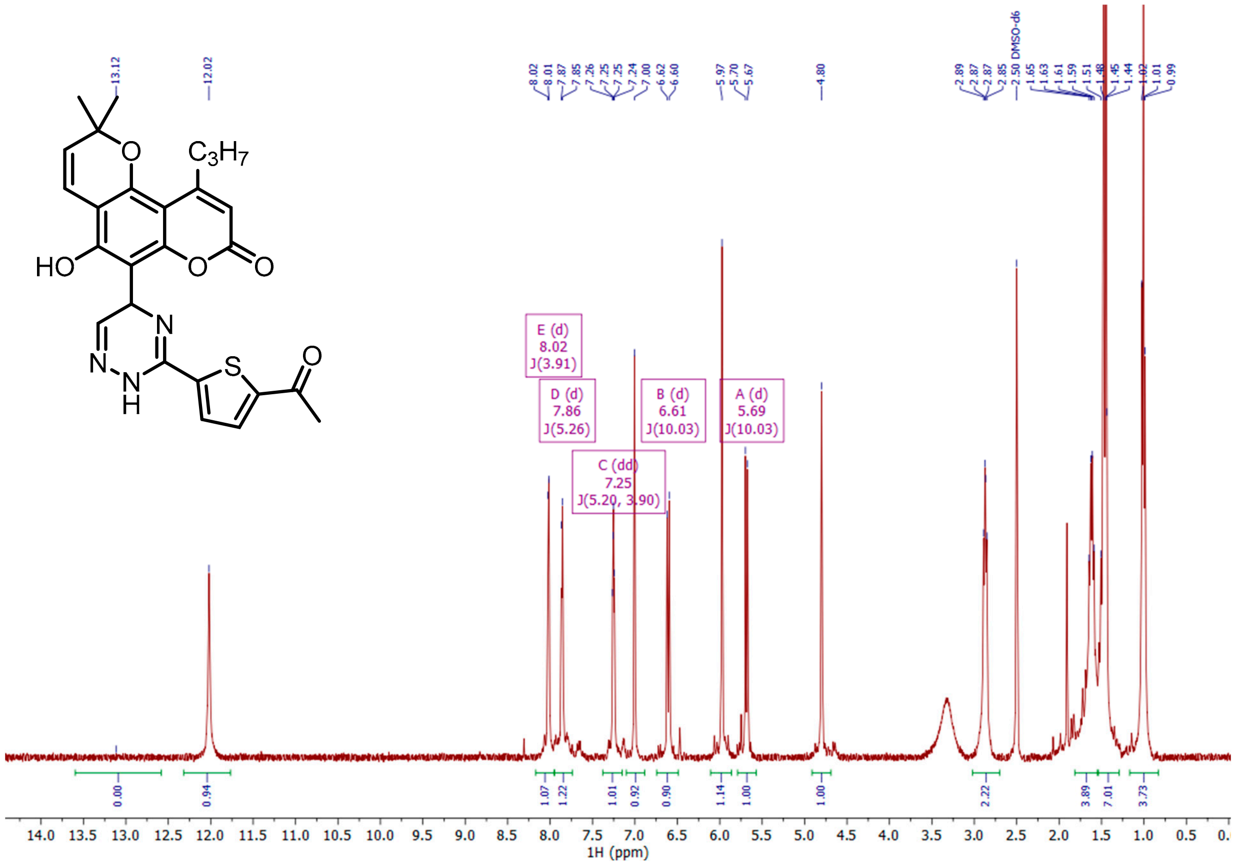

Supplementary Figure S13. ^{1}H NMR spectrum of 4g

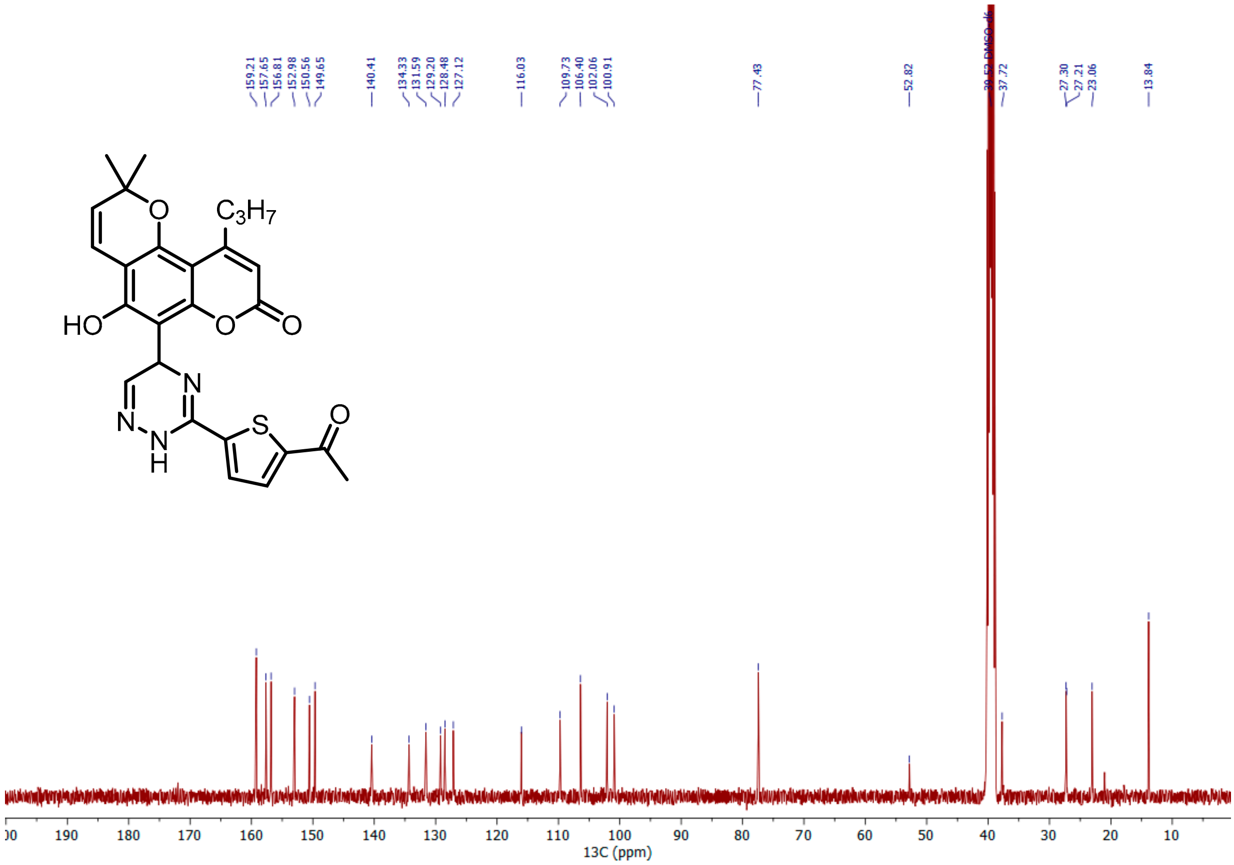

Supplementary Figure S14. ^{13}C NMR spectrum of 4g

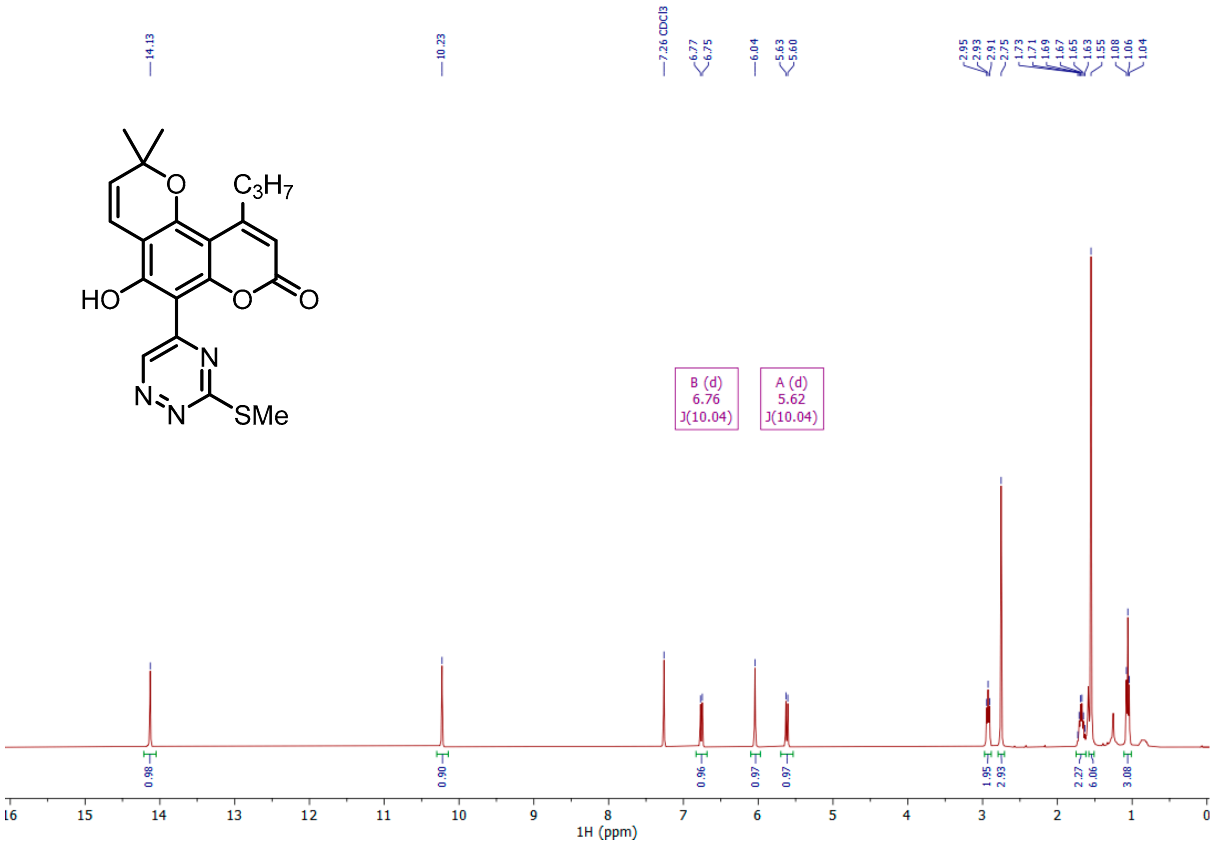

Supplementary Figure S15. ^{1}**H NMR spectrum of 5a**

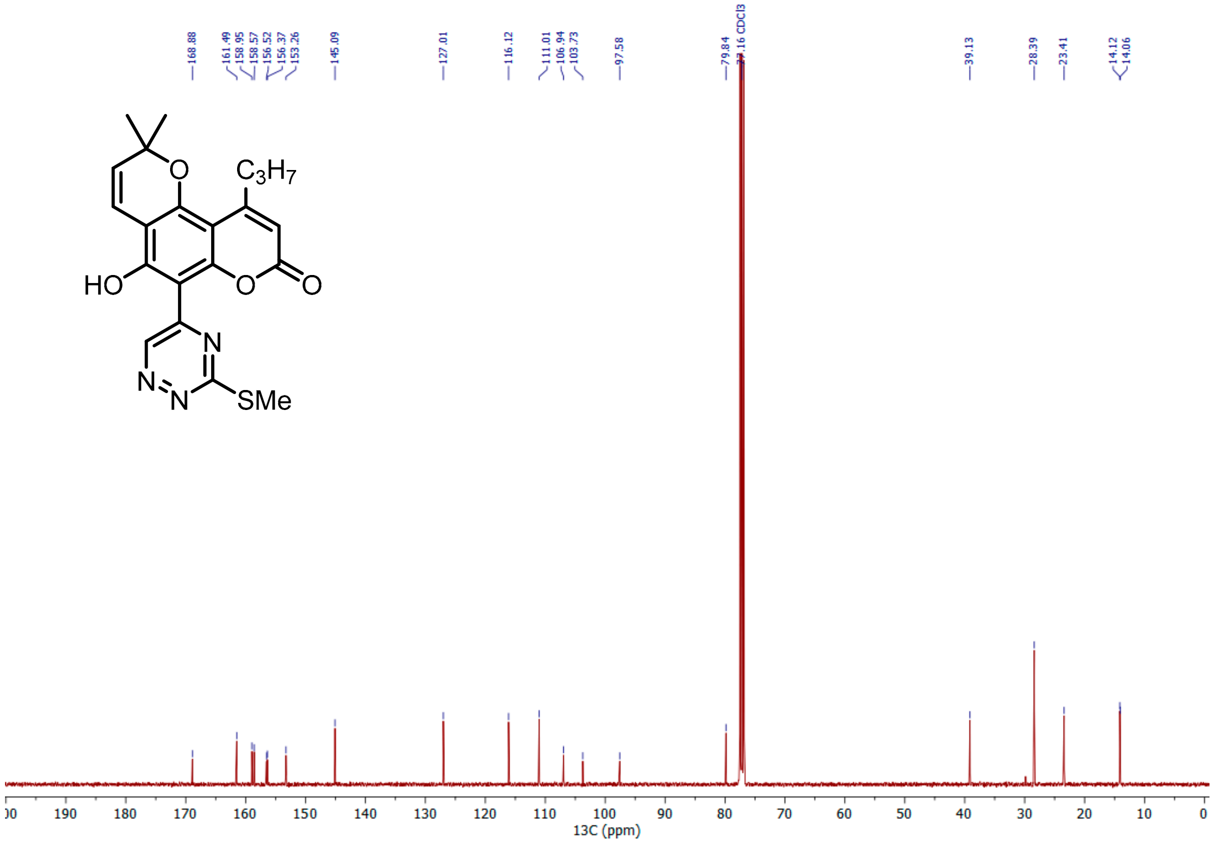

Supplementary Figure S16. ^{13}**C NMR spectrum of 5a**

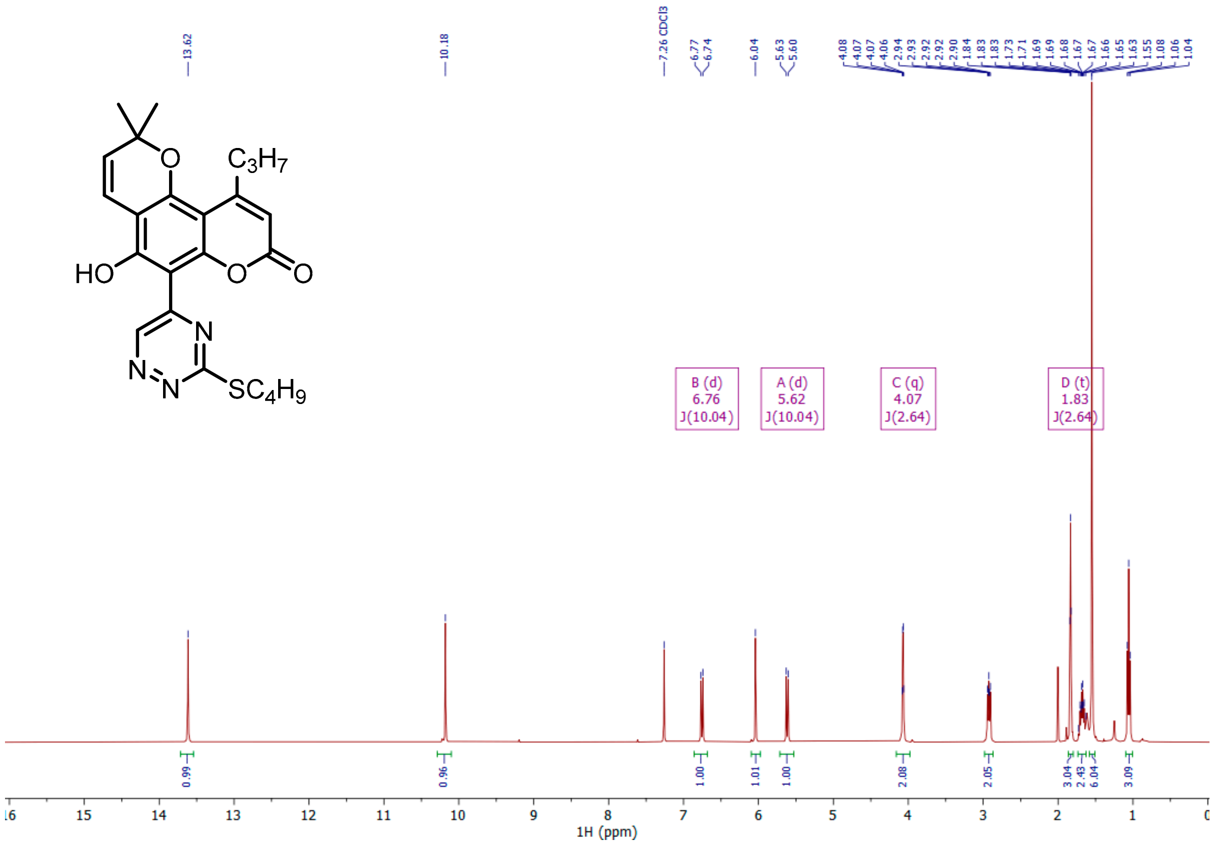

Supplementary Figure S17. ^{1}H NMR spectrum of 5b

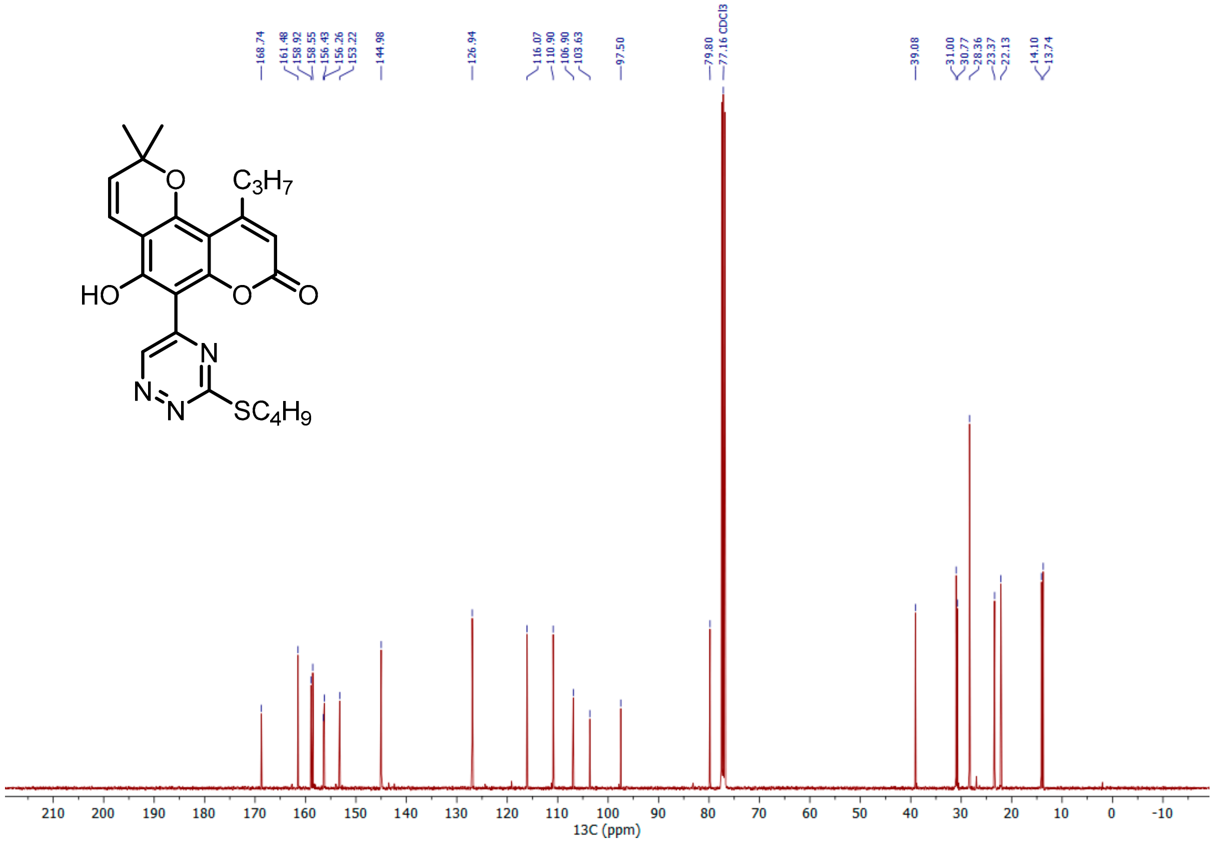

Supplementary Figure S18. ^{13}C NMR spectrum of 5b

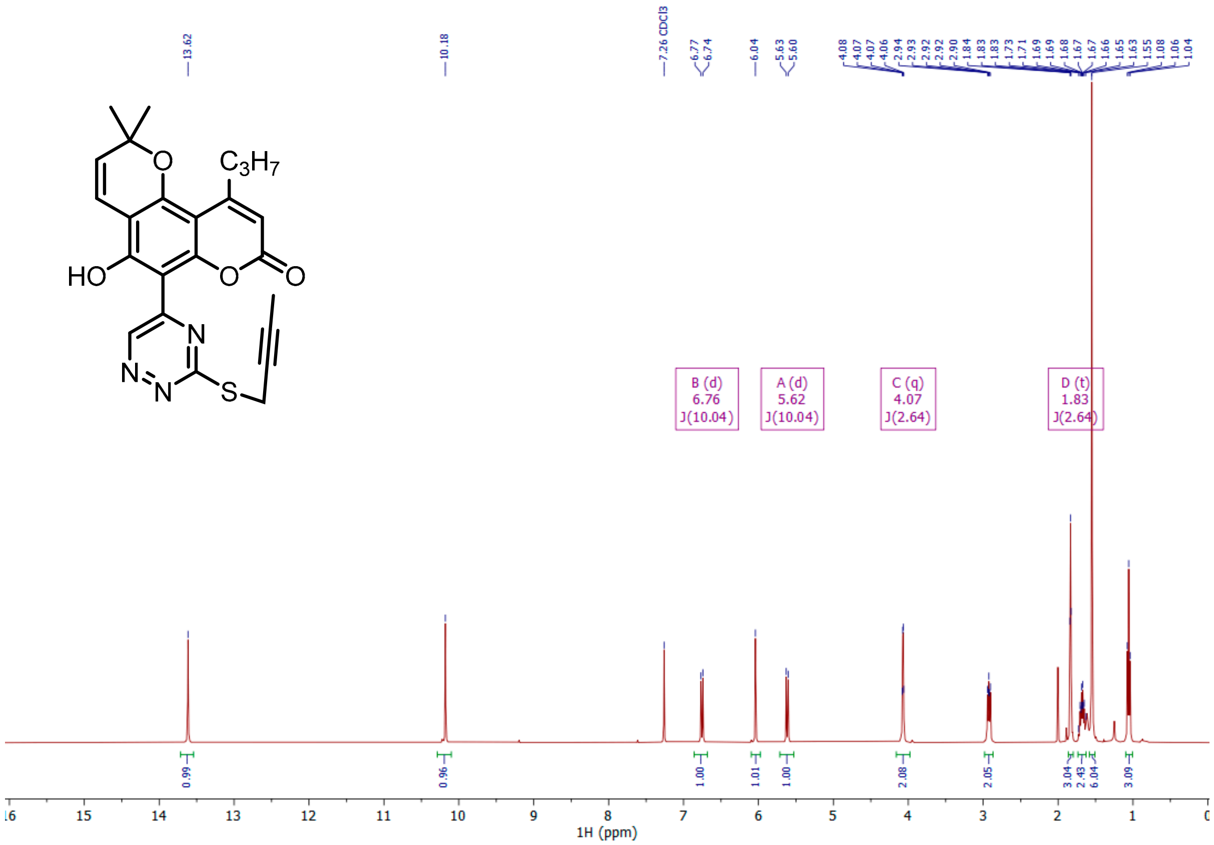

Supplementary Figure S19. ^{1}H NMR spectrum of 5c

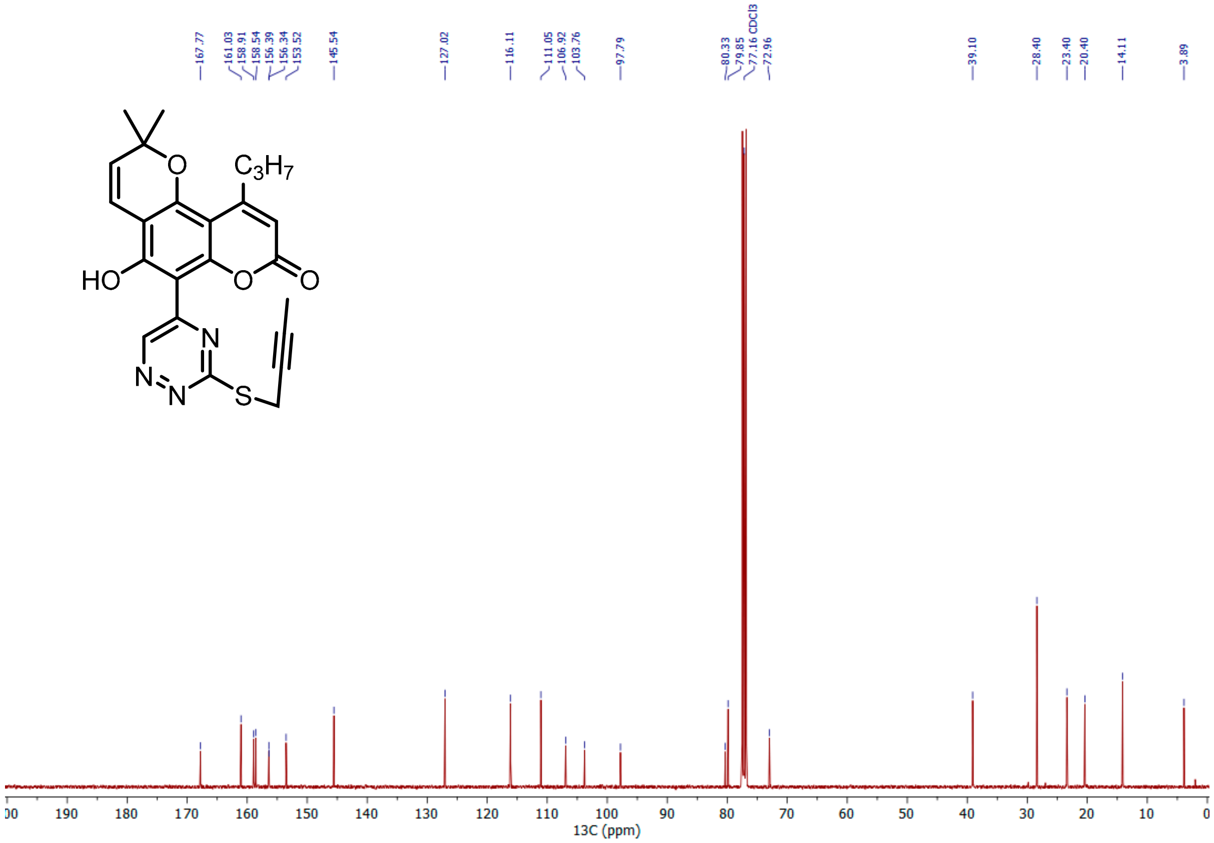

Supplementary Figure S20. ^{13}C NMR spectrum of 5c

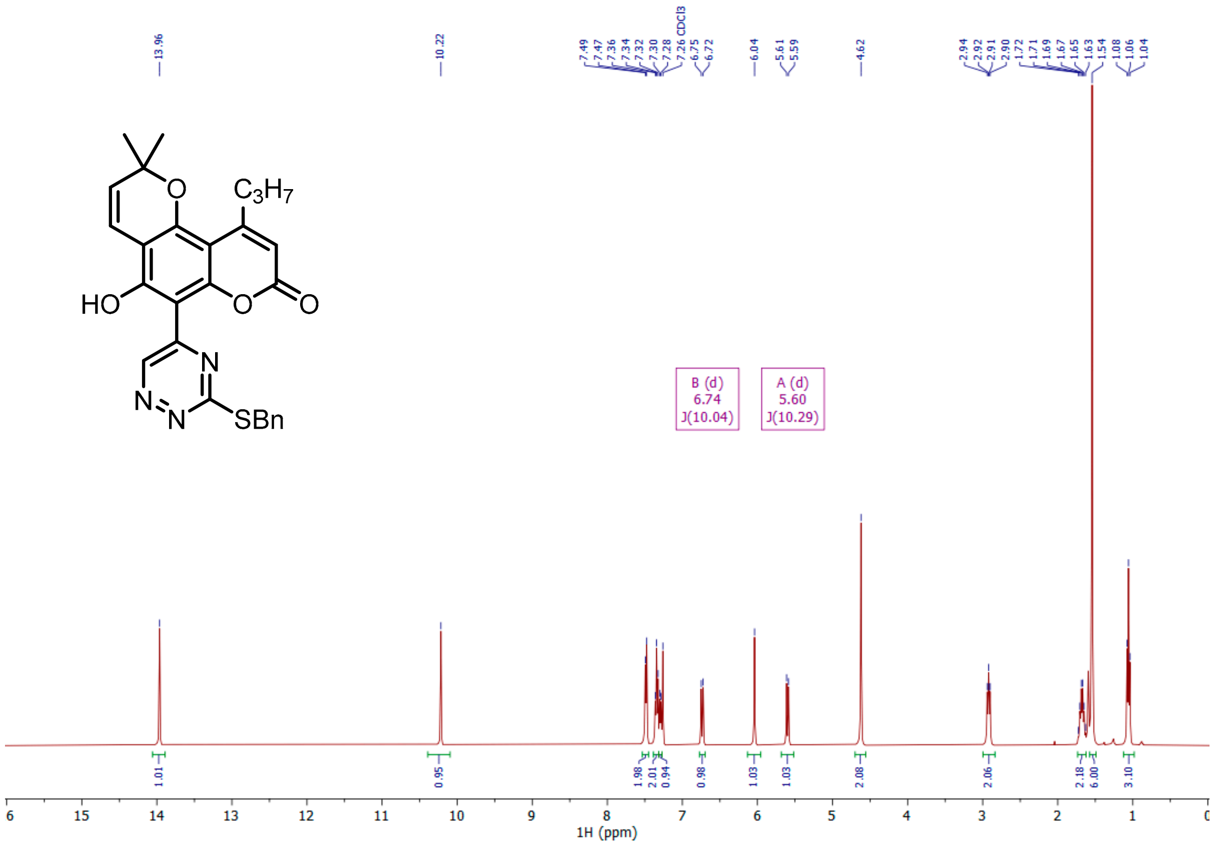

Supplementary Figure S21. ^{1}H NMR spectrum of 5d

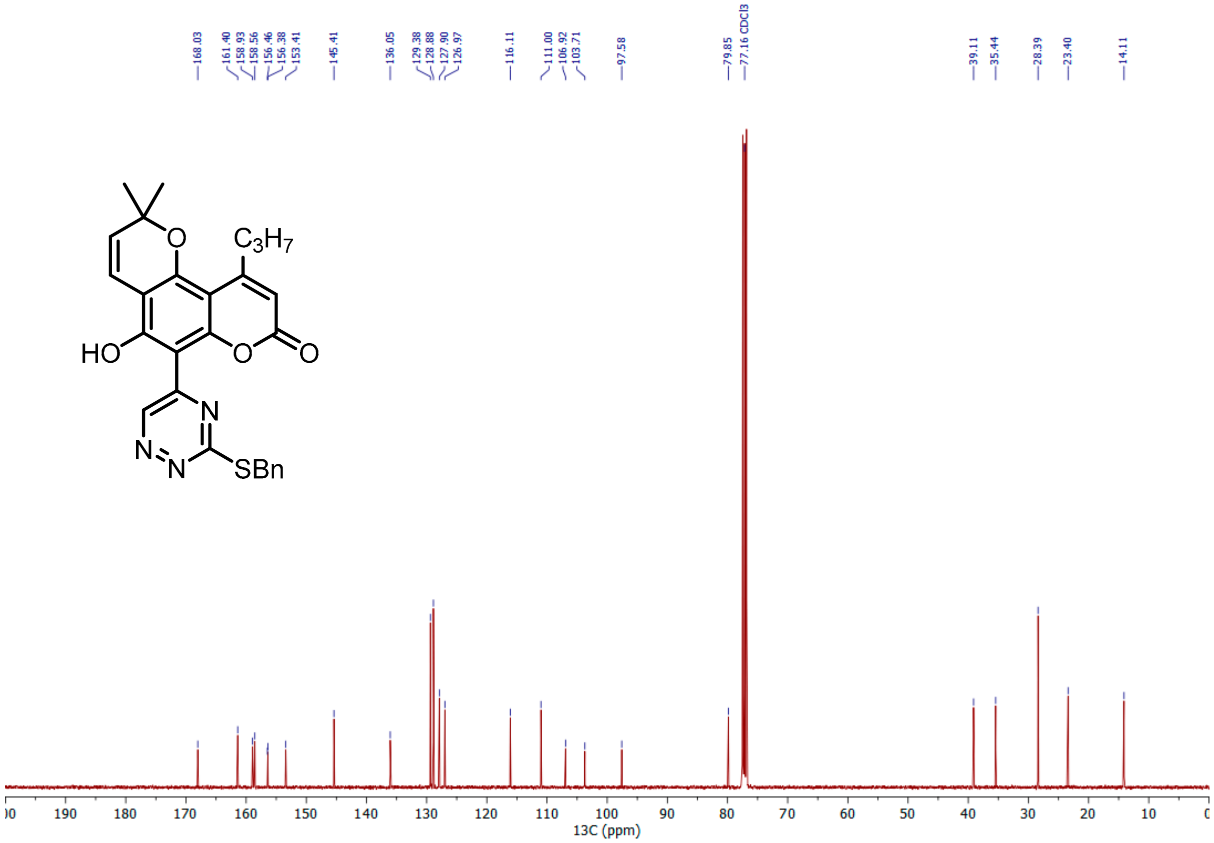

Supplementary Figure S22. ^{13}C NMR spectrum of 5d

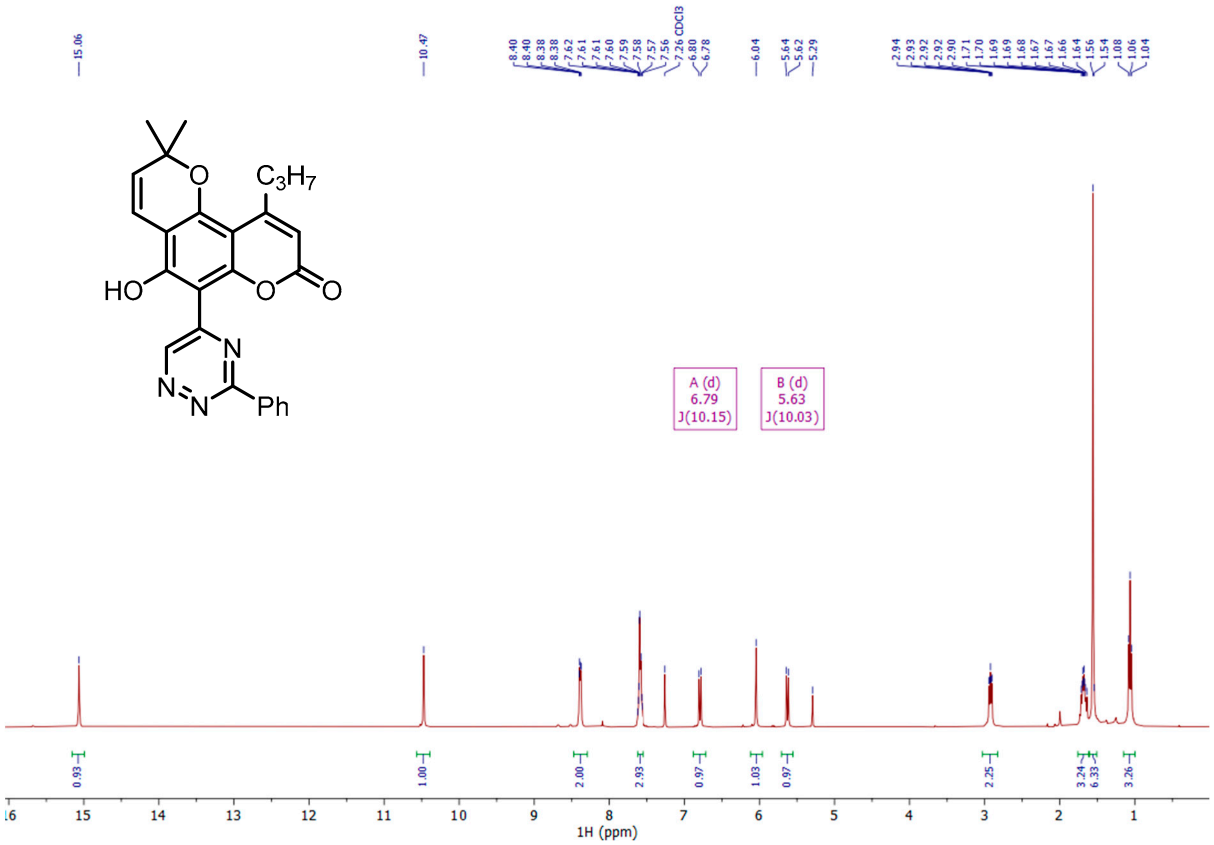

Supplementary Figure S23. ^{1}H NMR spectrum of 5f

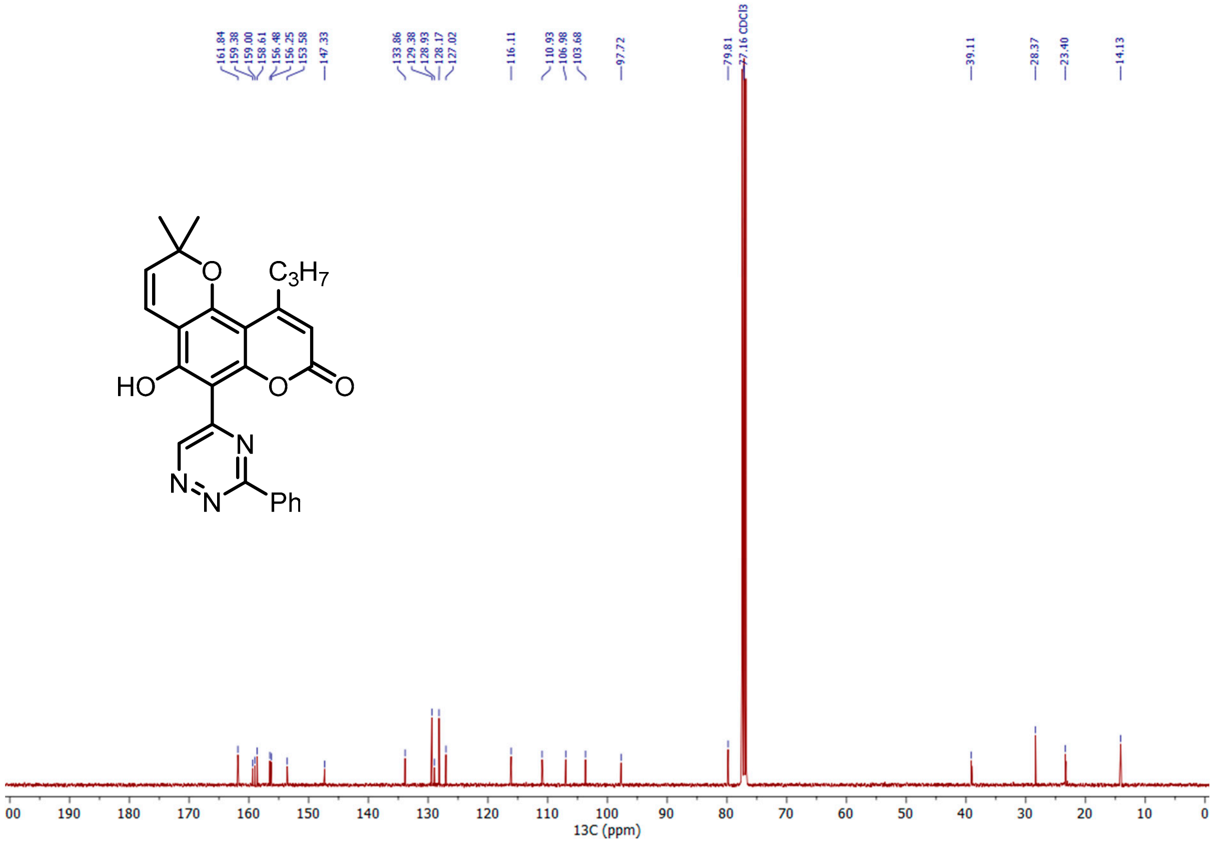

Supplementary Figure S24. ^{13}C NMR spectrum of 5f

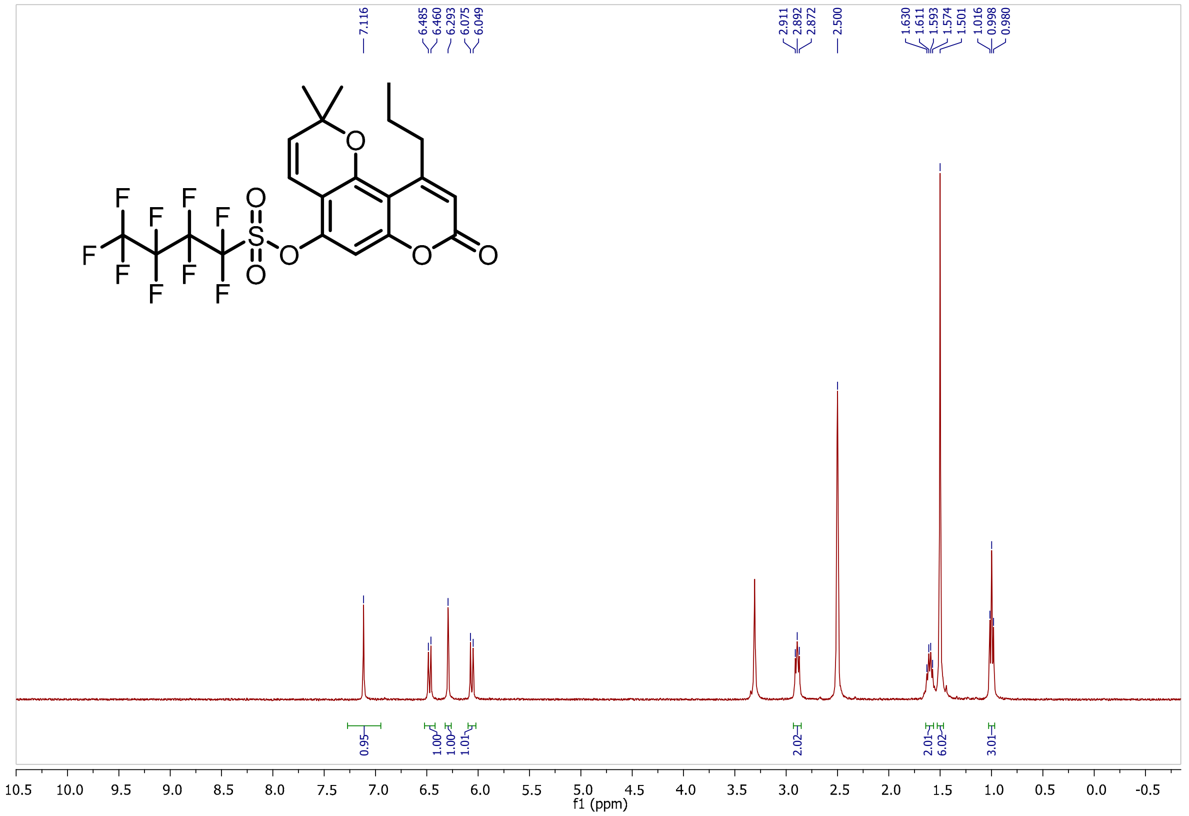

Supplementary Figure S25. ^{1}**H NMR spectrum of 8**

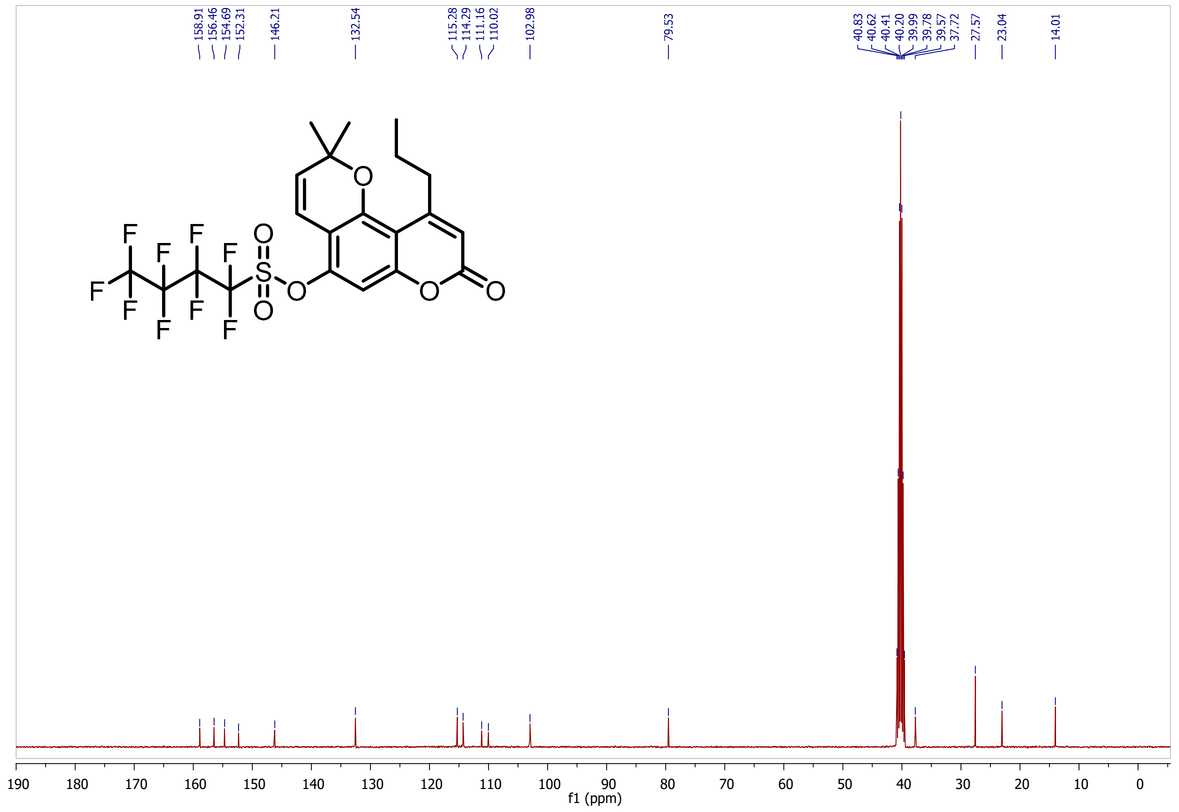

Supplementary Figure S26. ^{13}**C NMR spectrum of 8**

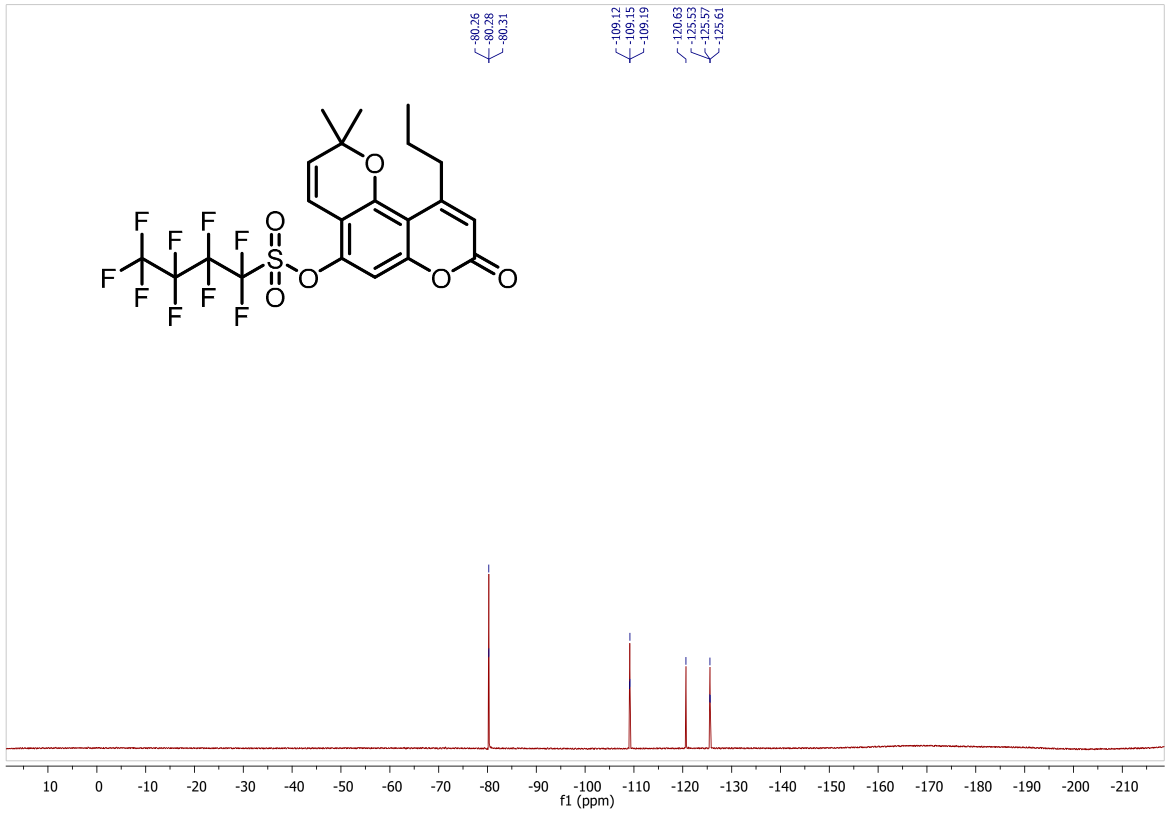

Supplementary Figure S27. ^{19}**F NMR spectrum of 8**

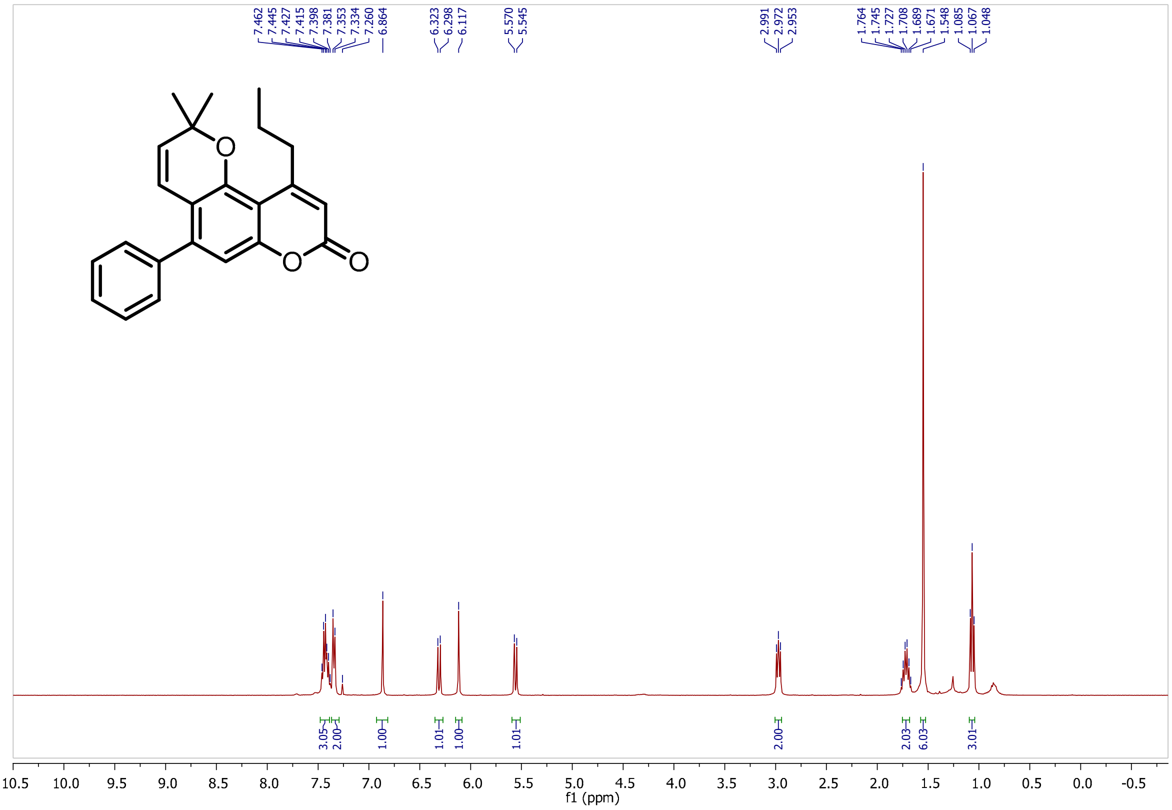

Supplementary Figure S28. ^{1}**H NMR spectrum of 7a**

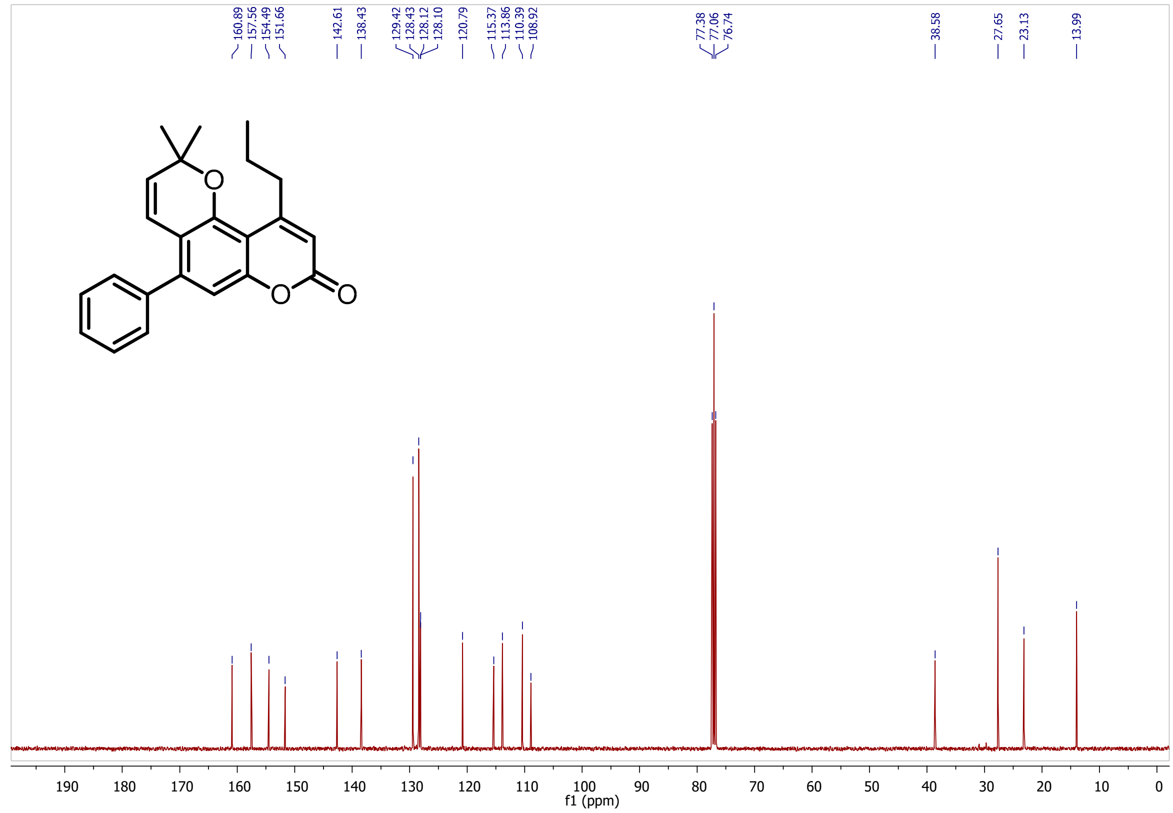

Supplementary Figure S29. ^{13}C NMR spectrum of 7a

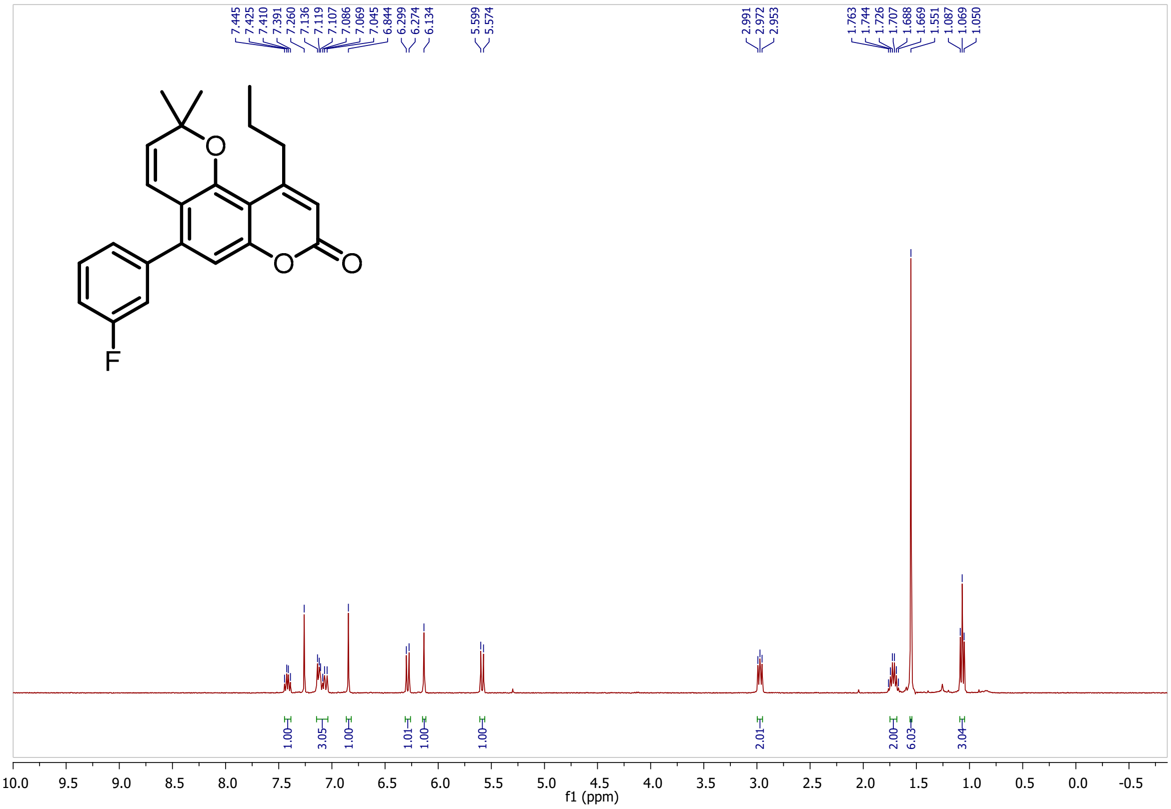

Supplementary Figure S30. ^{1}H NMR spectrum of 7b

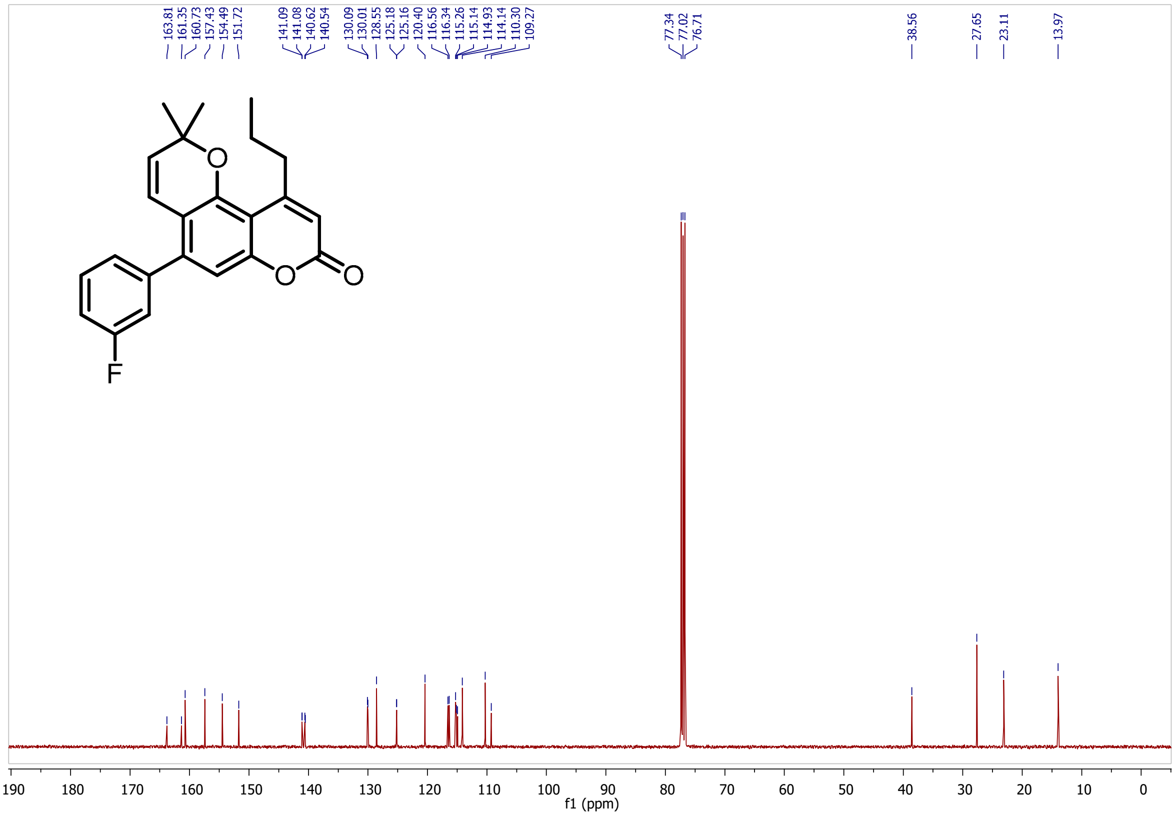

Supplementary Figure S31. ^{13}C **NMR spectrum of 7b**

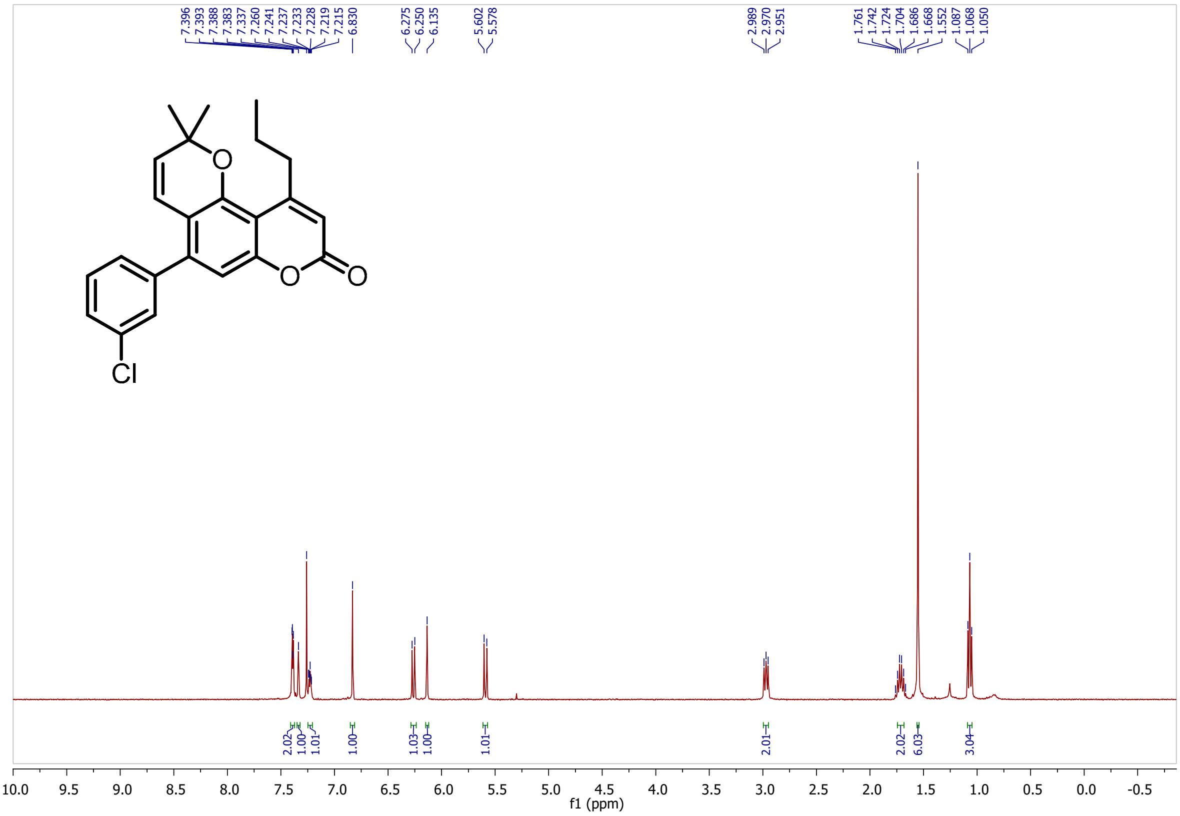

Supplementary Figure S32. ^{1}H **NMR spectrum of 7c**

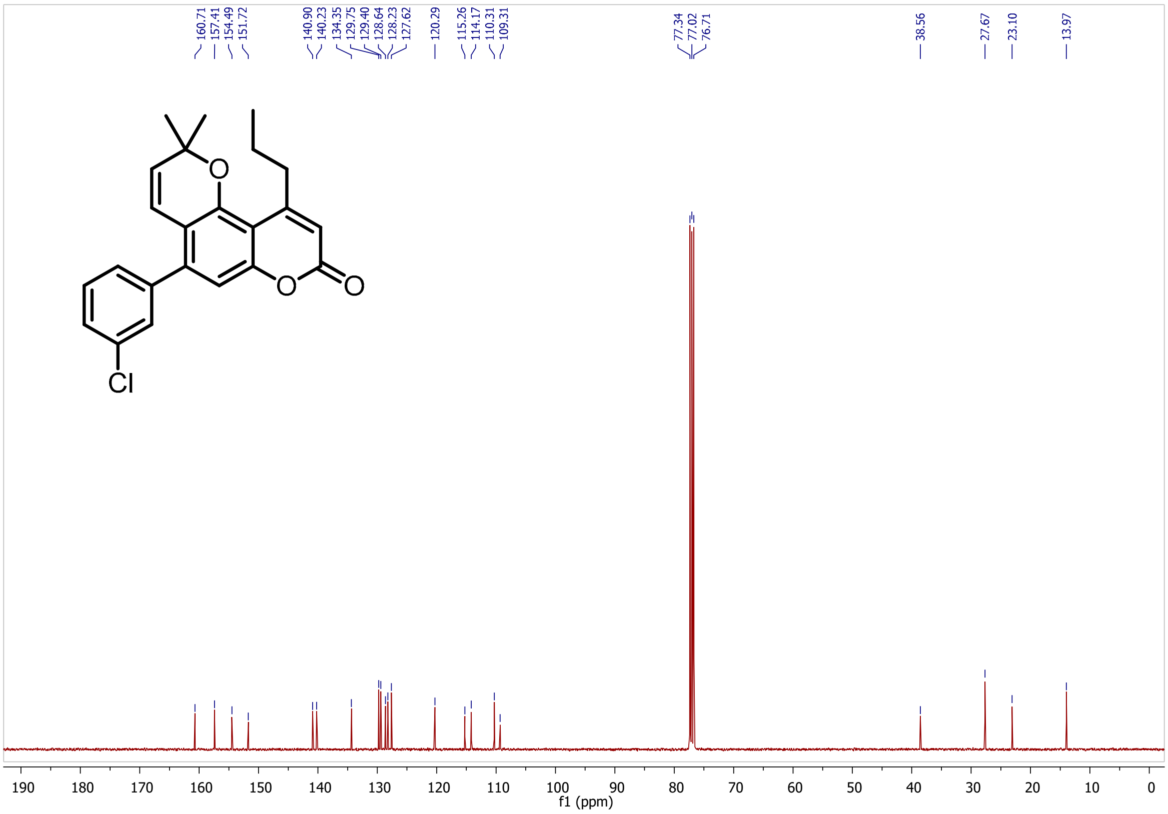

Supplementary Figure S33. ^{13}**C NMR spectrum of 7c**

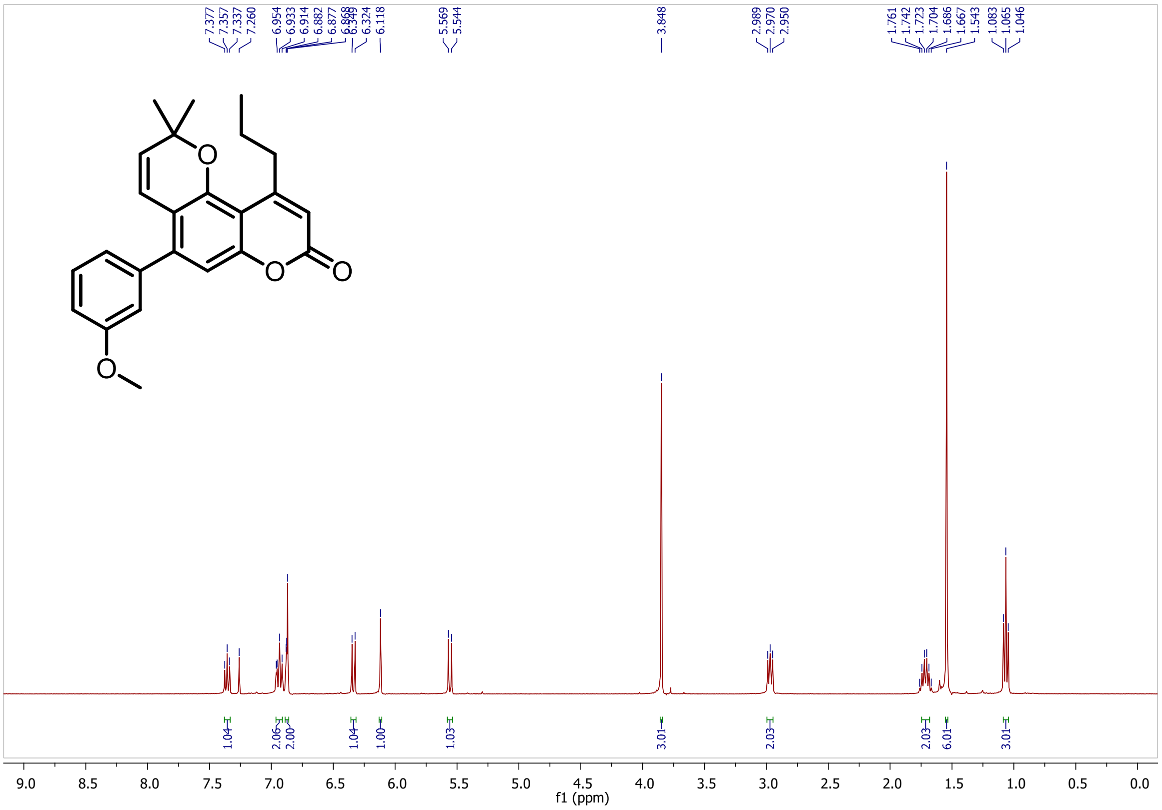

Supplementary Figure S34. ^{1}**H NMR spectrum of 7d**

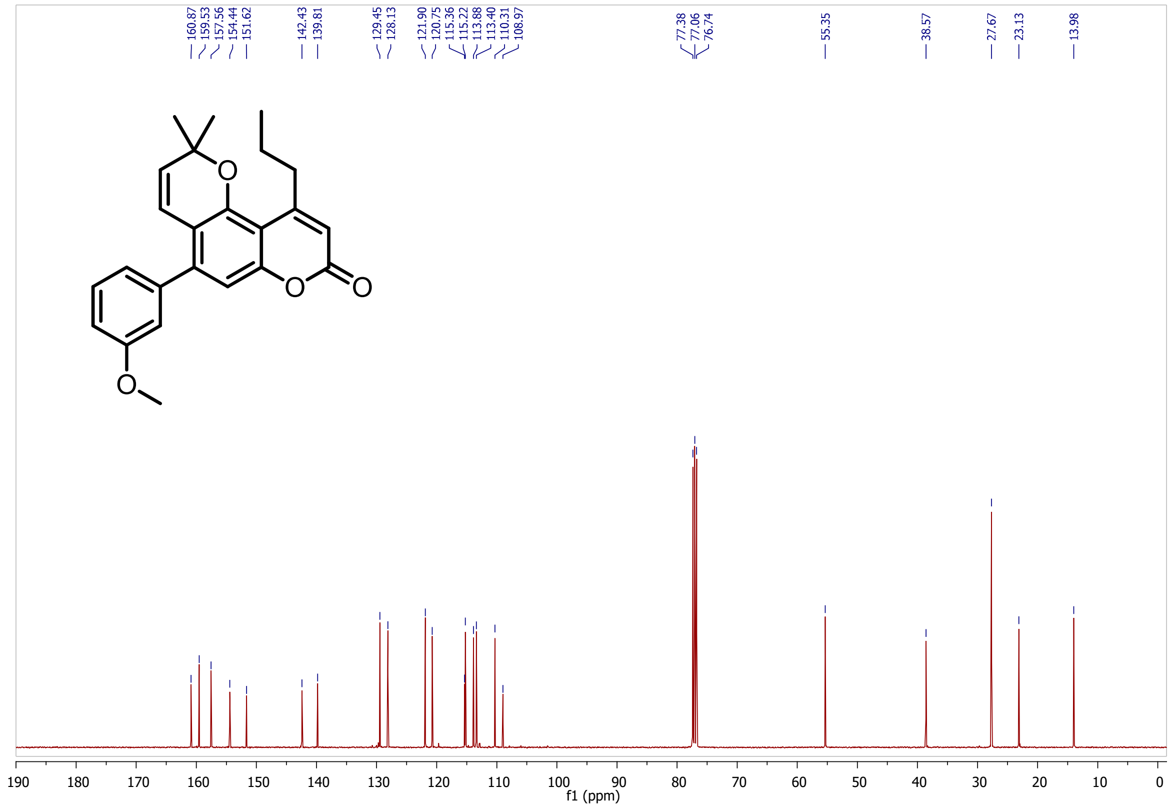

Supplementary Figure S35. ^{13}C NMR spectrum of 7d

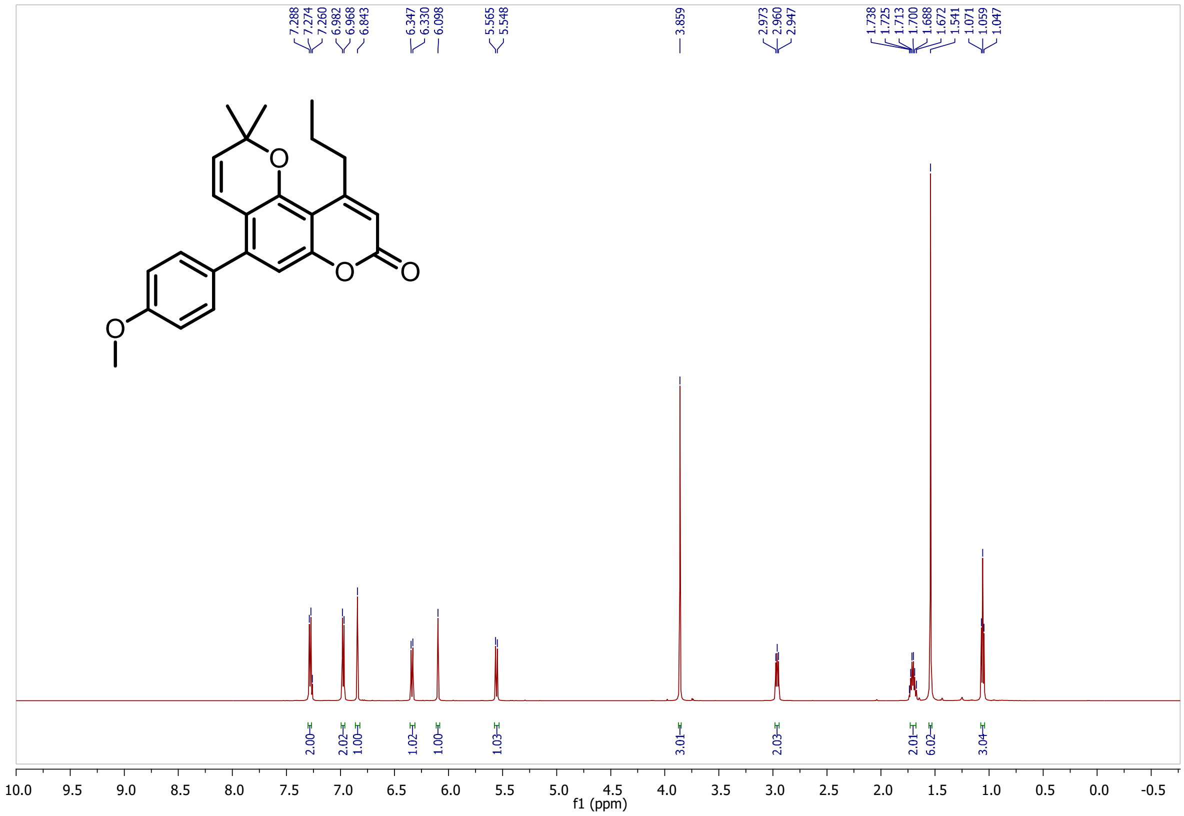

Supplementary Figure S36. ^{1}H NMR spectrum of 7e

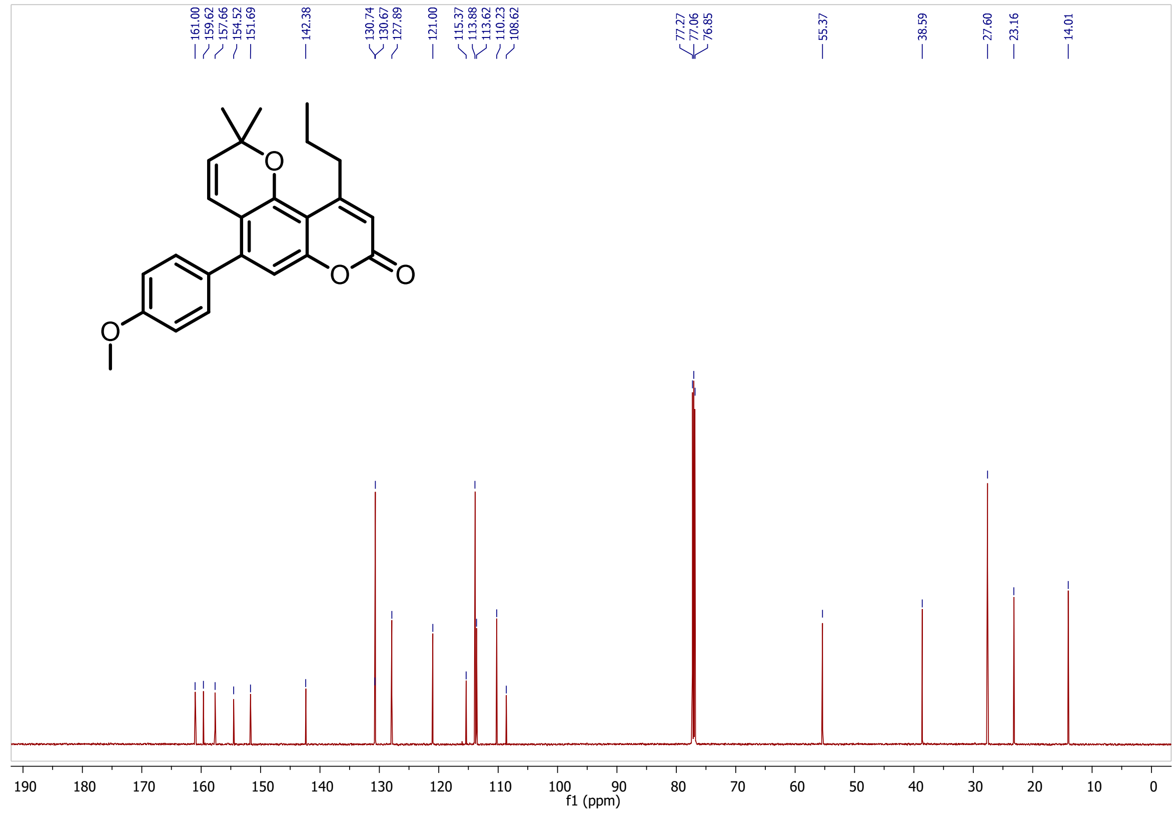

Supplementary Figure S37. ^{13}**C NMR spectrum of 7e**

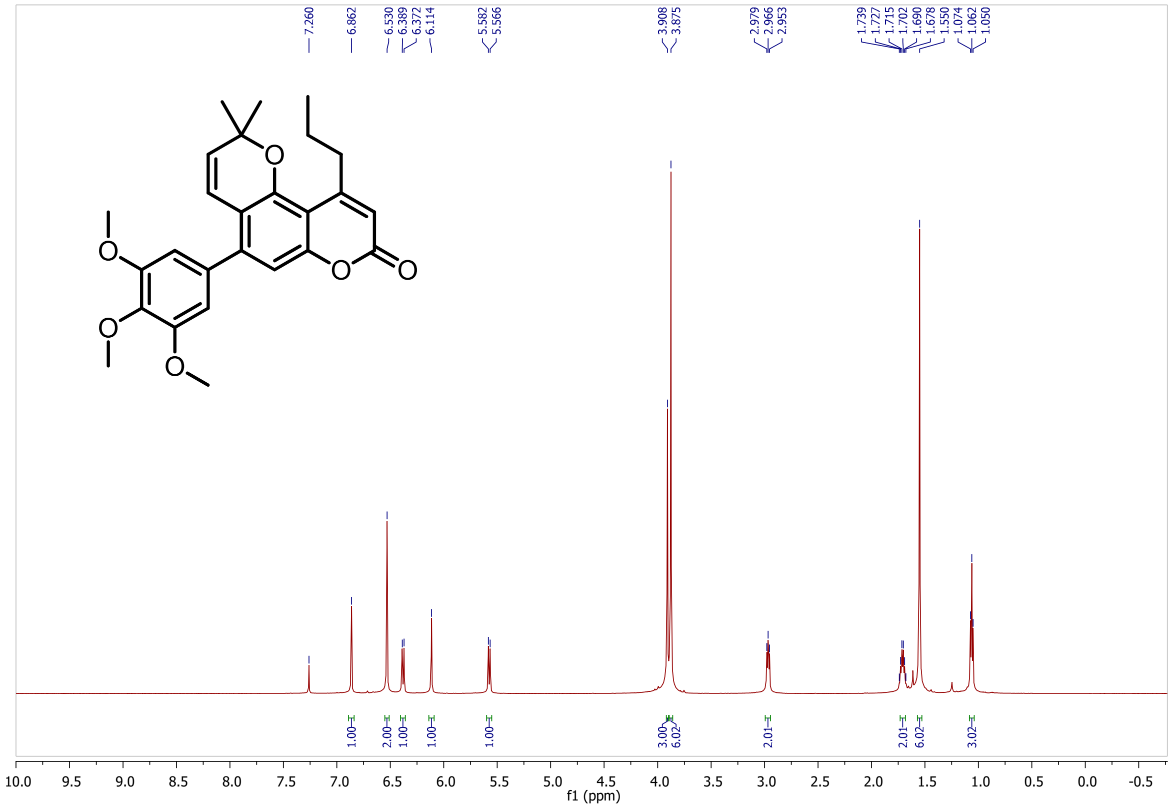

Supplementary Figure S38. ^{1}**H NMR spectrum of 7f**

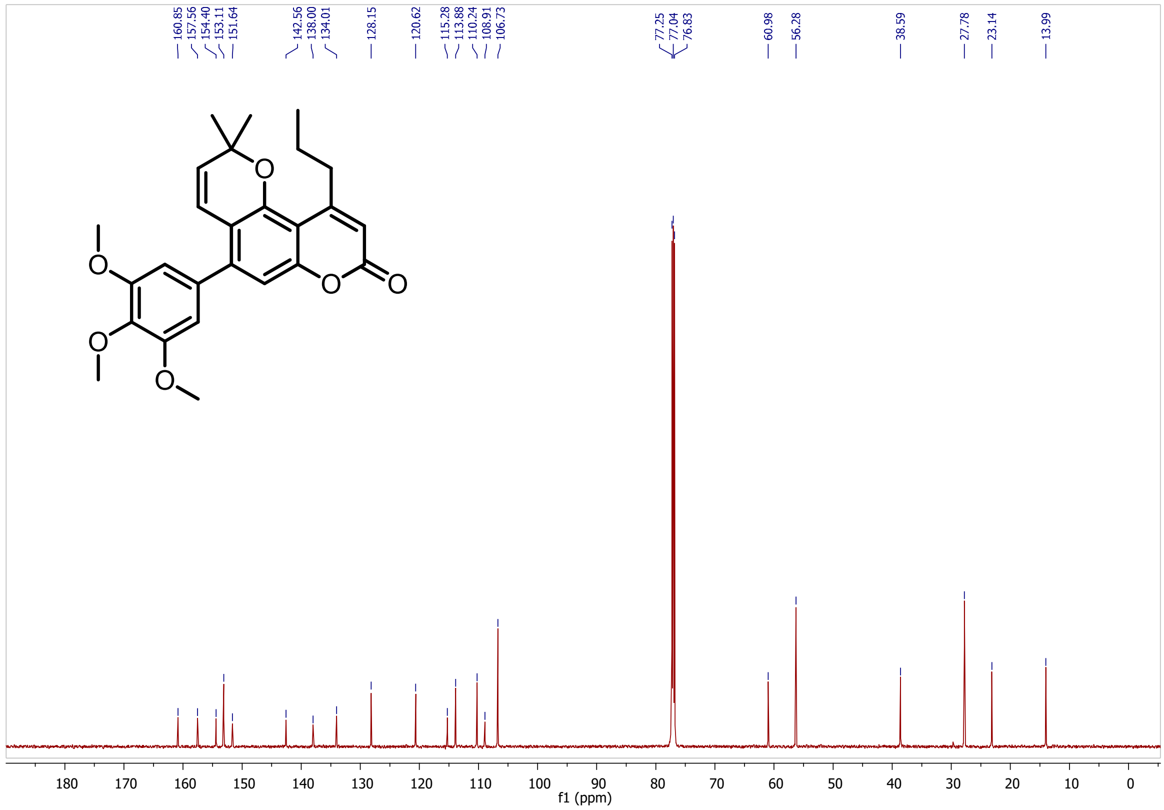

Supplementary Figure S39. ^{13}C NMR spectrum of 7f

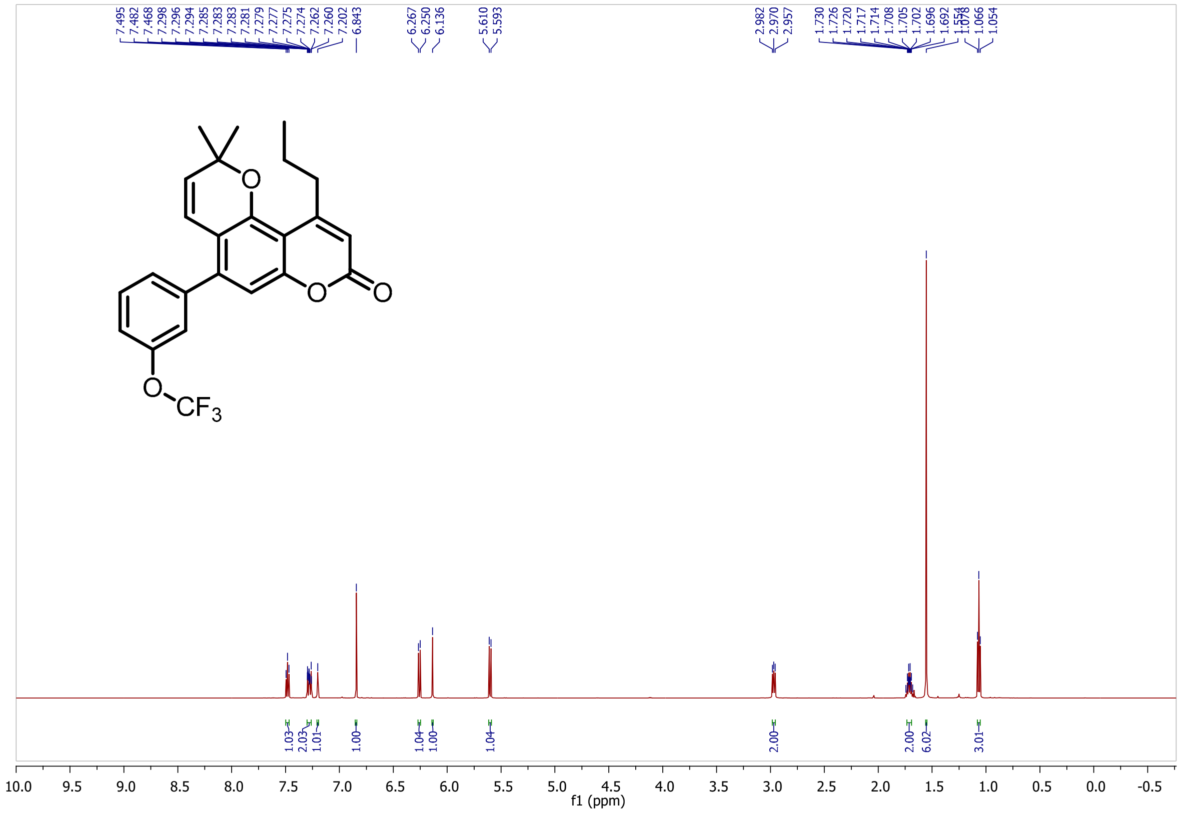

Supplementary Figure S40. ^{1}H NMR spectrum of 7g

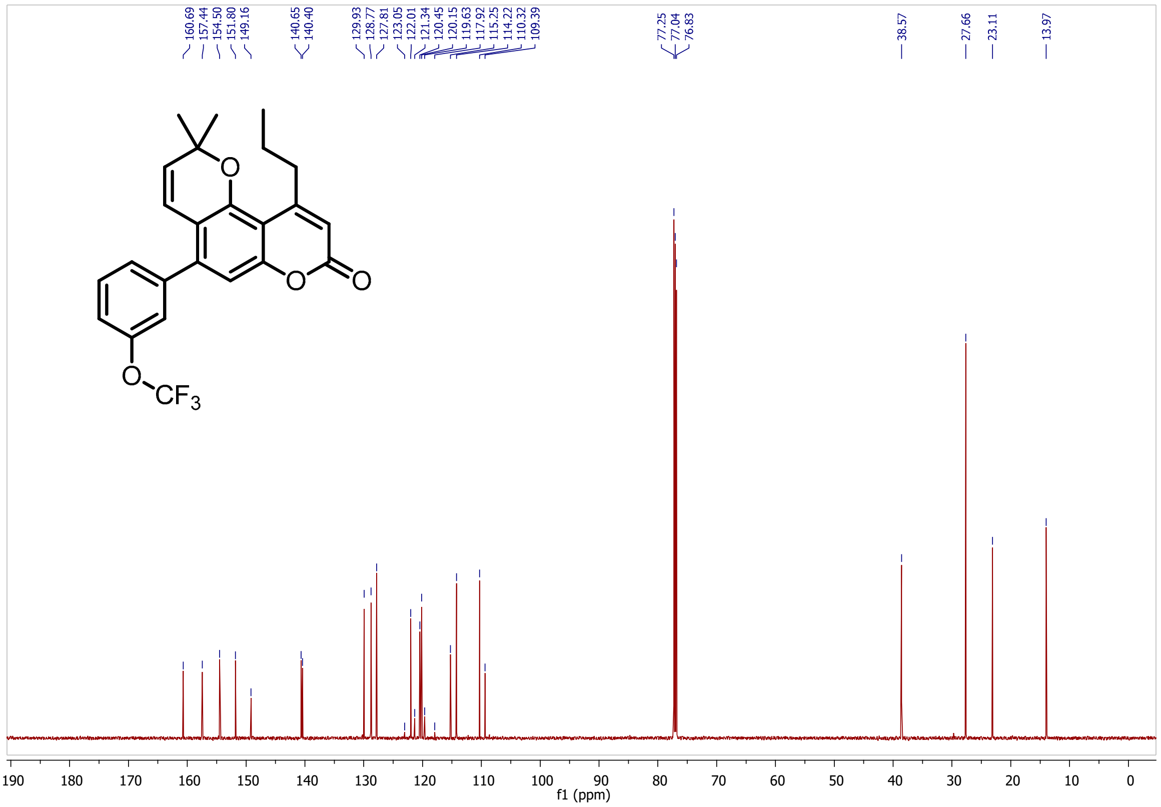

Supplementary Figure S41. ^{13}**C NMR spectrum of 7g**

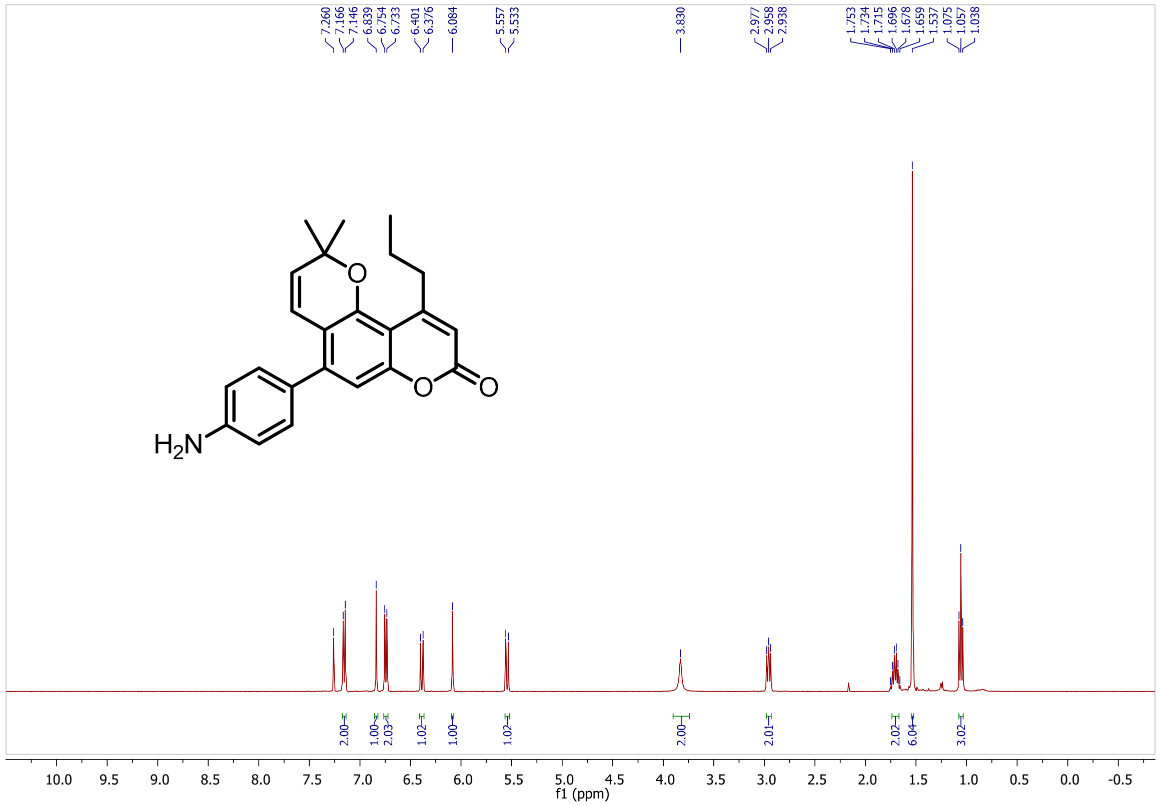

Supplementary Figure S42. ^{1}**H NMR spectrum of 7h**

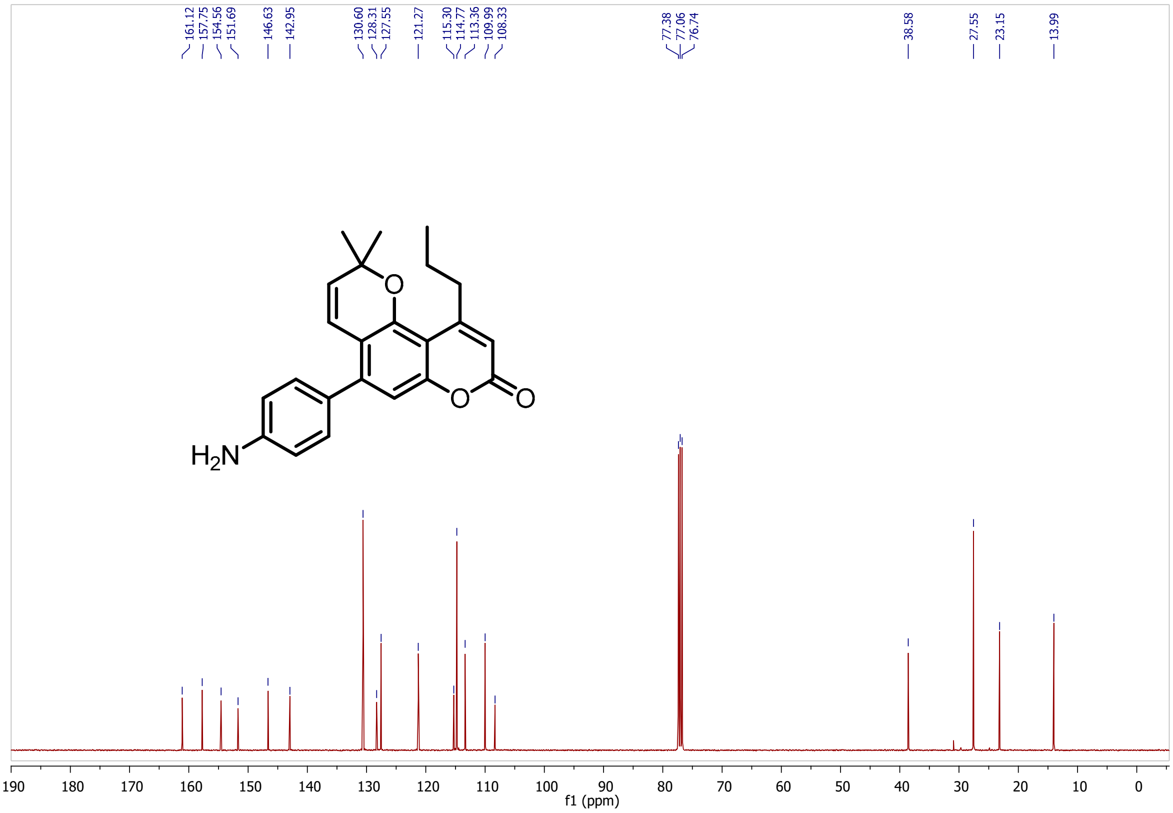

Supplementary Figure S43. ^{13}C NMR spectrum of 7h

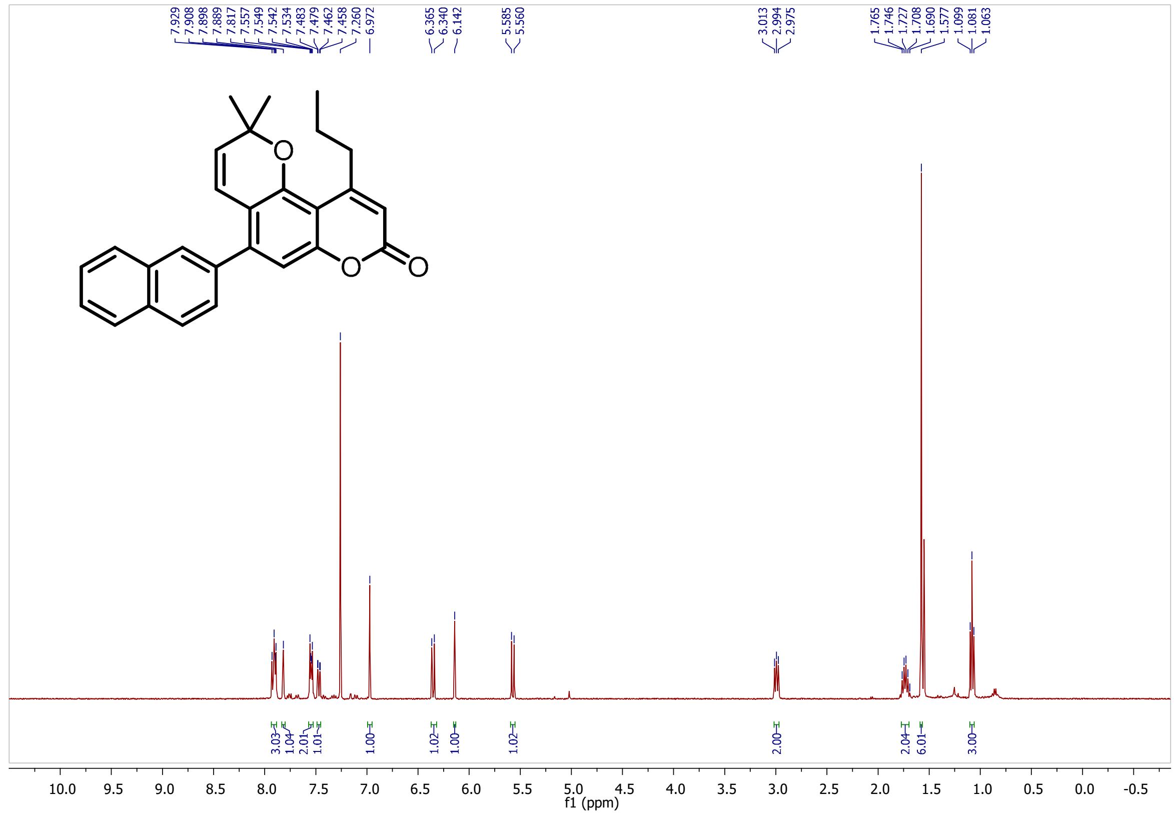

Supplementary Figure S44. ^{1}H NMR spectrum of 7i

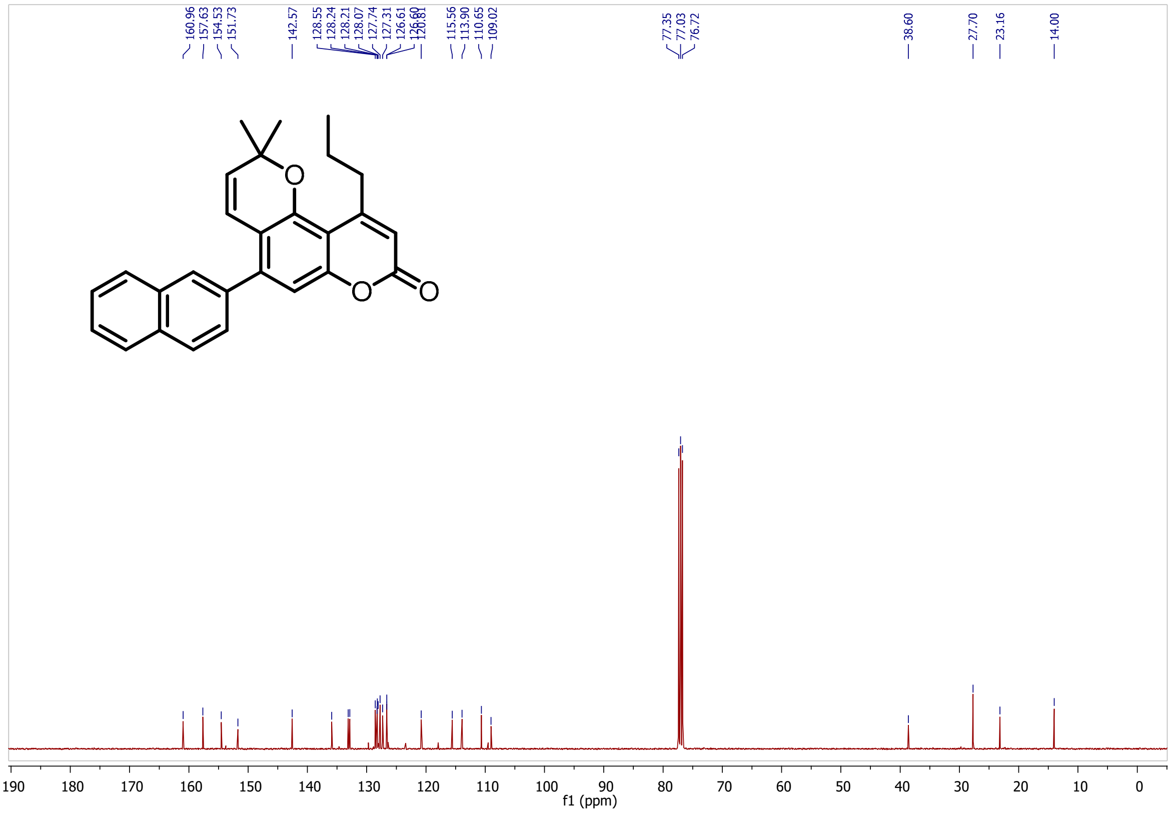

Supplementary Figure S45. ^{13}C NMR spectrum of 7i

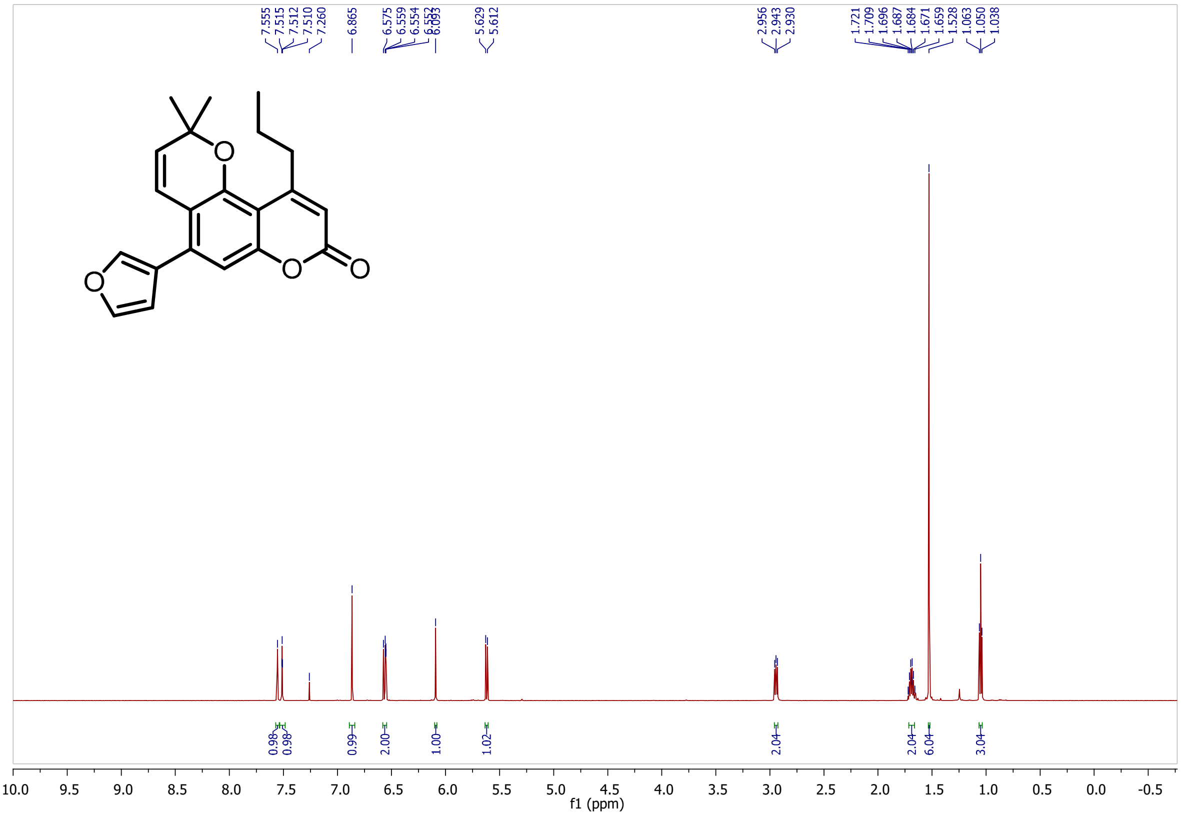

Supplementary Figure S46. ^{1}H NMR spectrum of 7g

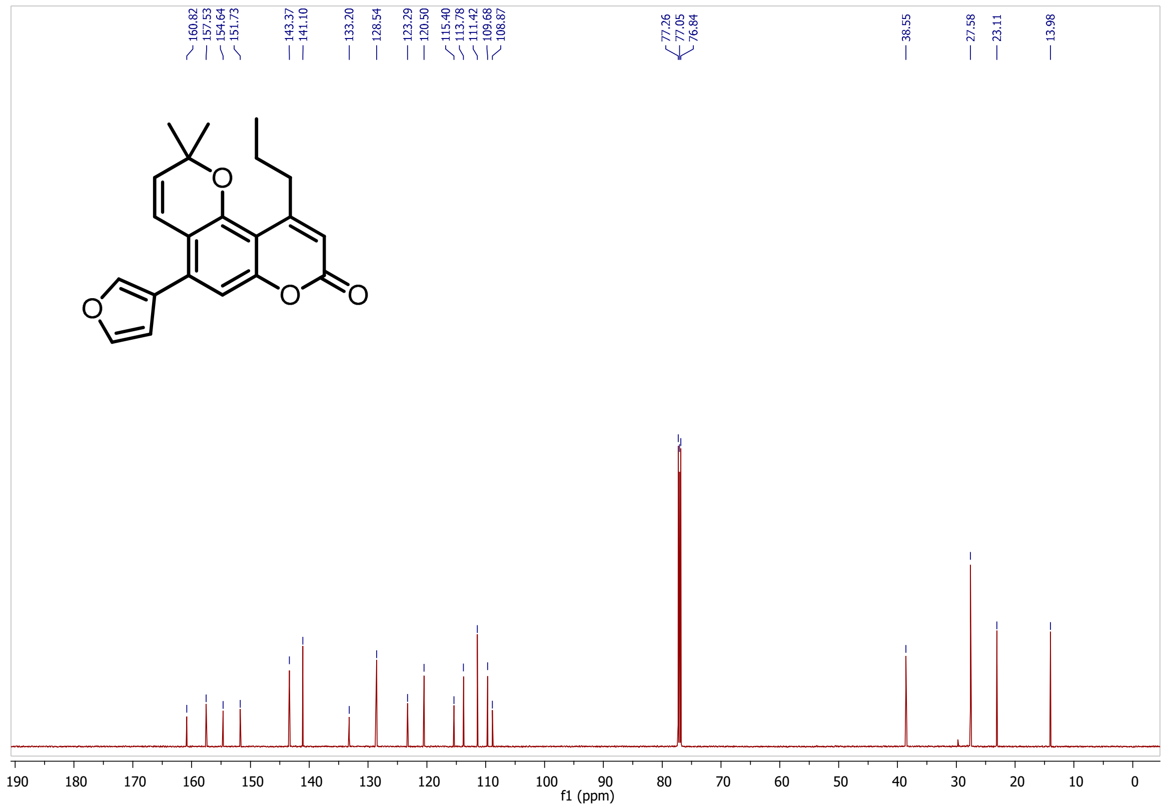

Supplementary Figure S47. ^{13}C NMR spectrum of 7g

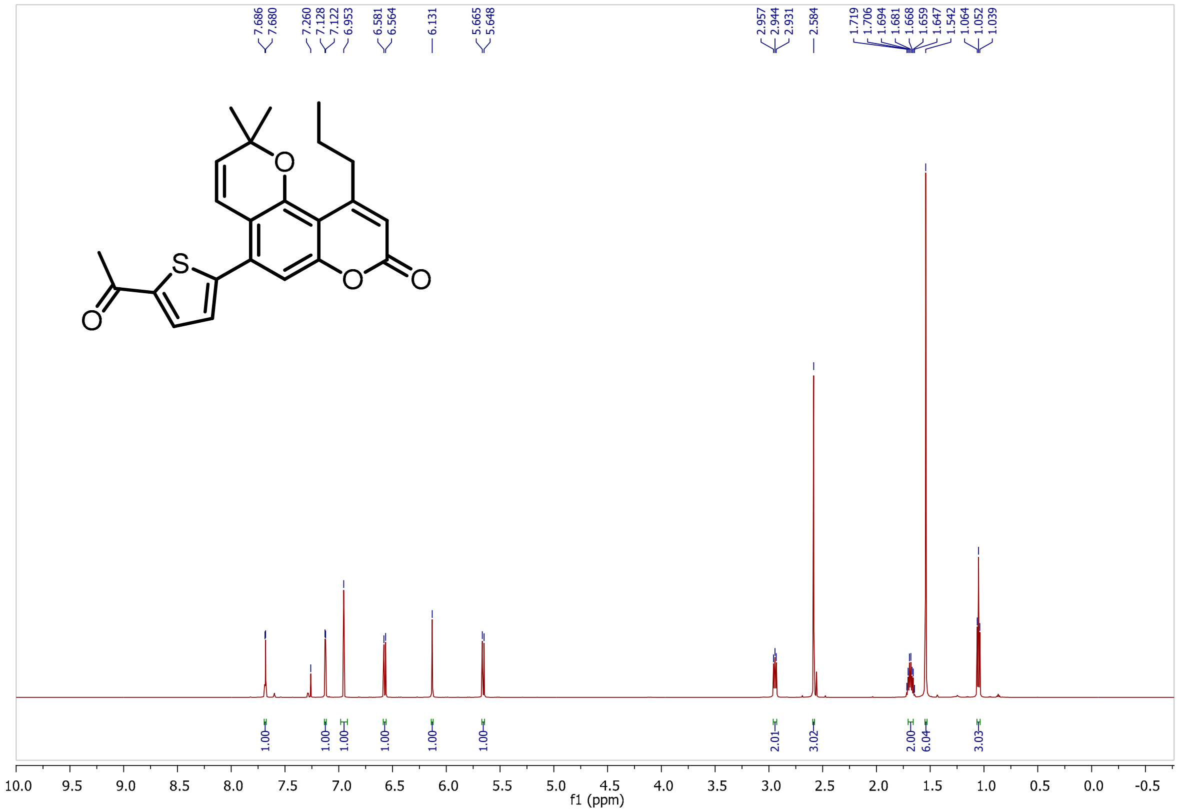

Supplementary Figure S48. ^{1}H NMR spectrum of 7k

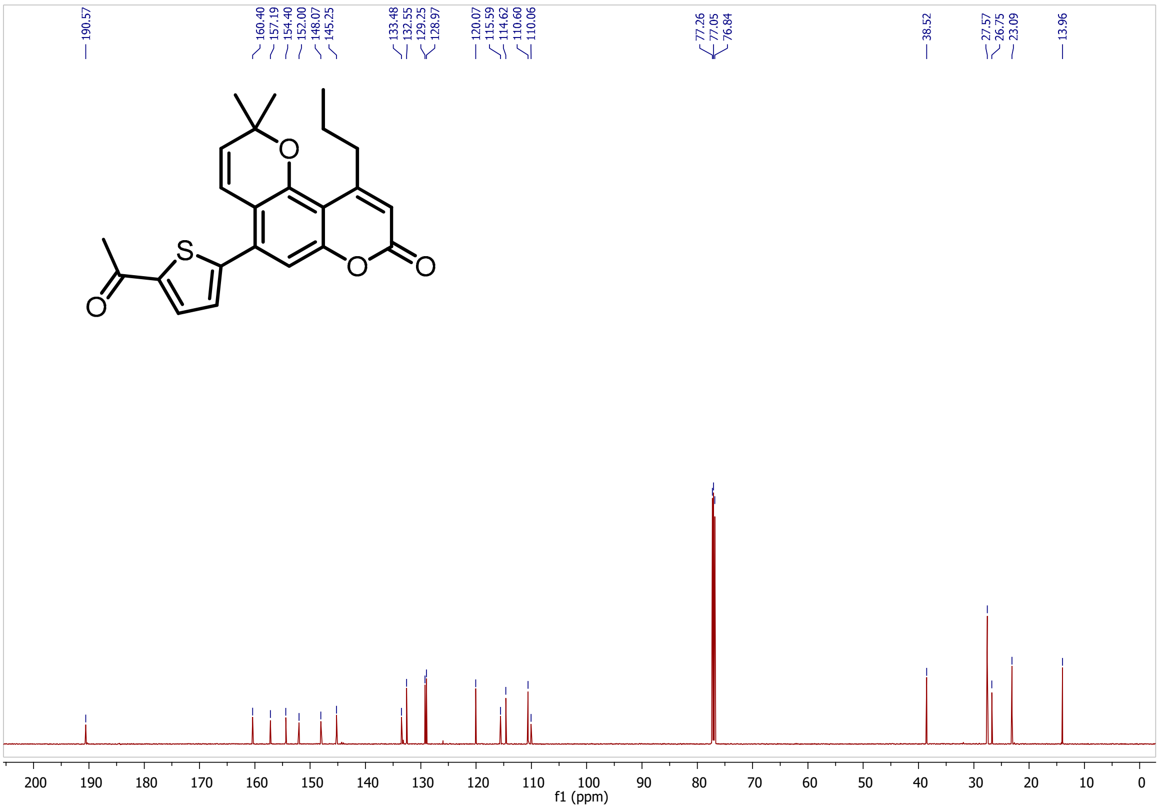

Supplementary Figure S49. ^{13}**C NMR spectrum of 7k**

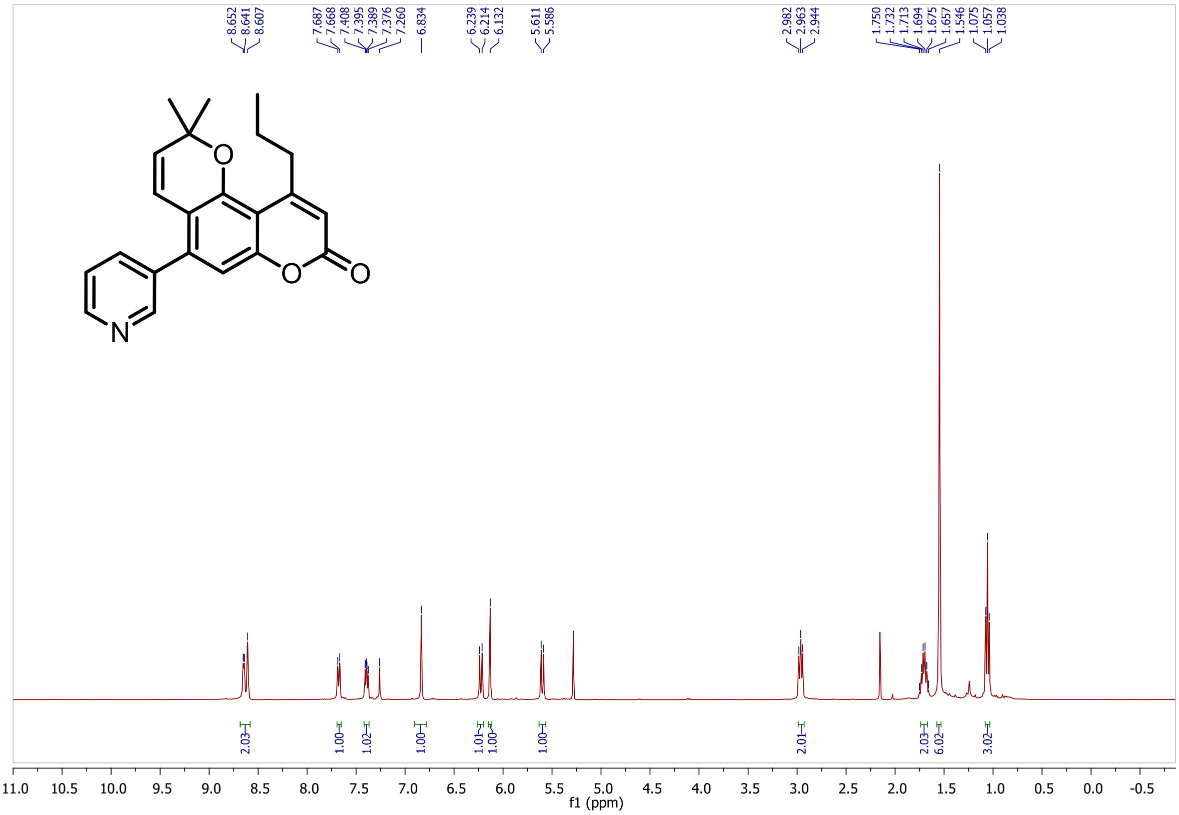

Supplementary Figure S50. ^{1}**H NMR spectrum of 7l**

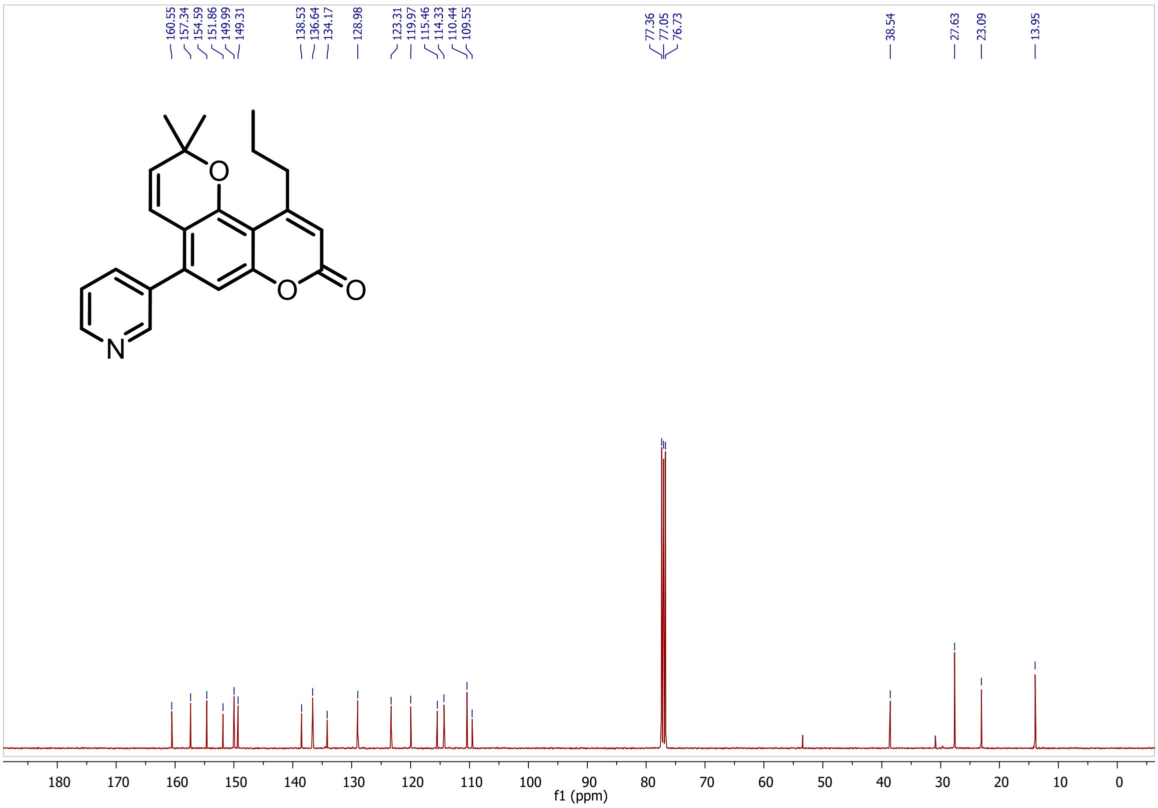

Supplementary Figure S51. ^{13}**C NMR spectrum of 7l**

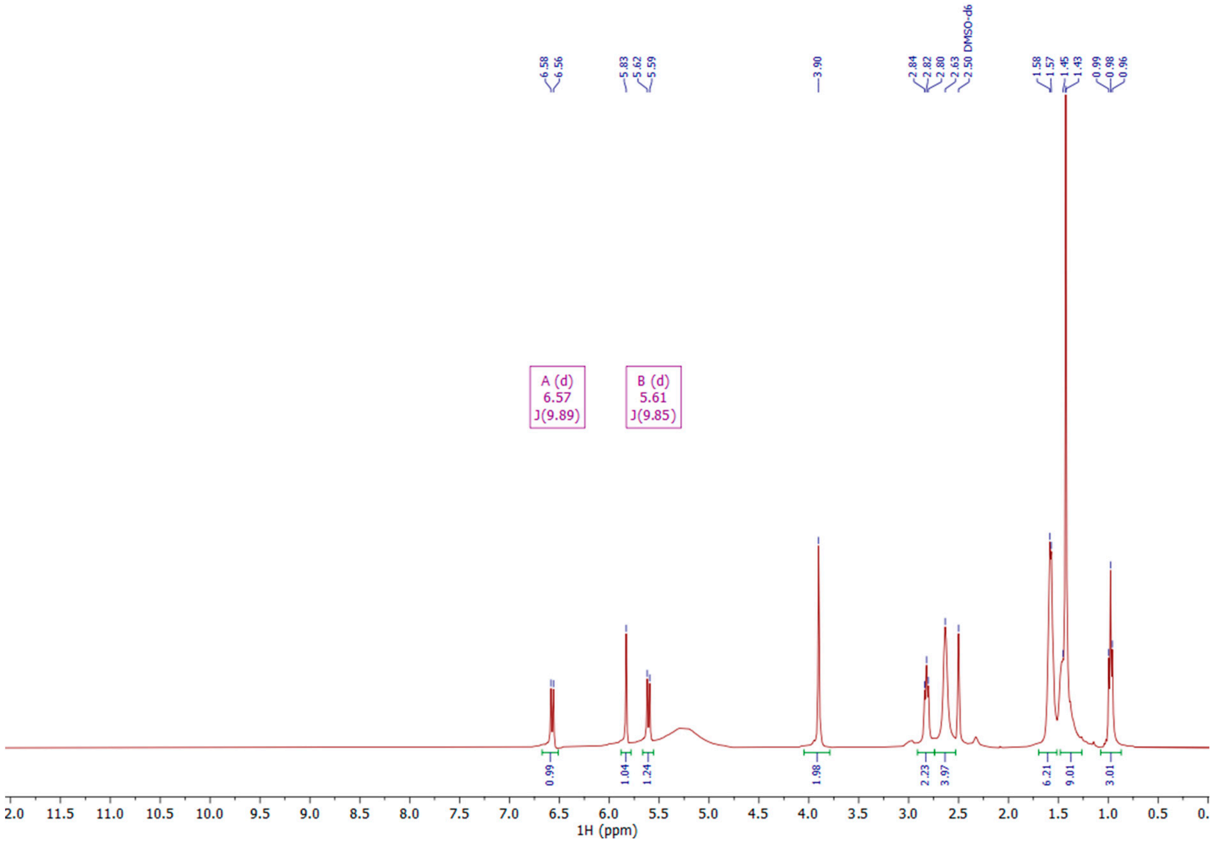

Supplementary Figure S52. ^{1}**H NMR spectrum of 9a**

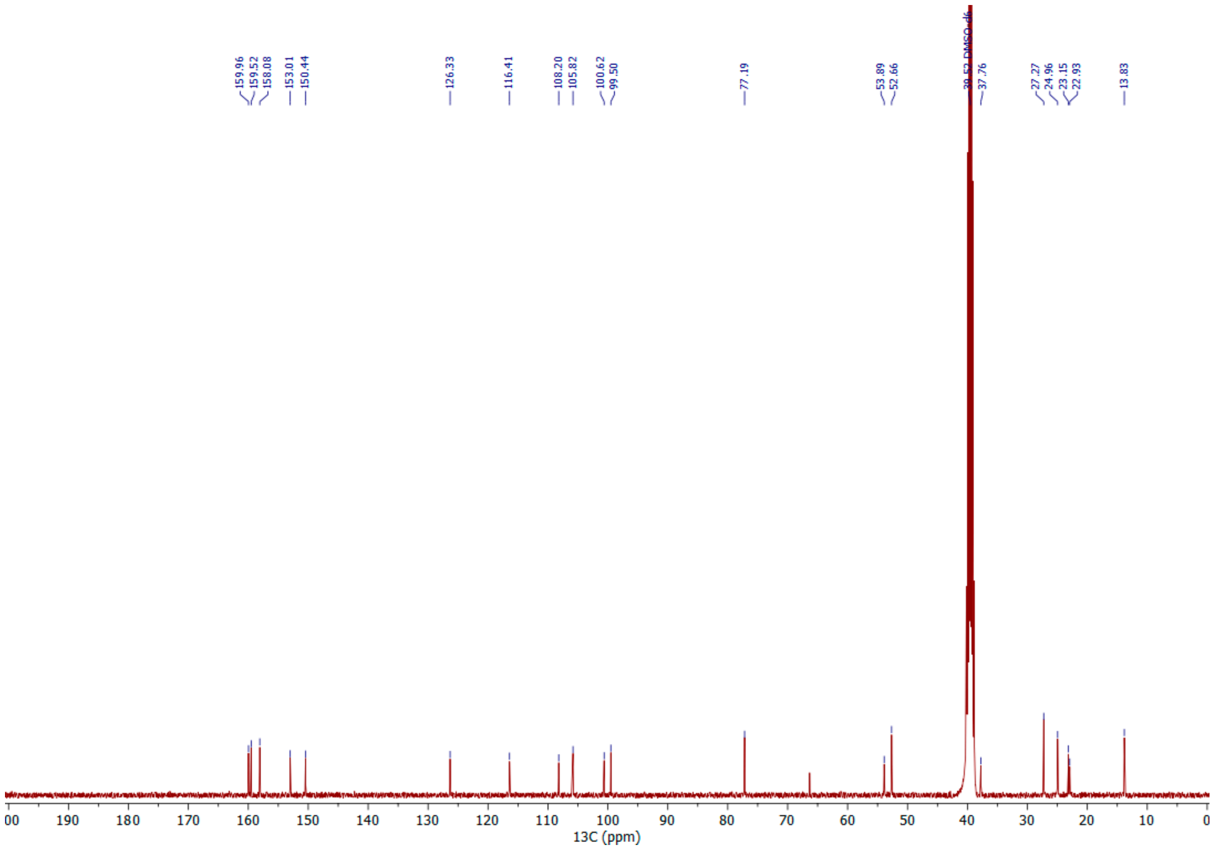

Supplementary Figure S53. ^{13}**C NMR spectrum of 9a**

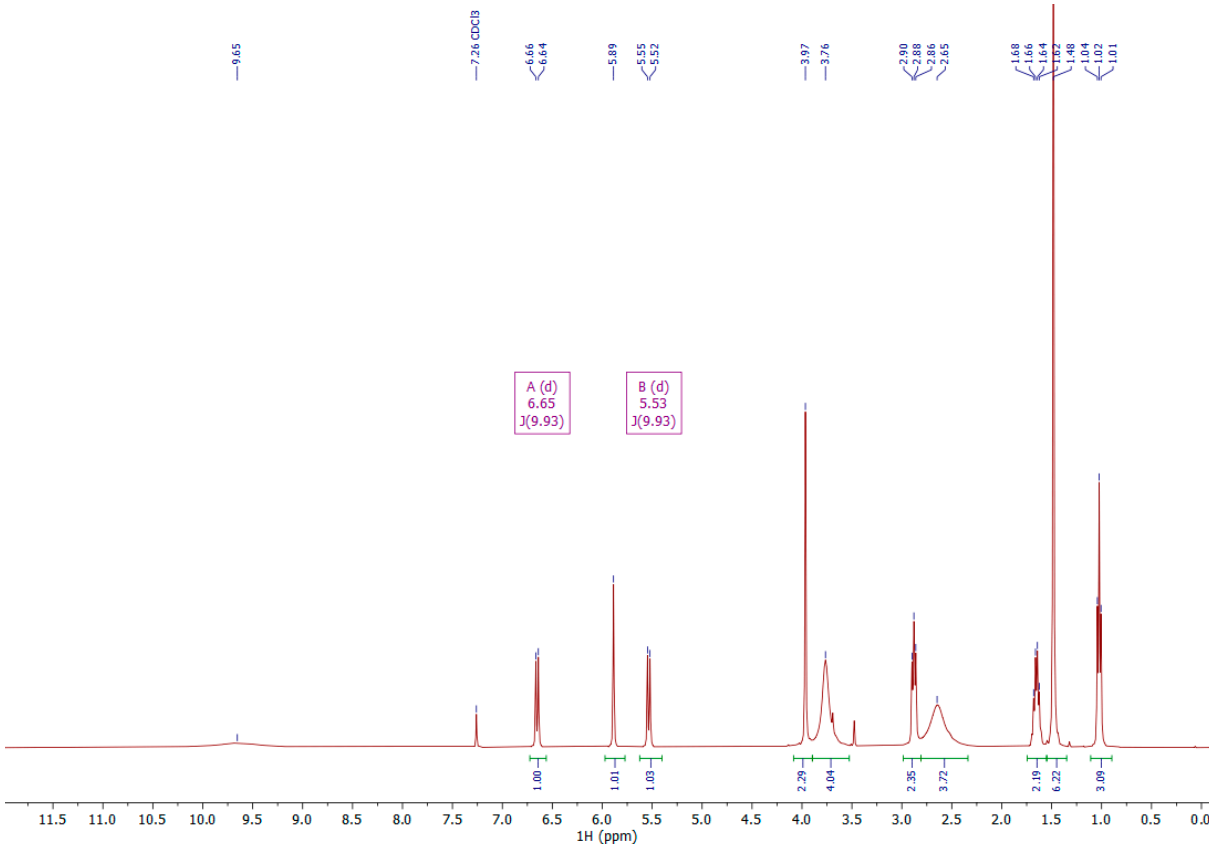

Supplementary Figure S54. ^{1}**H NMR spectrum of 9b**

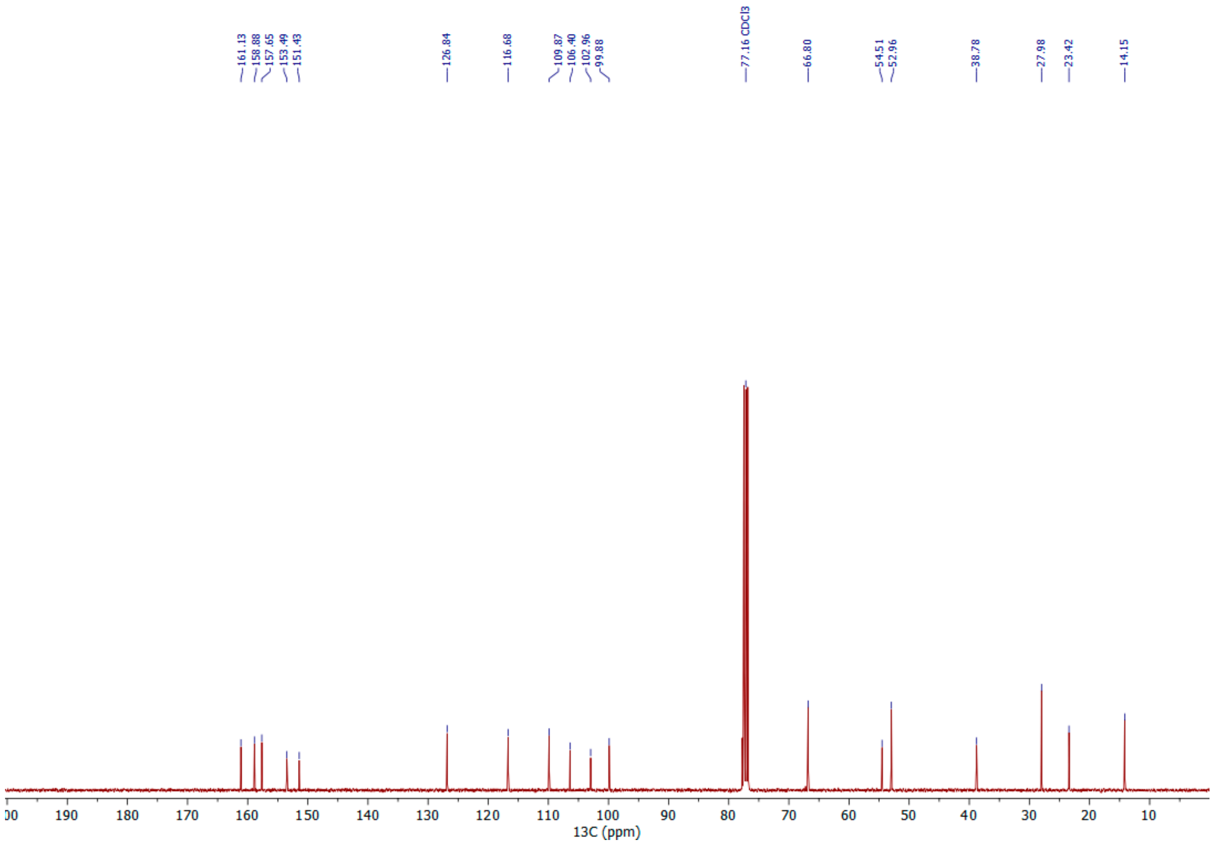

Supplementary Figure S55. ^{13}**C NMR spectrum of 9b**

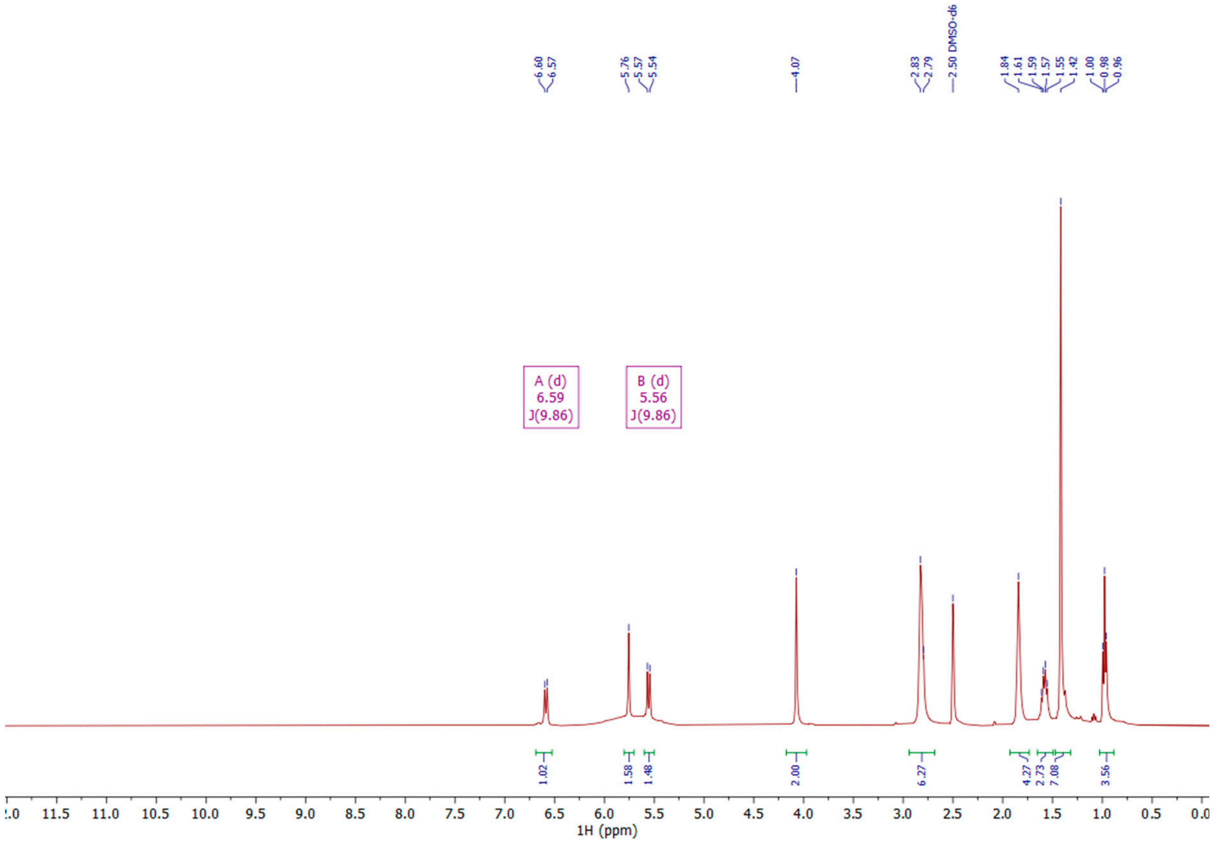

Supplementary Figure S56. ^{1}**H NMR spectrum of 9c**

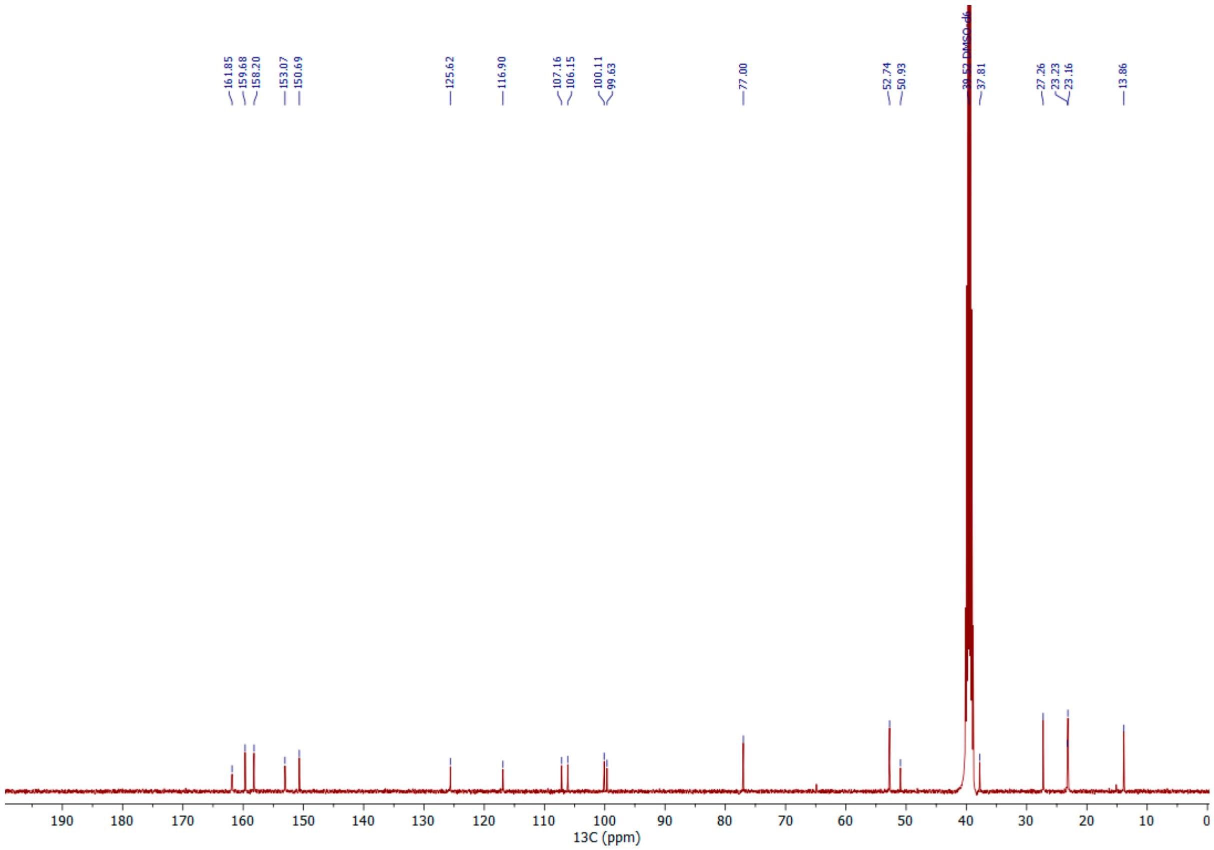

Supplementary Figure S57. ^{13}C NMR spectrum of 9c

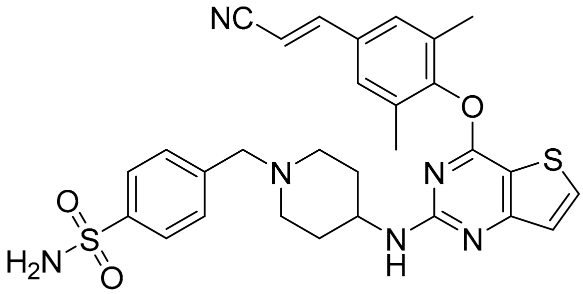

Supplementary Figure S58. Structure of CHEMBL4094163

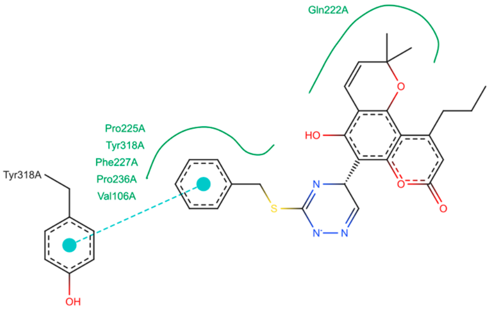

4d

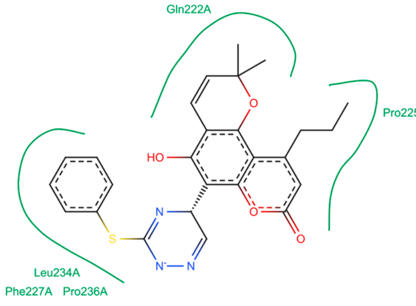

4e

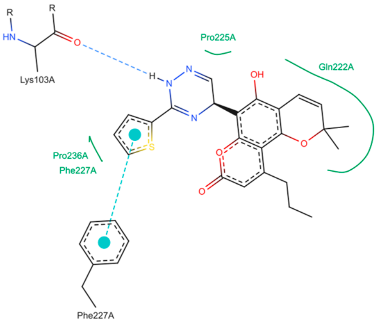

4g

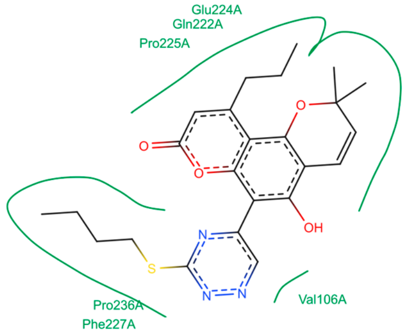

5b

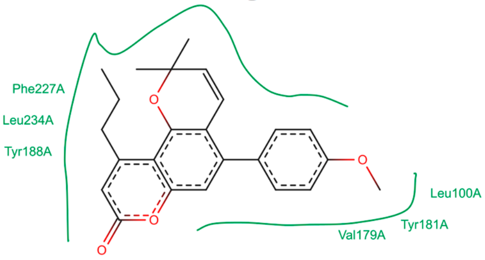

7e

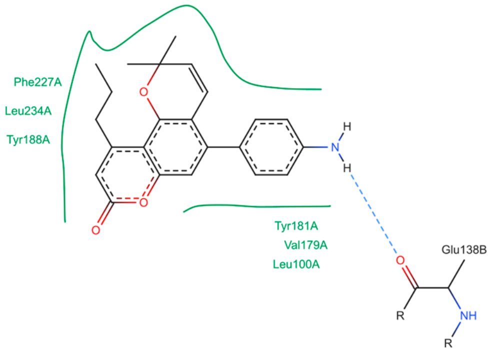

7h

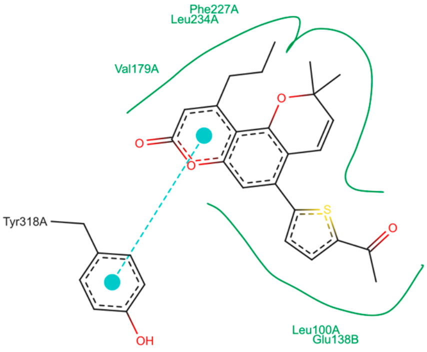

7k

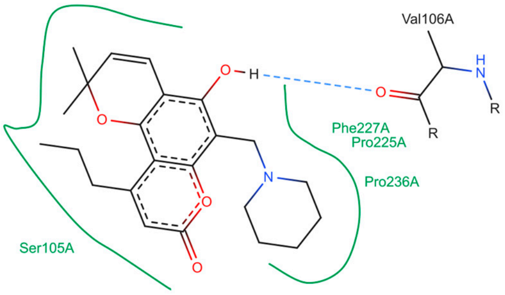

9a

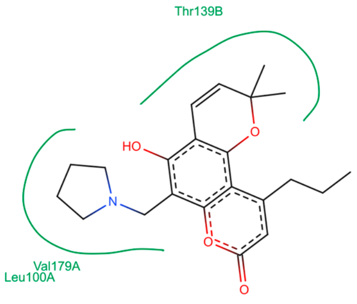

9c

Supplementary Figure S59. Two-dimensional maps of non-covalent interactions for the top-1 positions of docked ligands in HIV-RT non-nucleoside binding site

Supplementary Table S1. The results of docking of the known non-nucleoside inhibitors of HIV-1 RT WT

No	Structure	Jamda score	IC_{50}, nM	References
1	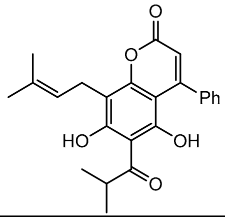	-2.39	2500	
2	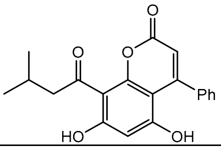	-2.79	6900	
3	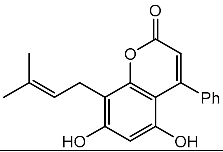	-2.37	11200	[30]
4	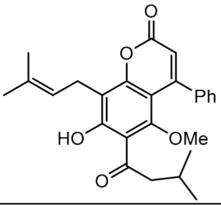	-2.60	22200	
5	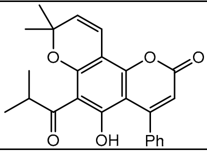	-1.95	32100	
6	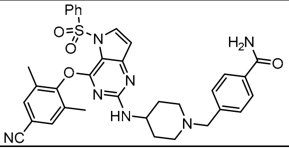	-3.36	195	[31]
7	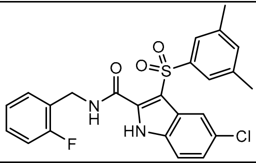	-3.23	1115	[32]
8	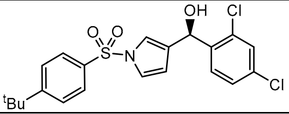	-3.13	180	[33]
9	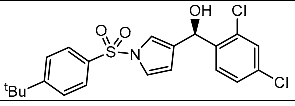	-2.64	6400	[34]
10	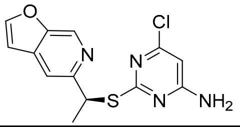	-2.51	24	[35]
11	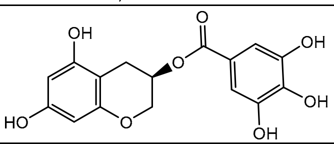	-2.50	3800	[36]
	Pearson coefficient	0.66		
	Spearman coefficient	0.54		
